# Estimates of the global, regional, and national morbidity, mortality, and aetiologies of lower respiratory tract infections in 195 countries: a systematic analysis for the Global Burden of Disease Study 2015

**DOI:** 10.1016/S1473-3099(17)30396-1

**Published:** 2017-11

**Authors:** GBD 2015 LRI Collaborators, Christopher Troeger, Mohammad Forouzanfar, Puja C Rao, Ibrahim Khalil, Alexandria Brown, Scott Swartz, Nancy Fullman, Jonathan Mosser, Robert L Thompson, Robert C Reiner, Amanuel Abajobir, Noore Alam, Mulubirhan Assefa Alemayohu, Azmeraw T Amare, Carl Abelardo Antonio, Hamid Asayesh, Euripide Avokpaho, Aleksandra Barac, Muktar A Beshir, Dube Jara Boneya, Michael Brauer, Lalit Dandona, Rakhi Dandona, Joseph R A Fitchett, Tsegaye Tewelde Gebrehiwot, Gessessew Buggsa Hailu, Peter J Hotez, Amir Kasaeian, Tawfik Khoja, Niranjan Kissoon, Luke Knibbs, G Anil Kumar, Rajesh Kumar Rai, Hassan Magdy Abd El Razek, Muktar S K Mohammed, Katie Nielson, Eyal Oren, Abdalla Osman, George Patton, Mostafa Qorbani, Hirbo Shore Roba, Benn Sartorius, Miloje Savic, Mika Shigematsu, Bryan Sykes, Soumya Swaminathan, Roman Topor-Madry, Kingsley Ukwaja, Andrea Werdecker, Naohiro Yonemoto, Maysaa El Sayed Zaki, Stephen S Lim, Mohsen Naghavi, Theo Vos, Simon I Hay, Christopher J L Murray, Ali H Mokdad

## Abstract

**Background:**

The Global Burden of Diseases, Injuries, and Risk Factors (GBD) Study 2015 provides an up-to-date analysis of the burden of lower respiratory tract infections (LRIs) in 195 countries. This study assesses cases, deaths, and aetiologies spanning the past 25 years and shows how the burden of LRI has changed in people of all ages.

**Methods:**

We estimated LRI mortality by age, sex, geography, and year using a modelling platform shared across most causes of death in the GBD 2015 study called the Cause of Death Ensemble model. We modelled LRI morbidity, including incidence and prevalence, using a meta-regression platform called DisMod-MR. We estimated aetiologies for LRI using two different counterfactual approaches, the first for viral pathogens, which incorporates the aetiology-specific risk of LRI and the prevalence of the aetiology in LRI episodes, and the second for bacterial pathogens, which uses a vaccine-probe approach. We used the Socio-demographic Index, which is a summary indicator derived from measures of income per capita, educational attainment, and fertility, to assess trends in LRI-related mortality. The two leading risk factors for LRI disability-adjusted life-years (DALYs), childhood undernutrition and air pollution, were used in a decomposition analysis to establish the relative contribution of changes in LRI DALYs.

**Findings:**

In 2015, we estimated that LRIs caused 2·74 million deaths (95% uncertainty interval [UI] 2·50 million to 2·86 million) and 103·0 million DALYs (95% UI 96·1 million to 109·1 million). LRIs have a disproportionate effect on children younger than 5 years, responsible for 704 000 deaths (95% UI 651 000–763 000) and 60.6 million DALYs (95ÙI 56·0–65·6). Between 2005 and 2015, the number of deaths due to LRI decreased by 36·9% (95% UI 31·6 to 42·0) in children younger than 5 years, and by 3·2% (95% UI −0·4 to 6·9) in all ages. Pneumococcal pneumonia caused 55·4% of LRI deaths in all ages, totalling 1 517 388 deaths (95% UI 857 940–2 183 791). Between 2005 and 2015, improvements in air pollution exposure were responsible for a 4·3% reduction in LRI DALYs and improvements in childhood undernutrition were responsible for an 8·9% reduction.

**Interpretation:**

LRIs are the leading infectious cause of death and the fifth-leading cause of death overall; they are the second-leading cause of DALYs. At the global level, the burden of LRIs has decreased dramatically in the last 10 years in children younger than 5 years, although the burden in people older than 70 years has increased in many regions. LRI remains a largely preventable disease and cause of death, and continued efforts to decrease indoor and ambient air pollution, improve childhood nutrition, and scale up the use of the pneumococcal conjugate vaccine in children and adults will be essential in reducing the global burden of LRI.

**Funding:**

Bill & Melinda Gates Foundation.

## Introduction

Lower respiratory tract infections (LRIs) are a substantial public health problem and a leading cause of illness and death in people of all ages. Previous estimates found that in 2013, LRIs caused more than 2·6 million deaths worldwide, making them the fifth leading cause of death overall and the leading infectious cause of death in children younger than 5 years.^1^ The burden of LRIs is highest in areas of low sociodemographic status, populations that depend on solid fuels for cooking and heating, and in malnourished and immunoimpaired populations.^2^ Global efforts to reduce the burden of LRIs using different preventive and treatment strategies require timely information about the burden of LRIs, their risk factors, and associated pathogens.

Estimates of the burden of LRIs and their aetiologies are being produced annually as part of the Global Burden of Diseases, Injuries, and Risk Factors (GBD) Study, which provides a unique source for tracking trends in LRI-related morbidity and mortality. Results from the GBD study that quantify the burden of LRI will help to measure progress towards the Sustainable Development Goals, including Goal 3, which is to ensure healthy lives and wellbeing for people of all ages.^3^

**Research in context**

**Evidence before this study**

Lower respiratory tract infections are a leading cause of morbidity and mortality, particularly in children younger than 5 years, and the global burden has been estimated by several groups, including the Global Burden of Disease study (GBD). We build on previous GBD studies with updated data and methods. Updated cause-of-death data came from additional years of vital registration systems (2012–14; data from Jan 1, 1980, to Dec 31, 2015) and by searching PubMed and Google Scholar for “verbal autopsy” on March 3, 2015. Updated non-fatal and aetiology data came from a PubMed search on June 1, 2015, for “lower respiratory infections”, “bronchiolitis”, and “pneumonia”. Articles published between Jan 1, 2012, and Dec 31, 2015, were selected without language restrictions.

**Added value of this study**

This manuscript provides a comprehensive assessment of LRI burden based on GBD 2015, including newer and more robust evidence on the mortality, morbidity, and risk factors associated with LRIs, including four aetiologies, and is the first cause-specific description of LRI in a GBD study. In addition to descriptions of trends in morbidity and mortality, we use the Socio-demographic Index to relate changes in LRI burden to demographic transitions and assess the effect of changing population characteristics and risk factor exposure to decompose the trends in LRI burden.

**Implications of all the available evidence**

We show a decreasing burden of LRI in children younger than 5 years, but an increase in the burden in adults. Furthermore, we show the change in risk factor exposure globally, providing health professionals with valuable information needed to design and implement effective programmes and policies to reduce the burden of LRI. We also identify high-burden LRI regions that need more attention. Expanded use of the pneumococcal conjugate vaccine, interventions to improve under-5 nutrition, and a focus on appropriate case management could reduce the burden of LRI. Comprehensive and reliable data on LRI morbidity and mortality globally are still needed.

Here, we present results from the GBD study 2015, describing the burden of LRIs and four aetiologies (*Haemophilus influenzae* type B [Hib], *Streptococcus pneumoniae* [pneumococcal pneumonia], influenza, and respiratory syncytial virus [RSV]), covering deaths, episodes, disability-adjusted life-years (DALYs), risk factors contributing to the burden of LRIs, and the relationship between LRIs and social development for 195 countries from 1990 to 2015 for both sexes and by age.

## Methods

### Modelling overview

Details on the methods for GBD 2015 are available elsewhere.^4^, ^5^ Here, we give a brief description of the methods and estimation strategy for LRIs, defined as acute-onset physician-diagnosed pneumonia or bronchiolitis. We measure LRI burden using three metrics: deaths, episodes, and DALYs. DALYs are the sum of years of life lost (YLLs) because of premature death and years lived with disability (YLDs). We estimated mortality and morbidity separately. Flowcharts and a detailed description for each step of the estimation process are provided in the appendix pp 2–3. Input data, including information on sources used, and code for each step of the estimation process are available on the Global Health Data Exchange. All estimates are produced by year and by age, for both sexes, and for all 195 countries. Each step of the GBD 2015 LRI estimation process, including data sources, is documented in accordance with the Guidelines for Accurate and Transparent Health Estimates Reporting.^6^

We saved 1000 draws from a posterior distribution of each parameter, and we repeated each analysis 1000 times using these draws to retain uncertainty of every step and input parameters. The results are given as mean values with 95% uncertainty intervals (UIs) showing the 2·5 and 97·5 percentiles of the distribution.

### Mortality

The GBD Cause of Death database contains all available data from vital registration systems, surveillance systems, and verbal autopsies (summary in appendix p 4). We processed raw data to reconcile disparate coding schemes (such as the International Classification of Diseases 9 and 10), to redistribute poorly coded causes of death, and separate data by age and sex from tabulated cause lists.^7^

We estimated LRI mortality in the Cause of Death Ensemble model (CODEm) framework.^5^, ^8^ CODEm is a spatiotemporal modelling platform that produces a wide range of submodels from cause of death data and space–time covariates. Covariates were selected independently for each submodel using an algorithm that captures the relationships between the covariates and LRI mortality and provides a variety of plausible models (for full list of covariates, see appendix p 5). We assessed our LRI cause of death models using in-sample and out-of-sample predictive performance.

The sum of all cause-specific mortality models must be equal to the all-cause mortality estimate.^5^ We corrected LRI mortality estimates and estimates for other causes of mortality by rescaling them according to the uncertainty around the cause-specific mortality rate. This process is called CoDCorrect and ensures internal consistency between causes of death and respects the all-cause mortality envelope.^5^

### Morbidity

LRIs were defined as clinician-confirmed or radiologically confirmed pneumonia or bronchiolitis and were divided into moderate and severe or very severe episodes on the basis of WHO case definitions for pneumonia.^9^ Input data were derived from a systematic literature review of cross-sectional and cohort studies, hospital inpatient and outpatient data, health-care utilisation data (USA only), population-representative surveys, and excess mortality from the GBD 2015 cause of death estimates for LRI (appendix pp 6–7).

LRI morbidity (incidence, prevalence, and remission) was modelled using DisMod-MR version 2.1 (DisMod), a Bayesian, hierarchical, mixed-effects meta-regression platform.^4^, ^10^, ^11^ DisMod adjusts for variations in study methods between data sources and enforces consistency between data for the different parameters such as incidence and prevalence. Incidence, prevalence, remission, and excess mortality were related in a compartmental model of disease progression. Epidemiological data on LRI burden were analysed through a geographical cascade from a global level, at which fixed effects for covariates are established, to the most detailed geographic estimation level, which was either the national or subnational level. Model estimates from higher levels of the cascade were used as priors in analyses of lower levels. Random effects exist for each geographic estimation level. Geospatial priors, space–time covariates, random effects, and input data predicted incidence and prevalence of LRI episodes. Input data were adjusted in DisMod during the modelling process to meet our standard case definition using study-level binary covariates. These covariates described the source of the data and accounted for hospital-based, inpatient, and self-reported sources (appendix p 8).

DALYs are the sum of YLLs and YLDs and represent the cumulative burden of disease due to LRI.^12^ To estimate the YLDs from LRIs, we used a disability weight for each severity level (moderate and severe or very severe) and the proportion of cases that fall into each severity level (appendix p 7).

### Aetiologies

We estimated LRI aetiologies separately from overall LRI mortality and morbidity using two distinct counterfactual modelling strategies to calculate population attributable fractions (PAFs) for influenza, RSV, Hib, and pneumococcal pneumonia. The PAF is the proportional reduction in LRI morbidity or mortality that would be observed if the exposure to the pathogen were zero. We did not attribute aetiologies to neonatal pneumonia cases or deaths because of an absence of reliable data in this age group, and we did not consider Hib in age groups older than 5 years for the same reason.

We used a vaccine probe design to estimate the PAF for pneumococcal pneumonia and Hib by first calculating the ratio of vaccine effectiveness against non-specific pneumonia to pathogen-specific pneumonia at the study level.^13^, ^14^, ^15^ We then adjusted this estimate by vaccine coverage and vaccine effectiveness to estimate country-specific and year-specific PAF values.^16^, ^17^ We did not account for herd immunity in our estimates. Equations and more about these calculations are provided in the appendix (pp 9–10).

For Hib, we assumed that the vaccine efficacy against invasive Hib disease is the same as against Hib pneumonia. However, we did not make the same assumption for pneumococcal pneumonia because a study of pneumococcal conjugate vaccine (PCV) found that the vaccine efficacy against invasive pneumococcal disease might be significantly higher than against pneumococcal pneumonia.^18^ We used a ratio of efficacy against pneumococcal pneumonia to invasive pneumococcal disease from this study to adjust estimates of vaccine efficacy against invasive pneumococcal disease from the other studies. We used separate pneumococcal pneumonia and Hib age distributions, modelled in DisMod, to establish the PAF by age. Finally, geography and year PAFs were estimated using vaccine coverage modelled estimates.

Influenza and RSV were estimated by calculating an attributable fraction that relates the odds ratio (OR) of LRI given pathogen detection^19^ and proportion of LRI episodes that test positive for influenza or RSV.^20^

$$PAF=Proportion*(1-\frac{1}{\text{OR}})$$

We conducted a systematic literature review of the proportion of LRI cases that test positive for influenza and RSV and used the meta-regression tool DisMod to estimate the proportion of people with LRI who are positive for influenza and RSV, separately, by location, year, age, and sex.

Different PAFs were measured for non-fatal and fatal LRI episodes. Fatal PAFs were adjusted using a scalar from the DisMod proportion models that represents the relative frequency of detection in inpatient versus non-inpatient sample populations. In the absence of aetiological data from fatal cases of LRI after death, we assumed that episodes of LRI requiring hospital admission were a reasonable proxy of severe and fatal episodes. Mortality is lower in patients with viral pneumonia than in those with pneumonia with bacterial causes. Therefore, we adjusted the fatal PAF estimates by establishing the ratio of case fatality in viral to bacterial causes of pneumonia from hospital data coded specifically to these causes, representing the relative fatality in people who were treated (appendix p 13).

### Changes in burden with development

On the basis of methods used to construct the Human Development Index, GBD 2015 used the Socio-demographic Index (SDI), a summary measure of a country's development based on lag-distributed income per capita, average educational attainment, and total fertility rate.^5^, ^21^ We used the SDI to show how changes in under-5 LRI mortality and incidence are related to changes in development. We fitted a least-squares regression using a cubic spline of the relationship of SDI to LRI mortality and incidence for each year at the most detailed geographic locations.

### Risk factor decomposition

Methods for risk factor attribution to LRI are described in detail elsewhere.^22^, ^23^ Briefly, risk factors followed a PAF counterfactual approach in which the prevalence of exposure was modelled from scientific literature and population-representative surveys, and the relative risk of LRI given a risk exposure was from published meta-analyses. We used the two leading risk groups for LRI DALYs from GBD 2015:^22^ air pollution (composed of household air pollution and ambient particulate matter^24^, ^25^) and childhood undernutrition (composed of underweight, wasted, and stunted^26^), in a decomposition analysis of the change in LRI DALYs from 2005 to 2015. This period was chosen to show recent changes. The decomposition had four factors that contribute interdependently to LRI burden: undernutrition exposure, air pollution, population growth, and population ageing. The remaining changes were considered part of the unexplained causes of LRIs. A combinatorial process established the relative contribution of each of these four factors to the change in LRI DALYs.^22^, ^27^

### Role of the funding source

The sponsor of the study had no role in study design, data collection, data analysis, data interpretation, or writing of the report. The corresponding author had full access to all the data in the study and had final responsibility for the decision to submit for publication.

## Results

**Figure 1:**
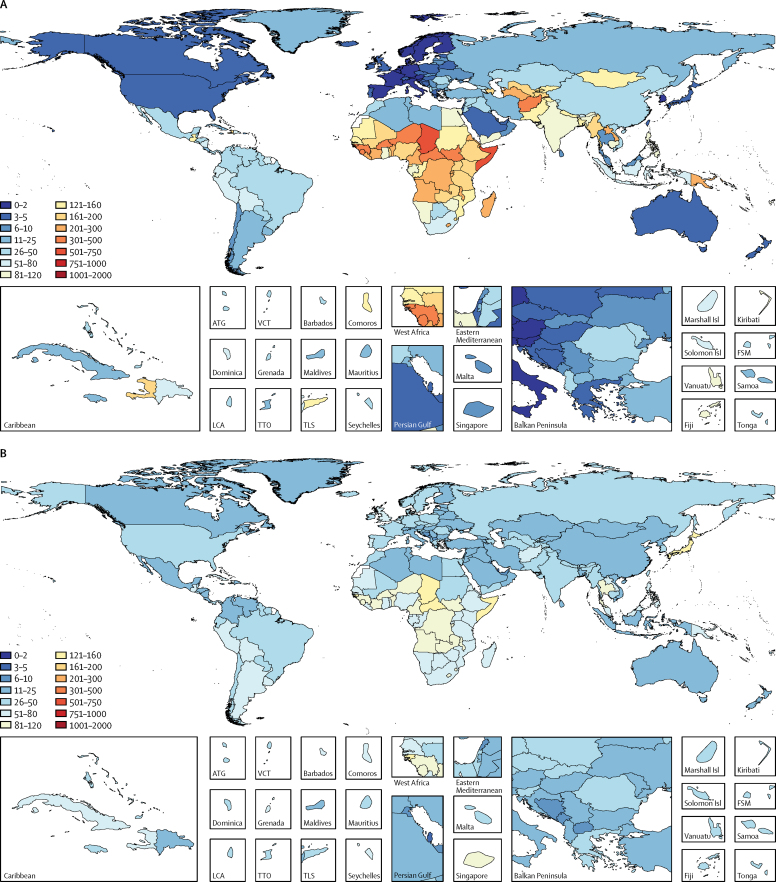

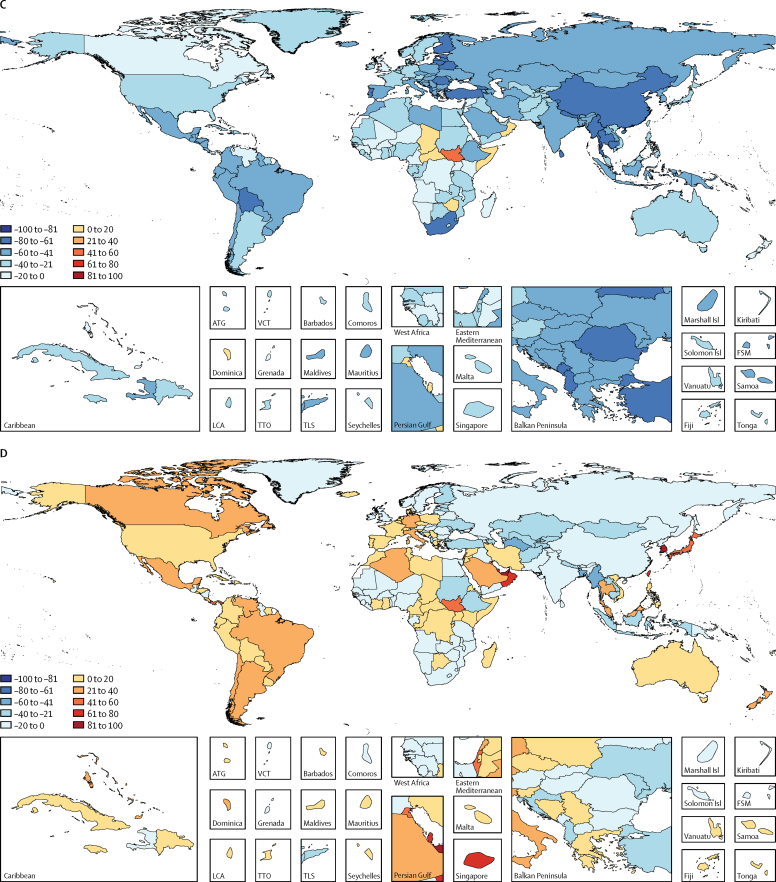
Global distribution of LRI mortality LRI mortality rate per 100 000 people in children younger than 5 years (A) and all ages (B) in 2015. Percent change in LRI deaths per 100 000 people between 2005 and 2015 in children younger than 5 years (C) and in all ages (D). LRI=lower respiratory tract infection. ATG=Antigua and Barbuda. VCT=Saint Vincent and the Grenadines. FSM=Federated States of Micronesia. LCA=Saint Lucia. TTO=Trinidad and Tobago. TLS=Timor-Leste.

At the global level, under-5 LRI mortality occurred in 104·8 children per 100 000 (95% UI 97·0–113·6) and varied by region and country (table 1, figure 1A). According to our estimates, the highest under-5 LRI mortalities were in sub-Saharan Africa, in Somalia (546·8 deaths per 100 000, 95% UI 404·5–716·4) and Chad (511·3 deaths per 100 000, 361·9–693·1; table 1), and the lowest were in Finland in western Europe (0·65 deaths per 100 000, 0·43–0·88; figure 1A). The greatest overall number of under-5 LRI deaths occurred in India (140 649 deaths, 95% UI 122 930–160 758) because of its large population (table 1). The under-5 LRI mortality was nearly the same in males and females at the global level, but in south Asia, it was 1·2-times higher in girls than in boys (1·22 times in India and 1·24 times in Pakistan).

**Table 1:** Episodes, DALYs, and deaths attributable to lower respiratory tract infections in 2015, by country

	**Children younger than 5 years**						**All ages**					
	Deaths			Episodes	DALYs		Deaths			Episodes	DALYs	
	Total number	Number per 100 000	Percent change 2005–15	Number (× 10^5^)	Number (× 10^5^)	Percent change 2005–15	Total number	Number per 100 000	Percent change 2005–15	Number (× 10^5^)	Number (× 10^5^)	Percent change 2005–15
**Global**	**703 917·9 (651 385·4 to 763 038·7)**	**104·8 (97·0 to 113·6)**	**−36·9 (−42·0 to −31·6)**	**1017·59 (900·03 to 1144·66)**	**605·53 (560·2 to 656·12)**	**−36·8 (−41·9 to −31·6)**	**2 736 714·2 (2 500 318·4 to 2 860 842·8)**	**37·1 (33·9 to 38·8)**	**−3·2 (−6·9 to 0·4)**	**2917·68 (2762·52 to 3070·13)**	**1030·49 (961·28 to 1090·79)**	**−23·8 (−28·2 to −19·4)**
**Central Europe, eastern Europe, and central Asia**	**15 935·5 (13 771·1 to 18 307·4)**	**57·6 (49·8 to 66·2)**	**−42·0 (−50·7 to −32·4)**	**39·11 (34·30 to 44·52)**	**13·74 (11·9 to 15·76)**	**−41·9 (−50·6 to −32·3)**	**103 530·3 (98 694·4 to 108 661·1)**	**24·7 (23·6 to 26·0)**	**−15·6 (−21·2 to −9·4)**	**139·02 (132·15 to 146·25)**	**36·21 (34·13 to 38·63)**	**−30·3 (−35·5 to −24·3)**
Albania	85·0 (55·9 to 121·3)	45·9 (30·2 to 65·6)	−60·8 (−76·2 to −39·8)	0·36 (0·31 to 0·42)	0·07 (0·05 to 0·1)	−60·7 (−76·0 to −39·7)	423·6 (336·3 to 562·9)	14·6 (11·6 to 19·4)	−19·7 (−36·1 to 0·2)	0·91 (0·85 to 0·97)	0·13 (0·1 to 0·17)	−49·4 (−62·7 to −32·9)
Armenia	101·4 (76·5 to 136·7)	51·3 (38·7 to 69·1)	−51·4 (−64·6 to −33·9)	0·30 (0·26 to 0·35)	0·09 (0·07 to 0·12)	−51·3 (−64·4 to −33·9)	436·7 (316·3 to 512·5)	14·5 (10·5 to 17·0)	−16·2 (−30·5 to −2·7)	0·97 (0·88 to 1·00)	0·16 (0·13 to 0·19)	−39·3 (−50·8 to −26·1)
Azerbaijan	1642·4 (1166·4 to 2242·2)	171·3 (121·7 to 233·9)	−48·9 (−64·2 to −27·7)	2·18 (1·88 to 2·49)	1·42 (1·01 to 1·93)	−48·8 (−64·2 to −27·6)	2641·8 (2187·1 to 3208·4)	27·0 (22·4 to 32·8)	−39·1 (−51·5 to −23·4)	4·63 (4·24 to 5·01)	1·74 (1·35 to 2·25)	−46·3 (−59·7 to −28·4)
Belarus	25·5 (17·6 to 37·5)	4·6 (3·2 to 6·8)	−65·7 (−76·0 to −51·0)	0·68 (0·58 to 0·78)	0·02 (0·02 to 0·03)	−64·4 (−74·9 to −50·0)	1246·5 (1020·5 to 1454·2)	13·0 (10·6 to 15·1)	−8·4 (−22·3 to 4·4)	3·09 (2·93 to 3·25)	0·35 (0·28 to 0·4)	−22·6 (−34·4 to −11·3)
Bosnia and Herzegovina	7·5 (5·1 to 10·8)	4·3 (2·9 to 6·3)	−65·7 (−76·0 to −51·0)	0·23 (0·20 to 0·27)	0·01 (0 to 0·01)	−53·5 (−68·7 to −28·4)	377·0 (278·5 to 590·6)	9·9 (7·3 to 15·5)	9·8 (−13·6 to 30·9)	0·93 (0·88 to 0·98)	0·06 (0·05 to 0·09)	−19·1 (−30·8 to −7·6)
Bulgaria	86·5 (58·6 to 128·1)	25·0 (16·9 to 37·0)	−49·5 (−65·6 to −27·7)	0·54 (0·47 to 0·61)	0·08 (0·05 to 0·11)	−49·2 (−65·3 to −27·7)	1794·5 (1628·2 to 1979·2)	24·7 (22·4 to 27·2)	−9·9 (−19·2 to 0·0)	2·20 (2·10 to 2·32)	0·37 (0·33 to 0·41)	−25·4 (−32·9 to −17·1)
Croatia	4·6 (3·6 to 5·9)	2·2 (1·7 to 2·8)	−55·5 (−65·4 to −43·4)	0·16 (0·14 to 0·18)	0 (0 to 0·01)	−53·9 (−63·6 to −42·2)	764·8 (681·5 to 866·4)	18·0 (16·1 to 20·4)	−29·6 (−37·7 to −19·6)	0·83 (0·80 to 0·87)	0·1 (0·09 to 0·11)	−39·3 (−44·7 to −32·6)
Czech Republic	13·1 (9·8 to 16·9)	2·4 (1·8 to 3·1)	−44·6 (−58·4 to −27·3)	0·45 (0·40 to 0·51)	0·01 (0·01 to 0·02)	−42·9 (−56·1 to −26·2)	3442·9 (3080·6 to 3841·0)	32·2 (28·8 to 35·9)	11·4 (−1·8 to 26·1)	2·56 (2·45 to 2·68)	0·48 (0·44 to 0·52)	−1·7 (−10·4 to 7·9)
Estonia	2·9 (2·0 to 4·0)	4·1 (2·8 to 5·6)	−67·5 (−77·4 to −54·4)	0·08 (0·07 to 0·09)	0 (0 to 0)	−66·4 (−76·2 to −53·6)	177·3 (154·8 to 203·2)	13·1 (11·4 to 15·0)	−26·1 (−36·3 to −13·8)	0·42 (0·40 to 0·44)	0·04 (0·03 to 0·05)	−42·0 (−51·0 to −31·5)
Georgia	73·2 (54·5 to 95·4)	26·2 (19·5 to 34·2)	−70·3 (−78·1 to −59·9)	0·33 (0·29 to 0·39)	0·06 (0·05 to 0·08)	−70·2 (−78·0 to −59·8)	650·7 (547·9 to 745·2)	16·2 (13·7 to 18·6)	−16·4 (−26·8 to −1·9)	1·12 (1·05 to 1·19)	0·18 (0·16 to 0·22)	−47·0 (−55·3 to −37·1)
Hungary	19·7 (12·8 to 27·8)	4·2 (2·8 to 6·0)	−45·5 (−62·6 to −23·9)	0·48 (0·42 to 0·56)	0·02 (0·01 to 0·02)	−44·3 (−60·7 to −23·2)	1025·0 (913·2 to 1155·5)	10·1 (9·0 to 11·4)	−4·3 (−16·4 to 9·8)	2·16 (2·04 to 2·27)	0·18 (0·16 to 0·2)	−19·5 (−28·1 to −9·3)
Kazakhstan	800·9 (617·7 to 1048·3)	43·1 (33·2 to 56·4)	−43·1 (−59·2 to −19·5)	2·43 (2·13 to 2·76)	0·69 (0·53 to 0·9)	−43·0 (−59·1 to −19·5)	3152·4 (2827·7 to 3519·7)	18·0 (16·1 to 20·1)	−22·9 (−33·4 to −10·6)	5·62 (5·26 to 6·02)	1·48 (1·3 to 1·71)	−32·0 (−43·9 to −17·8)
Kyrgyzstan	966·1 (807·0 to 1154·3)	131·6 (109·9 to 157·2)	−26·5 (−38·2 to −12·5)	1·35 (1·18 to 1·56)	0·83 (0·69 to 0·99)	−26·5 (−38·1 to −12·5)	1456·9 (1280·9 to 1680·2)	24·7 (21·7 to 28·5)	−21·5 (−30·7 to −11·1)	2·65 (2·44 to 2·88)	1·01 (0·87 to 1·17)	−24·2 (−34·6 to −12·3)
Latvia	5·2 (3·3 to 7·6)	5·1 (3·3 to 7·4)	−64·7 (−76·4 to −47·9)	0·13 (0·11 to 0·15)	0 (0 to 0·01)	−63·6 (−75·3 to −47·0)	368·2 (332·9 to 406·3)	16·6 (15·1 to 18·4)	−21·8 (−30·5 to −11·1)	0·70 (0·67 to 0·73)	0·09 (0·08 to 0·1)	−32·6 (−41·2 to −23·1)
Lithuania	8·3 (5·7 to 10·6)	5·4 (3·7 to 7·0)	−62·2 (−71·7 to −50·9)	0·19 (0·16 to 0·22)	0·01 (0·01 to 0·01)	−61·3 (−70·8 to −50·3)	583·5 (540·2 to 636·1)	18·5 (17·1 to 20·2)	−8·0 (−17·0 to 1·5)	1·04 (1·00 to 1·09)	0·14 (0·13 to 0·15)	−21·6 (−29·4 to −12·6)
Macedonia	15·6 (9·8 to 23·5)	13·6 (8·6 to 20·5)	−41·1 (−63·1 to −11·3)	0·17 (0·14 to 0·19)	0·01 (0·01 to 0·02)	−40·6 (−62·4 to −11·3)	146·8 (118·6 to 204·0)	7·1 (5·7 to 9·8)	−1·3 (−13·6 to 15·6)	0·49 (0·46 to 0·53)	0·04 (0·03 to 0·05)	−21·5 (−34·1 to −6·5)
Moldova	71·3 (45·5 to 104·2)	32·5 (20·7 to 47·5)	−47·7 (−67·6 to −18·5)	0·34 (0·29 to 0·39)	0·06 (0·04 to 0·09)	−47·6 (−67·3 to −18·6)	725·2 (653·5 to 809·6)	17·8 (16·1 to 19·9)	−26·0 (−35·5 to −13·4)	1·38 (1·30 to 1·46)	0·26 (0·23 to 0·3)	−33·9 (−44·3 to −21·0)
Mongolia	459·6 (330·1 to 638·3)	138·5 (99·5 to 192·3)	−44·5 (−59·1 to −22·5)	0·69 (0·60 to 0·80)	0·4 (0·28 to 0·55)	−44·5 (−59·0 to −22·5)	695·7 (546·6 to 914·1)	23·6 (18·5 to 31·0)	−35·4 (−48·4 to −18·1)	1·41 (1·29 to 1·53)	0·49 (0·37 to 0·65)	−40·4 (−53·8 to −21·7)
Montenegro	2·6 (1·6 to 4·0)	7·0 (4·2 to 10·8)	−69·5 (−81·4 to −50·1)	0·05 (0·04 to 0·06)	0 (0 to 0)	−68·8 (−80·6 to −49·7)	59·0 (48·5 to 72·2)	9·4 (7·7 to 11·5)	−4·9 (−20·9 to 14·4)	0·16 (0·15 to 0·17)	0·01 (0·01 to 0·01)	−32·2 (−41·8 to −20·0)
Poland	67·8 (47·3 to 94·5)	3·4 (2·4 to 4·7)	−47·1 (−63·5 to −26·2)	1·82 (1·59 to 2·09)	0·06 (0·04 to 0·08)	−46·0 (−61·8 to −25·6)	11 861·9 (10 678·7 to 13 177·8)	30·5 (27·4 to 33·9)	19·2 (5·3 to 34·2)	8·93 (8·50 to 9·36)	1·77 (1·64 to 1·91)	4·0 (−4·2 to 14·0)
Romania	410·3 (302·5 to 552·1)	43·7 (32·2 to 58·8)	−66·6 (−75·5 to −54·9)	1·80 (1·58 to 2·05)	0·36 (0·26 to 0·48)	−66·5 (−75·3 to −54·8)	5199·3 (4756·6 to 5665·5)	26·6 (24·4 to 29·0)	−19·4 (−26·2 to −11·6)	7·05 (6·70 to 7·41)	1·35 (1·23 to 1·5)	−41·4 (−47·1 to −34·6)
Russia	1483·6 (1287·2 to 1699·9)	16·6 (14·4 to 19·0)	−40·8 (−49·2 to −31·0)	9·89 (8·52 to 11·42)	1·29 (1·12 to 1·48)	−40·6 (−48·9 to −30·8)	40 832·4 (37 671·8 to 44 302·0)	27·6 (25·4 to 29·9)	−14·9 (−25·3 to −4·4)	49·78 (47·20 to 52·35)	12·9 (11·91 to 13·99)	−25·7 (−34·6 to −16·1)
Serbia	34·0 (26·2 to 43·7)	7·5 (5·8 to 9·6)	−41·4 (−55·7 to −21·1)	0·49 (0·43 to 0·56)	0·03 (0·02 to 0·04)	−41·0 (−55·1 to −21·1)	1143·1 (988·6 to 1379·0)	12·9 (11·2 to 15·6)	4·6 (−9·2 to 23·9)	1·89 (1·80 to 1·98)	0·21 (0·18 to 0·24)	−12·2 (−24·7 to 2·1)
Slovakia	27·9 (21·8 to 34·9)	9·8 (7·7 to 12·3)	−41·2 (−55·1 to −25·1)	0·35 (0·30 to 0·40)	0·02 (0·02 to 0·03)	−40·6 (−54·4 to −24·8)	1868·1 (1584·6 to 2132·2)	33·6 (28·5 to 38·4)	−6·6 (−17·1 to 5·0)	1·44 (1·37 to 1·51)	0·3 (0·26 to 0·34)	−18·1 (−24·9 to −9·9)
Slovenia	1·9 (1·3 to 2·5)	1·8 (1·2 to 2·3)	−44·5 (−58·2 to −25·0)	0·09 (0·08 to 0·11)	0 (0 to 0)	−42·0 (−55·3 to −23·2)	809·4 (700·3 to 925·4)	39·2 (33·9 to 44·8)	7·4 (−8·1 to 24·1)	0·50 (0·47 to 0·52)	0·08 (0·07 to 0·09)	−11·7 (−23·2 to 0·2)
Tajikistan	2022·9 (1420·9 to 2755·1)	169·3 (118·9 to 230·5)	−30·3 (−51·1 to −3·1)	2·34 (2·02 to 2·72)	1·74 (1·22 to 2·37)	−30·2 (−51·0 to −3·1)	3531·7 (2947·8 to 4263·7)	41·5 (34·7 to 50·2)	−21·0 (−35·3 to −2·4)	5·11 (4·66 to 5·60)	2·33 (1·82 to 2·96)	−26·2 (−42·5 to −4·3)
Turkmenistan	1411·6 (955·3 to 1968·8)	263·6 (178·4 to 367·6)	−50·0 (−66·6 to −26·0)	1·30 (1·11 to 1·50)	1·21 (0·82 to 1·69)	−50·0 (−66·6 to −26·0)	1938·4 (1451·6 to 2570·4)	36·0 (27·0 to 47·8)	−45·0 (−60·3 to −25·1)	2·80 (2·56 to 3·07)	1·45 (1·05 to 1·95)	−47·4 (−62·8 to −26·9)
Ukraine	193·3 (118·5 to 288·6)	8·0 (4·9 to 11·9)	−52·2 (−73·0 to −24·4)	2·49 (2·15 to 2·87)	0·17 (0·11 to 0·25)	−51·6 (−72·2 to −24·1)	6908·1 (6202·7 to 7813·3)	14·9 (13·3 to 16·8)	−22·2 (−32·4 to −8·8)	11·91 (11·32 to 12·52)	2·11 (1·88 to 2·39)	−33·6 (−42·9 to −20·7)
Uzbekistan	5890·9 (4407·1 to 7779·5)	181·7 (135·9 to 240·0)	−37·1 (−57·4 to −10·5)	7·42 (6·40 to 8·55)	5·07 (3·79 to 6·69)	−37·1 (−57·4 to −10·5)	9269·7 (7700·5 to 11 211·4)	31·0 (25·7 to 37·4)	−28·2 (−45·3 to −7·9)	16·37 (14·90 to 17·86)	6·41 (5·11 to 8·06)	−33·3 (−51·6 to −10·7)
**High income**	**1972·2 (1811·4 to 2162·6)**	**3·4 (3·1 to 3·7)**	**−34·9 (−40·4 to −28·6)**	**22·43 (19·85 to 25·32)**	**1·73 (1·6 to 1·9)**	**−34·4 (−39·8 to −28·2)**	**486 408·2 (470 465·3 to 501 521·2)**	**45·5 (44·0 to 46·9)**	**21·6 (18·4 to 24·7)**	**211·23 (203·63 to 218·10)**	**51·07 (49·67 to 52·43)**	**9·0 (6·2 to 11·9)**
Andorra	0·0 (0·0 to 0·0)	0·7 (0·4 to 1·2)	−50·1 (−68·1 to −23·7)	0 (0 to 0)	0 (0 to 0)	−47·8 (−65·2 to −22·8)	33·5 (23·6 to 46·5)	42·1 (29·8 to 58·6)	36·0 (−3·2 to 86·9)	0·01 (0·01 to 0·02)	0 (0 to 0)	21·2 (−4·6 to 50·9)
Argentina	584·8 (490·8 to 686·3)	15·7 (13·2 to 18·5)	−32·0 (−44·0 to −19·0)	3·17 (2·77 to 3·60)	0·51 (0·43 to 0·59)	−31·7 (−43·7 to −18·7)	31 200·9 (28 313·1 to 34 118·7)	71·9 (65·2 to 78·6)	25·4 (12·2 to 39·0)	13·35 (12·72 to 13·99)	4·59 (4·3 to 4·89)	9·7 (1·9 to 17·9)
Australia	34·1 (27·4 to 41·9)	2·2 (1·8 to 2·7)	−30·8 (−45·2 to −10·7)	0·44 (0·38 to 0·51)	0·03 (0·02 to 0·04)	−29·9 (−44·4 to −9·9)	4505·5 (3906·5 to 5209·4)	18·5 (16·1 to 21·4)	18·2 (−0·1 to 41·0)	2·87 (2·75 to 2·99)	0·45 (0·42 to 0·5)	5·7 (−4·5 to 18·3)
Austria	6·7 (5·3 to 8·4)	1·7 (1·3 to 2·1)	−35·4 (−48·9 to −18·1)	0·14 (0·12 to 0·16)	0·01 (0 to 0·01)	−34·4 (−47·4 to −17·6)	1316·7 (1161·9 to 1501·8)	15·2 (13·4 to 17·3)	−5·3 (−19·2 to 10·8)	1·17 (1·12 to 1·22)	0·13 (0·12 to 0·15)	−14·1 (−22·4 to −4·8)
Belgium	12·4 (10·1 to 15·1)	1·9 (1·6 to 2·3)	−24·0 (−39·9 to −4·5)	0·23 (0·20 to 0·27)	0·01 (0·01 to 0·01)	−23·5 (−38·7 to −4·7)	6397·3 (5615·6 to 7218·4)	56·4 (49·6 to 63·7)	12·0 (−2·6 to 27·4)	2·22 (2·12 to 2·32)	0·63 (0·57 to 0·69)	1·7 (−8·3 to 12·6)
Brunei	5·1 (4·1 to 6·2)	15·3 (12·2 to 18·6)	0·4 (−20·9 to 25·1)	0·02 (0·02 to 0·02)	0 (0 to 0·01)	0·5 (−20·7 to 25·0)	65·4 (56·7 to 84·6)	15·4 (13·4 to 20·0)	57·8 (42·0 to 77·3)	0·08 (0·08 to 0·08)	0·02 (0·01 to 0·02)	29·6 (16·7 to 43·3)
Canada	47·1 (38·1 to 57·0)	2·5 (2·0 to 3·0)	−13·6 (−30·5 to 6·3)	0·47 (0·41 to 0·55)	0·04 (0·03 to 0·05)	−12·9 (−29·6 to 6·9)	8742·5 (7676·1 to 9963·1)	24·2 (21·2 to 27·6)	22·8 (6·3 to 41·0)	4·36 (4·18 to 4·54)	0·94 (0·87 to 1·03)	12·8 (3·1 to 23·8)
Chile	94·5 (78·4 to 114·5)	8·1 (6·7 to 9·8)	−41·4 (−51·9 to −27·7)	0·89 (0·78 to 1·02)	0·08 (0·07 to 0·1)	−40·9 (−51·5 to −27·4)	6339·6 (5594·9 to 7094·5)	35·3 (31·2 to 39·5)	36·3 (18·2 to 55·6)	4·23 (4·03 to 4·41)	0·73 (0·68 to 0·8)	9·4 (−0·4 to 19·9)
Cyprus	1·5 (1·2 to 1·9)	4·1 (3·2 to 5·2)	−47·1 (−59·1 to −30·3)	0·01 (0·01 to 0·01)	0 (0 to 0)	−46·9 (−58·9 to −30·3)	147·9 (121·1 to 194·8)	16·6 (13·6 to 21·8)	−3·3 (−19·6 to 11·7)	0·09 (0·08 to 0·09)	0·02 (0·02 to 0·02)	−13·6 (−23·2 to −4·8)
Denmark	5·1 (4·0 to 6·6)	1·7 (1·3 to 2·2)	−35·5 (−50·8 to −17·8)	0·11 (0·09 to 0·13)	0 (0 to 0·01)	−34·7 (−49·6 to −17·8)	2380·2 (2086·8 to 2707·2)	41·7 (36·5 to 47·4)	7·4 (−7·2 to 23·1)	0·99 (0·95 to 1·04)	0·24 (0·21 to 0·26)	2·7 (−7·9 to 13·9)
Finland	1·9 (1·3 to 2·6)	0·7 (0·4 to 0·9)	−60·4 (−72·3 to −43·9)	0·09 (0·08 to 0·10)	0 (0 to 0)	−58·2 (−69·7 to −42·1)	916·2 (794·4 to 1055·5)	16·5 (14·3 to 19·0)	−27·6 (−37·7 to −15·4)	0·80 (0·76 to 0·84)	0·1 (0·09 to 0·1)	−34·7 (−41·2 to −27·2)
France	45·5 (33·2 to 60·0)	1·2 (0·8 to 1·5)	−31·9 (−51·0 to −6·7)	1·74 (1·51 to 2·02)	0·04 (0·03 to 0·06)	−29·2 (−47·7 to −4·8)	25 009·3 (21 466·4 to 29 059·2)	38·3 (32·9 to 44·5)	20·0 (2·9 to 41·8)	14·82 (14·11 to 15·55)	2·28 (2·05 to 2·51)	7·3 (−3·1 to 19·9)
Germany	42·6 (33·3 to 53·5)	1·3 (1·0 to 1·6)	−28·7 (−44·8 to −7·5)	1·10 (0·94 to 1·26)	0·04 (0·03 to 0·05)	−27·7 (−43·3 to −7·4)	31 582·8 (27 970·5 to 35 596·7)	37·8 (33·4 to 42·6)	20·8 (5·0 to 38·9)	15·33 (14·55 to 16·11)	3·41 (3·11 to 3·72)	9·8 (−0·4 to 21·0)
Greece	12·6 (9·6 to 20·2)	2·5 (1·9 to 4·0)	−52·2 (−63·2 to −38·5)	0·18 (0·16 to 0·21)	0·01 (0·01 to 0·02)	−51·5 (−62·3 to −38·1)	3153·0 (2741·3 to 3574·9)	28·9 (25·1 to 32·7)	4·8 (−10·1 to 20·6)	1·81 (1·73 to 1·90)	0·33 (0·3 to 0·36)	−12·4 (−21·2 to −3·0)
Greenland	0·8 (0·6 to 1·1)	10·8 (8·0 to 14·2)	−30·4 (−49·1 to −5·3)	0 (0 to 0)	0 (0 to 0)	−30·4 (−49·0 to −5·3)	8·9 (5·1 to 10·6)	16·4 (9·4 to 19·7)	−4·4 (−15·5 to 8·9)	0·01 (0·01 to 0·01)	0 (0 to 0)	−13·7 (−27·1 to 0·3)
Iceland	0·6 (0·5 to 0·8)	2·9 (2·3 to 3·8)	−47·9 (−58·6 to −33·7)	0·01 (0·01 to 0·01)	0 (0 to 0)	−47·2 (−57·5 to −33·5)	93·4 (81·0 to 107·1)	28·6 (24·8 to 32·8)	7·2 (−8·5 to 24·9)	0·05 (0·05 to 0·05)	0·01 (0·01 to 0·01)	−5·9 (−14·9 to 4·4)
Ireland	8·5 (6·9 to 10·5)	2·4 (1·9 to 2·9)	−36·9 (−49·9 to −20·2)	0·13 (0·11 to 0·15)	0·01 (0·01 to 0·01)	−36·1 (−48·8 to −19·9)	1653·2 (1435·4 to 1891·2)	34·5 (30·0 to 39·5)	−3·7 (−18·6 to 11·6)	0·73 (0·69 to 0·76)	0·17 (0·15 to 0·18)	−13·4 (−23·0 to −3·1)
Israel	18·3 (14·8 to 23·1)	2·2 (1·8 to 2·8)	−29·2 (−43·6 to −9·6)	0·29 (0·25 to 0·33)	0·02 (0·01 to 0·02)	−28·3 (−42·5 to −9·2)	2140·2 (1888·3 to 2413·1)	26·6 (23·5 to 30·0)	44·8 (27·0 to 65·5)	1·22 (1·16 to 1·28)	0·24 (0·22 to 0·26)	22·3 (12·4 to 34·4)
Italy	30·7 (22·9 to 39·1)	1·2 (0·9 to 1·5)	−42·5 (−57·7 to −23·5)	0·62 (0·53 to 0·71)	0·03 (0·02 to 0·03)	−41·5 (−56·2 to −23·0)	15 172·6 (13 132·5 to 17 635·5)	24·2 (20·9 to 28·1)	30·9 (11·0 to 54·8)	7·20 (6·86 to 7·55)	1·43 (1·3 to 1·59)	14·4 (2·4 to 28·0)
Japan	149·7 (127·1 to 169·7)	2·8 (2·4 to 3·2)	−38·2 (−44·8 to −30·5)	2·26 (1·98 to 2·57)	0·13 (0·11 to 0·15)	−37·6 (−44·2 to −30·2)	156 576·6 (150 156·7 to 162 966·9)	122·0 (117·0 to 127·0)	40·1 (34·3 to 46·0)	46·27 (44·37 to 48·08)	13·29 (12·77 to 13·86)	22·4 (17·0 to 28·1)
Luxembourg	0·7 (0·6 to 0·9)	2·5 (1·9 to 3·1)	−22·1 (−40·5 to 1·4)	0·01 (0·01 to 0·01)	0 (0 to 0)	−21·6 (−39·7 to 1·3)	131·6 (113·0 to 151·1)	23·6 (20·3 to 27·2)	12·9 (−4·4 to 33·6)	0·07 (0·07 to 0·08)	0·01 (0·01 to 0·01)	−0·8 (−12·0 to 11·1)
Malta	0·9 (0·8 to 1·1)	5·0 (4·1 to 6·1)	−30·8 (−45·3 to −11·9)	0·01 (0·01 to 0·01)	0 (0 to 0)	−30·5 (−44·9 to −11·8)	126·1 (110·7 to 141·5)	30·1 (26·5 to 33·8)	12·7 (−2·5 to 29·2)	0·06 (0·06 to 0·07)	0·01 (0·01 to 0·01)	−0·6 (−10·1 to 9·6)
Netherlands	17·3 (13·8 to 21·1)	2·0 (1·6 to 2·4)	−38·8 (−51·3 to −23·3)	0·45 (0·39 to 0·51)	0·02 (0·01 to 0·02)	−37·7 (−49·7 to −22·4)	7780·6 (6878·9 to 8714·0)	45·3 (40·0 to 50·7)	5·6 (−8·0 to 21·5)	3·71 (3·71 to 4·06)	0·78 (0·71 to 0·85)	−2·1 (−11·6 to 7·9)
New Zealand	12·4 (9·8 to 15·3)	4·1 (3·2 to 5·0)	−18·1 (−36·4 to 4·4)	0·12 (0·11 to 0·14)	0·01 (0·01 to 0·01)	−17·7 (−36·0 to 4·5)	729·3 (621·1 to 841·8)	16·0 (13·6 to 18·4)	24·5 (4·3 to 46·1)	0·64 (0·61 to 0·67)	0·07 (0·06 to 0·08)	8·3 (−4·0 to 21·3)
Norway	4·7 (3·4 to 5·9)	1·6 (1·1 to 2·0)	−33·1 (−49·2 to −13·2)	0·08 (0·07 to 0·09)	0 (0 to 0·01)	−32·4 (−47·9 to −12·9)	2195·3 (1910·1 to 2512·9)	42·5 (37·0 to 48·7)	−2·7 (−16·5 to 14·2)	0·64 (0·61 to 0·64)	0·18 (0·16 to 0·2)	−8·0 (−18·6 to 3·8)
Portugal	11·3 (9·1 to 13·8)	2·5 (2·1 to 3·1)	−63·8 (−71·5 to −55·7)	0·15 (0·13 to 0·17)	0·01 (0·01 to 0·01)	−63·3 (−70·9 to −55·2)	7053·4 (6269·6 to 7908·2)	65·3 (58·1 to 73·2)	15·6 (1·5 to 31·9)	2·12 (2·02 to 2·22)	0·74 (0·68 to 0·8)	−1·4 (−10·0 to 8·4)
Singapore	11·1 (8·3 to 13·4)	5·8 (4·3 to 7·0)	−32·5 (−43·6 to −18·1)	0·10 (0·09 to 0·11)	0·01 (0·01 to 0·01)	−32·2 (−43·2 to −18·1)	4253·2 (3899·8 to 4618·1)	108·4 (99·4 to 117·7)	63·1 (47·3 to 81·5)	1·19 (1·14 to 1·25)	0·44 (0·41 to 0·48)	32·3 (21·9 to 43·6)
South Korea	39·5 (30·9 to 50·5)	1·7 (1·4 to 2·2)	−53·3 (−63·7 to −38·9)	0·82 (0·72 to 0·95)	0·04 (0·03 to 0·04)	−52·4 (−62·8 to −38·4)	13 583·1 (11 974·6 to 15 291·9)	27·0 (23·8 to 30·4)	97·1 (71·1 to 125·5)	8·56 (8·19 to 8·93)	1·48 (1·34 to 1·62)	50·9 (35·7 to 67·2)
Spain	28·3 (22·9 to 35·2)	1·3 (1·1 to 1·6)	−48·8 (−59·3 to −34·8)	0·53 (0·46 to 0·60)	0·03 (0·02 to 0·03)	−47·9 (−58·4 to −34·2)	14 027·1 (12 316·2 to 15 910·7)	28·8 (25·3 to 32·6)	9·6 (−4·8 to 27·7)	5·98 (5·71 to 6·25)	1·37 (1·24 to 1·5)	−4·8 (−13·4 to 6·4)
Sweden	7·5 (4·5 to 9·7)	1·3 (0·8 to 1·7)	−23·0 (−38·8 to −2·4)	0·13 (0·11 to 0·15)	0·01 (0 to 0·01)	−22·1 (−37·6 to −1·7)	3290·2 (2905·5 to 3753·9)	33·5 (29·6 to 38·3)	−2·9 (−16·1 to 10·8)	1·13 (1·07 to 1·18)	0·3 (0·27 to 0·33)	−6·8 (−16·4 to 3·9)
Switzerland	9·0 (7·2 to 11·3)	2·2 (1·7 to 2·7)	−14·3 (−33·8 to 9·2)	0·14 (0·12 to 0·16)	0·01 (0·01 to 0·01)	−13·9 (−32·8 to 9·2)	1858·6 (1613·9 to 2138·5)	22·5 (19·5 to 25·8)	1·7 (−13·5 to 18·2)	1·08 (1·03 to 1·13)	0·17 (0·16 to 0·19)	−5·1 (−15·1 to 5·9)
UK	151·2 (129·9 to 168·5)	3·8 (3·2 to 4·2)	−19·0 (−27·9 to −9·0)	1·68 (1·47 to 1·91)	0·13 (0·11 to 0·15)	−18·7 (−27·3 to −9·0)	39 930·4 (37 967·4 to 41 942·9)	62·2 (59·1 to 65·3)	−3·4 (−8·2 to 1·3)	13·56 (13·03 to 14·09)	3·79 (3·61 to 3·96)	−10·5 (−14·5 to −6·4)
USA	538·5 (480·9 to 604·8)	2·7 (2·4 to 3·0)	−36·8 (−43·8 to −28·6)	6·03 (5·40 to 6·71)	0·47 (0·42 to 0·53)	−36·5 (−43·3 to −28·5)	91 996·2 (88 094·3 to 96 175·8)	28·4 (27·2 to 29·7)	8·0 (3·6 to 12·6)	53·35 (51·61 to 55·02)	12·47 (12·01 to 13·02)	5·1 (0·9 to 9·6)
Uruguay	36·4 (25·7 to 50·0)	15·0 (10·6 to 20·5)	−45·2 (−62·3 to −23·6)	0·37 (0·24 to 0·31)	0·03 (0·02 to 0·04)	−44·7 (−61·6 to −23·3)	1844·2 (1625·2 to 2075·8)	53·7 (47·3 to 60·4)	18·9 (3·0 to 36·9)	1·26 (1·20 to 1·31)	0·24 (0·22 to 0·26)	−5·0 (−14·3 to 4·6)
**Latin America and Caribbean**	**21 423·8 (19 529·9 to 23 622·2)**	**44·1 (40·2 to 48·6)**	**−45·3 (−49·9 to −40·6)**	**63·84 (56·83 to 71·72)**	**18·51 (16·87 to 20·37)**	**−45·2 (−49·7 to −40·5)**	**186 860·6 (160 496·9 to 199 589·1)**	**32·8 (28·2 to 35·0)**	**17·0 (11·2 to 23·5)**	**191·43 (182·52 to 200·70)**	**47·12 (42·5 to 49·71)**	**−18·9 (−23·0 to −14·8)**
Antigua and Barbuda	2·1 (1·4 to 3·0)	28·5 (19·2 to 41·0)	−24·8 (−44·7 to −0·9)	0·01 (0·01 to 0·02)	0 (0 to 0)	−24·5 (−44·2 to −0·7)	30·4 (26·5 to 34·5)	33·1 (28·8 to 37·5)	1·3 (−13·2 to 16·7)	0·94 (0·04 to 0·04)	0·01 (0·01 to 0·01)	−6·3 (−16·6 to 6·0)
Barbados	4·8 (2·8 to 8·1)	28·0 (16·4 to 47·7)	−29·3 (−61·8 to 30·1)	0·03 (0·02 to 0·03)	0 (0 to 0·01)	−29·0 (−61·3 to 29·8)	200·0 (173·4 to 228·7)	70·5 (61·1 to 80·6)	13·4 (−3·3 to 33·9)	0·15 (0·14 to 0·16)	0·03 (0·03 to 0·03)	3·6 (−10·0 to 19·6)
Belize	15·6 (9·9 to 24·9)	40·1 (25·4 to 64·0)	−19·3 (−52·8 to 38·3)	0·07 (0·06 to 0·08)	0·01 (0·01 to 0·02)	−19·1 (−52·4 to 38·0)	107·0 (92·4 to 123·0)	29·8 (25·8 to 34·3)	20·3 (4·3 to 38·8)	0·16 (0·15 to 0·18)	0·03 (0·03 to 0·04)	2·7 (−19·6 to 31·3)
Bermuda	0·2 (0·1 to 0·3)	5·5 (3·5 to 8·2)	−45·0 (−67·5 to −10·8)	0·01 (0·01 to 0·01)	0 (0 to 0)	−43·3 (−64·9 to −9·9)	16·1 (13·9 to 18·5)	24·1 (20·8 to 27·7)	15·6 (−1·3 to 35·1)	0·02 (0·02 to 0·03)	0 (0 to 0)	−2·4 (−15·0 to 12·4)
Bolivia	930·6 (630·8 to 1326·1)	75·8 (51·4 to 108·0)	−59·4 (−71·3 to −43·3)	2·79 (2·40 to 3·20)	0·81 (0·55 to 1·15)	−59·2 (−71·1 to −43·2)	7021·0 (5516·1 to 8656·8)	65·2 (51·2 to 80·4)	3·8 (−14·9 to 25·3)	7·40 (6·92 to 7·87)	1·81 (1·49 to 2·22)	−36·9 (−47·3 to −24·3)
Brazil	4677·3 (4125·4 to 5300·3)	31·1 (27·4 to 35·3)	−51·3 (−57·1 to −45·4)	23·31 (20·63 to 26·30)	4·06 (3·58 to 4·6)	−51·1 (−56·9 to −45·2)	75 602·0 (55 632·8 to 84 415·7)	36·4 (26·8 to 40·6)	31·6 (19·0 to 43·1)	89·83 (85·98 to 93·76)	16·45 (13·2 to 17·91)	−10·1 (−19·7 to −2·9)
Colombia	1234·9 (905·0 to 1683·7)	32·7 (24·0 to 44·6)	−43·6 (−59·0 to −22·7)	4·57 (4·02 to 5·17)	1·07 (0·79 to 1·46)	−43·4 (−58·7 to −22·6)	8230·5 (7531·4 to 9056·1)	17·1 (15·6 to 18·8)	3·3 (−7·0 to 14·9)	10·60 (9·95 to 11·29)	2·33 (2·04 to 2·71)	−24·2 (−35·1 to −10·3)
Costa Rica	36·5 (24·2 to 53·8)	10·4 (6·9 to 15·3)	−46·0 (−62·4 to −23·2)	0·43 (0·38 to 0·49)	0·03 (0·02 to 0·05)	−45·2 (−61·5 to −22·9)	607·1 (541·7 to 681·4)	12·6 (11·3 to 14·2)	21·5 (6·9 to 36·8)	1·06 (0·99 to 1·13)	0·12 (0·1 to 0·13)	−9·8 (−21·3 to 2·6)
Cuba	66·8 (55·1 to 78·5)	11·3 (9·3 to 13·3)	−33·3 (−44·5 to −20·6)	0·75 (0·65 to 0·86)	0·06 (0·05 to 0·07)	−32·8 (−43·9 to −20·4)	7016·0 (6239·0 to 7855·3)	61·6 (54·8 to 69·0)	13·3 (−0·2 to 27·8)	4·72 (4·51 to 4·93)	0·83 (0·77 to 0·91)	0·5 (−8·3 to 10·0)
Dominica	3·0 (1·8 to 4·8)	53·8 (32·5 to 85·8)	2·5 (−33·1 to 55·2)	0·01 (0·01 to 0·01)	0 (0 to 0)	2·5 (−32·9 to 54·8)	28·4 (23·4 to 34·6)	39·6 (32·7 to 48·3)	21·9 (−1·4 to 43·9)	0·03 (0·03 to 0·04)	0·01 (0·01 to 0·01)	11·4 (−8·0 to 35·6)
Dominican Republic	536·6 (404·1 to 708·3)	50·5 (38·0 to 66·6)	−37·8 (−54·4 to −13·0)	2·04 (1·78 to 2·33)	0·46 (0·35 to 0·61)	−37·6 (−54·1 to −12·9)	2532·7 (2090·6 to 2928·0)	24·1 (19·9 to 27·8)	12·3 (−2·9 to 30·9)	4·71 (4·40 to 5·05)	0·81 (0·67 to 0·96)	−21·1 (−35·0 to −1·8)
Ecuador	1099·1 (892·2 to 1360·0)	67·8 (55·1 to 83·9)	−43·6 (−54·0 to −30·9)	1·75 (1·54 to 1·96)	0·94 (0·77 to 1·17)	−43·6 (−54·0 to −30·9)	6569·5 (5763·2 to 7768·5)	40·7 (35·7 to 48·1)	5·2 (−7·2 to 17·2)	4·75 (4·51 to 5·00)	1·84 (1·63 to 2·14)	−25·3 (−34·5 to −15·4)
El Salvador	156·2 (104·3 to 223·9)	29·4 (19·7 to 42·2)	−60·0 (−74·1 to −41·0)	0·64 (0·56 to 0·74)	0·14 (0·09 to 0·19)	−59·8 (−73·9 to −40·8)	2474·8 (1797·9 to 2895·6)	40·3 (29·3 to 47·2)	6·4 (−8·2 to 20·0)	1·71 (1·61 to 1·82)	0·54 (0·44 to 0·63)	−27·4 (−38·2 to −15·1)
Grenada	4·6 (2·5 to 7·9)	45·5 (24·7 to 77·9)	−7·6 (−50·6 to 74·5)	0·02 (0·02 to 0·02)	0 (0 to 0·01)	−7·4 (−50·1 to 74·2)	63·5 (55·3 to 74·7)	59·4 (51·7 to 69·9)	−1·4 (−15·4 to 12·7)	0·06 (0·05 to 0·06)	0·01 (0·01 to 0·02)	−3·1 (−19·2 to 17·9)
Guatemala	2994·0 (2348·2 to 3765·9)	142·5 (111·7 to 179·2)	−46·8 (−56·2 to −35·0)	3·89 (3·41 to 4·40)	2·57 (2·02 to 3·23)	−46·7 (−56·1 to −35·0)	9940·8 (8746·0 to 11 163·9)	60·8 (53·5 to 68·3)	−17·1 (−26·8 to −8·0)	6·61 (6·10 to 7·13)	4·1 (3·48 to 4·79)	−37·3 (−45·5 to −28·2)
Guyana	33·6 (24·0 to 47·0)	48·0 (34·3 to 67·0)	−44·6 (−60·0 to −24·1)	0·12 (0·10 to 0·14)	0·03 (0·02 to 0·04)	−44·5 (−59·8 to −23·9)	248·5 (209·7 to 285·7)	32·3 (27·2 to 37·1)	−14·3 (−25·9 to −2·9)	0·31 (0·29 to 0·33)	0·08 (0·07 to 0·1)	−24·6 (−35·7 to −12·1)
Haiti	2384·2 (1485·2 to 3643·7)	191·1 (119·0 to 292·0)	−39·7 (−63·6 to −3·5)	3·28 (2·81 to 3·75)	2·05 (1·28 to 3·13)	−39·6 (−63·4 to −3·5)	6015·6 (4657·6 to 7710·6)	56·1 (43·4 to 71·9)	−12·1 (−31·5 to 12·0)	6·28 (5·79 to 6·79)	2·93 (2·09 to 4·03)	−29·5 (−51·1 to 0·1)
Honduras	338·5 (250·7 to 446·8)	40·4 (29·9 to 53·4)	−41·3 (−58·4 to −19·8)	1·28 (1·10 to 1·46)	0·29 (0·22 to 0·39)	−41·1 (−58·2 to −19·8)	1415·1 (1083·0 to 1812·8)	17·5 (13·4 to 22·4)	0·6 (−20·1 to 22·2)	2·32 (2·14 to 2·52)	0·49 (0·4 to 0·61)	−27·5 (−43·4 to −9·6)
Jamaica	56·2 (37·1 to 85·9)	23·4 (15·5 to 35·8)	−21·5 (−55·2 to 36·1)	0·39 (0·34 to 0·46)	0·05 (0·03 to 0·07)	−21·2 (−54·7 to 35·6)	787·9 (652·9 to 992·4)	27·8 (23·1 to 35·1)	15·2 (−6·1 to 36·7)	1·14 (1·07 to 1·21)	0·16 (0·13 to 0·19)	−2·6 (−23·0 to 20·4)
Mexico	3343·2 (2965·9 to 3798·7)	28·7 (25·5 to 32·6)	−44·9 (−51·3 to −37·3)	5·20 (4·56 to 5·93)	2·88 (2·56 to 3·28)	−44·9 (−51·2 to −37·2)	24 848·1 (23 587·0 to 26 016·5)	19·6 (18·6 to 20·5)	21·0 (14·1 to 27·5)	13·38 (12·65 to 14·16)	6·77 (6·39 to 7·2)	−17·1 (−22·3 to −11·0)
Nicaragua	330·2 (252·9 to 423·8)	53·9 (41·3 to 69·2)	−47·1 (−60·0 to −31·2)	0·96 (0·84 to 1·10)	0·29 (0·22 to 0·37)	−47·0 (−59·9 to −31·1)	1059·8 (893·8 to 1277·2)	17·4 (14·7 to 21·0)	−7·8 (−21·6 to 7·6)	1·82 (1·68 to 1·96)	0·43 (0·36 to 0·52)	−35·2 (−46·3 to −21·2)
Panama	165·1 (120·9 to 229·1)	44·9 (32·9 to 62·3)	−6·4 (−30·3 to 27·6)	0·52 (0·45 to 0·59)	0·14 (0·1 to 0·2)	−6·3 (−30·0 to 27·6)	1046·6 (847·6 to 1235·6)	26·6 (21·6 to 31·5)	42·6 (20·9 to 69·7)	1·10 (1·02 to 1·17)	0·28 (0·24 to 0·34)	12·2 (−8·1 to 36·6)
Paraguay	222·2 (158·2 to 295·5)	32·3 (23·0 to 43·0)	−43·6 (−60·5 to −22·4)	2·30 (2·00 to 2·66)	0·2 (0·14 to 0·26)	−43·0 (−59·7 to −22·0)	1724·9 (1410·1 to 2078·0)	25·9 (21·2 to 31·2)	11·3 (−10·2 to 34·2)	5·45 (5·10 to 5·84)	0·48 (0·4 to 0·57)	−18·6 (−33·2 to −2·3)
Peru	1537·5 (1268·4 to 1858·1)	50·6 (41·8 to 61·2)	−44·4 (−54·9 to −31·3)	5·48 (4·86 to 6·17)	1·33 (1·1 to 1·6)	−44·3 (−54·7 to −31·2)	19 313·5 (15 391·4 to 22 215·9)	61·5 (49·0 to 70·8)	19·3 (4·4 to 36·7)	17·53 (16·62 to 18·45)	4·05 (3·34 to 4·55)	−16·3 (−26·3 to −4·9)
Puerto Rico	16·5 (13·1 to 20·6)	7·4 (5·9 to 9·3)	−50·4 (−61·1 to −36·3)	0·26 (0·23 to 0·30)	0·01 (0·01 to 0·02)	−49·8 (−60·1 to −35·7)	2036·2 (1806·3 to 2308·0)	55·3 (49·0 to 62·7)	0·4 (−11·0 to 14·2)	1·60 (1·53 to 1·68)	0·26 (0·24 to 0·29)	−11·1 (−18·9 to −2·3)
Saint Lucia	5·0 (2·7 to 9·3)	36·4 (19·3 to 66·7)	−32·4 (−66·3 to 39·0)	0·03 (0·02 to 0·03)	0 (0 to 0·01)	−32·1 (−65·9 to 39·0)	79·1 (69·0 to 90·4)	42·8 (37·3 to 48·9)	12·9 (−4·1 to 32·8)	0·08 (0·08 to 0·09)	0·02 (0·01 to 0·02)	−4·6 (−24·7 to 24·9)
Saint Vincent and the Grenadines	3·3 (2·1 to 5·4)	37·8 (23·5 to 61·4)	−37·9 (−60·5 to 1·0)	0·02 (0·01 to 0·02)	0 (0 to 0)	−37·6 (−60·1 to 1·1)	41·1 (37·0 to 46·0)	37·5 (33·7 to 41·9)	−2·0 (−13·5 to 11·9)	0·05 (0·05 to 0·05)	0·01 (0·01 to 0·01)	−14·1 (−27·9 to 3·1)
Suriname	26·3 (18·8 to 36·0)	55·1 (39·4 to 75·4)	−39·4 (−53·4 to −21·9)	0·08 (0·07 to 0·10)	0·02 (0·02 to 0·03)	−39·2 (−53·1 to −21·9)	175·0 (148·4 to 203·5)	32·2 (27·3 to 37·5)	5·8 (−7·6 to 21·7)	0·23 (0·22 to 0·25)	0·05 (0·04 to 0·06)	−20·0 (−31·2 to −7·0)
The Bahamas	8·5 (4·9 to 15·6)	30·0 (17·1 to 55·1)	−11·6 (−50·2 to 58·8)	0·05 (0·05 to 0·06)	0·01 (0 to 0·01)	−11·2 (−49·7 to 58·7)	135·1 (115·5 to 164·3)	34·9 (29·8 to 42·4)	33·8 (17·4 to 53·3)	0·17 (0·16 to 0·18)	0·03 (0·03 to 0·04)	10·9 (−6·7 to 32·0)
Trinidad and Tobago	23·4 (14·8 to 38·1)	24·2 (15·3 to 39·4)	−32·2 (−54·4 to 4·1)	0·13 (0·11 to 0·16)	0·02 (0·01 to 0·03)	−31·9 (−54·1 to 4·2)	359·1 (313·8 to 407·8)	26·4 (23·1 to 30·0)	9·9 (−3·9 to 25·3)	0·49 (0·46 to 0·52)	0·08 (0·07 to 0·1)	−7·0 (−19·4 to 8·0)
Venezuela	1066·1 (916·0 to 1261·6)	36·0 (31·0 to 42·7)	−2·9 (−19·0 to 16·6)	3·18 (2·76 to 3·70)	0·92 (0·79 to 1·09)	−2·7 (−18·7 to 16·7)	6093·1 (5444·4 to 6808·8)	19·6 (17·5 to 21·9)	31·4 (15·5 to 49·0)	6·73 (6·27 to 7·27)	1·84 (1·66 to 2·04)	11·2 (−1·1 to 24·1)
Virgin Islands	0·6 (0·5 to 0·8)	8·3 (6·4 to 11·0)	−37·2 (−52·0 to −17·9)	0·01 (0·01 to 0·01)	0 (0 to 0)	−36·4 (−51·0 to −17·5)	38·7 (32·6 to 48·0)	36·3 (30·6 to 45·0)	40·5 (18·5 to 62·7)	0·05 (0·05 to 0·05)	0·01 (0·01 to 0·01)	16·8 (1·6 to 31·8)
**North Africa and Middle East**	**49 979·5 (41 384·5 to 60 086·1)**	**77·7 (64·4 to 93·4)**	**−38·1 (−47·7 to −25·9)**	**115·37 (100·82 to 131·39)**	**43·06 (35·65 to 51·7)**	**−37·9 (−47·5 to −25·8)**	**126 059·4 (113 173·2 to 139 514·6)**	**22·3 (20·0 to 24·6)**	**−10·3 (−18·4 to −1·4)**	**274·75 (255·92 to 294·48)**	**62·12 (53·56 to 71·27)**	**−27·7 (−36·8 to −17·4)**
Afghanistan	19 116·3 (12 797·7 to 26 098·6)	380·0 (254·4 to 518·7)	−37·7 (−55·4 to −16·2)	9·27 (7·96 to 10·82)	16·4 (10·99 to 22·35)	−37·6 (−55·3 to −16·1)	25 847·5 (18 986·5 to 33 169·1)	79·3 (58·2 to 101·7)	−28·9 (−44·7 to −9·3)	18·32 (16·59 to 20·18)	18·98 (13·31 to 25·02)	−33·6 (−50·5 to −12·8)
Algeria	949·4 (602·9 to 1374·3)	20·8 (13·2 to 30·2)	−19·5 (−49·2 to 25·0)	7·24 (6·16 to 8·49)	0·83 (0·53 to 1·19)	−19·0 (−48·7 to 25·3)	7772·3 (6488·4 to 9162·8)	19·6 (16·4 to 23·1)	32·5 (11·4 to 58·1)	18·36 (16·98 to 19·80)	2·22 (1·83 to 2·64)	2·1 (−15·6 to 24·3)
Bahrain	8·5 (5·9 to 11·1)	8·6 (6·0 to 11·2)	−29·6 (−47·0 to −5·2)	0·13 (0·11 to 0·15)	0·01 (0·01 to 0·01)	−28·7 (−46·0 to −4·4)	86·1 (69·6 to 101·8)	6·3 (5·1 to 7·4)	21·1 (1·1 to 43·8)	0·41 (0·39 to 0·44)	0·03 (0·02 to 0·03)	4·3 (−12·1 to 23·6)
Egypt	9759·7 (7022·8 to 13 347·1)	83·0 (59·7 to 113·5)	−32·7 (−53·3 to −4·4)	18·84 (16·53 to 21·22)	8·43 (6·07 to 11·51)	−32·6 (−53·1 to −4·3)	23 381·7 (19 935·1 to 28 468·5)	25·7 (21·9 to 31·2)	−6·1 (−20·3 to 11·7)	41·72 (38·99 to 44·81)	11·74 (9·36 to 14·81)	−23·0 (−40·8 to 0·4)
Iran	1161·4 (754·2 to 1737·4)	17·1 (11·1 to 25·6)	−54·1 (−72·9 to −26·5)	10·14 (8·79 to 11·70)	1·02 (0·67 to 1·51)	−53·6 (−72·1 to −26·3)	9733·7 (7234·5 to 12 030·6)	12·3 (9·2 to 15·2)	9·4 (−19·1 to 42·5)	30·39 (28·53 to 32·40)	2·82 (2·25 to 3·41)	−23·3 (−44·2 to 1·7)
Iraq	2696·3 (1872·4 to 3648·5)	47·1 (32·7 to 63·7)	−33·5 (−52·0 to −9·7)	10·35 (8·88 to 11·99)	2·34 (1·63 to 3·16)	−33·2 (−51·6 to −9·5)	5965·8 (4832·5 to 7134·3)	16·4 (13·3 to 19·6)	−14·1 (−30·8 to 3·3)	20·64 (18·85 to 22·61)	3·38 (2·59 to 4·2)	−23·7 (−40·6 to −3·8)
Jordan	269·7 (208·8 to 348·3)	28·2 (21·8 to 36·4)	−13·6 (−35·2 to 14·9)	1·53 (1·32 to 1·75)	0·23 (0·18 to 0·3)	−13·3 (−34·8 to 15·0)	807·3 (687·8 to 933·9)	10·7 (9·1 to 12·3)	9·3 (−7·8 to 30·6)	3·28 (3·03 to 3·54)	0·36 (0·31 to 0·43)	−4·5 (−21·5 to 16·8)
Kuwait	36·2 (27·3 to 48·7)	10·1 (7·6 to 13·7)	6·0 (−23·0 to 44·5)	0·45 (0·40 to 0·52)	0·03 (0·02 to 0·04)	6·8 (−21·6 to 44·8)	305·3 (268·0 to 350·0)	7·8 (6·9 to 9·0)	56·5 (35·4 to 79·9)	1·28 (1·21 to 1·36)	0·09 (0·08 to 0·11)	34·3 (15·5 to 57·9)
Lebanon	25·3 (14·4 to 41·4)	6·8 (3·9 to 11·1)	−44·4 (−67·4 to −4·0)	0·67 (0·58 to 0·78)	0·02 (0·01 to 0·04)	−42·8 (−65·4 to −2·9)	334·8 (243·4 to 439·5)	5·8 (4·2 to 7·6)	38·6 (6·1 to 78·1)	2·27 (2·14 to 2·41)	0·08 (0·06 to 0·09)	−5·1 (−26·2 to 24·7)
Libya	100·8 (66·6 to 146·6)	15·1 (10·0 to 22·0)	−43·6 (−61·9 to −14·9)	1·00 (0·85 to 1·19)	0·09 (0·06 to 0·13)	−43·2 (−61·3 to −14·9)	1006·3 (785·4 to 1242·5)	16·0 (12·5 to 19·7)	15·2 (−8·7 to 42·0)	2·81 (2·60 to 3·04)	0·29 (0·23 to 0·34)	−12·1 (−28·7 to 6·5)
Morocco	991·7 (676·8 to 1417·2)	29·0 (19·8 to 41·5)	−47·8 (−64·1 to −25·8)	5·92 (5·09 to 6·82)	0·86 (0·59 to 1·22)	−47·6 (−63·8 to −25·9)	8631·4 (6650·8 to 11 279·2)	25·1 (19·3 to 32·8)	10·8 (−12·9 to 37·4)	17·26 (16·22 to 18·29)	2·55 (1·99 to 3·2)	−17·8 (−35·1 to 2·3)
Oman	35·9 (26·0 to 49·4)	9·6 (6·9 to 13·1)	10·5 (−25·7 to 59·7)	0·43 (0·36 to 0·50)	0·03 (0·02 to 0·04)	11·1 (−24·6 to 59·4)	663·8 (493·3 to 805·0)	14·8 (11·0 to 18·0)	67·5 (36·3 to 102·6)	1·45 (1·35 to 1·55)	0·17 (0·14 to 0·21)	55·3 (29·2 to 85·7)
Palestine	100·7 (67·9 to 144·1)	14·2 (9·6 to 20·4)	−33·7 (−55·7 to −1·5)	1·07 (0·92 to 1·25)	0·09 (0·06 to 0·13)	−33·1 (−55·0 to −1·3)	659·8 (526·9 to 844·0)	14·1 (11·3 to 18·1)	17·4 (−8·9 to 45·1)	2·54 (2·31 to 2·77)	0·28 (0·23 to 0·35)	−2·2 (−22·5 to 20·2)
Qatar	4·6 (2·8 to 7·4)	3·9 (2·3 to 6·3)	5·6 (−38·9 to 80·7)	0·16 (0·14 to 0·19)	0 (0 to 0·01)	9·1 (−34·3 to 81·7)	59·4 (42·2 to 76·7)	2·7 (1·9 to 3·5)	76·5 (32·1 to 134·8)	0·52 (0·49 to 0·55)	0·02 (0·02 to 0·03)	67·7 (27·3 to 118·5)
Saudi Arabia	132·0 (106·6 to 161·3)	4·3 (3·5 to 5·3)	−43·1 (−54·3 to −30·6)	2·40 (2·08 to 2·75)	0·12 (0·09 to 0·14)	−42·2 (−53·3 to −30·0)	4065·6 (2971·9 to 4697·9)	12·9 (9·5 to 14·9)	22·6 (9·2 to 38·2)	9·81 (9·26 to 10·34)	0·96 (0·78 to 1·08)	7·7 (−6·3 to 21·0)
Sudan	8684·1 (5480·7 to 13 365·3)	142·2 (89·8 to 218·9)	−35·9 (−58·3 to 0·8)	15·41 (13·00 to 18·00)	7·45 (4·72 to 11·43)	−35·8 (−58·1 to 0·6)	15 005·5 (10 928·5 to 20 078·3)	37·2 (27·1 to 49·7)	−20·1 (−41·1 to 8·3)	28·95 (26·10 to 31·82)	9·26 (6·53 to 13·26)	−29·9 (−50·6 to 3·1)
Syria	750·5 (527·2 to 980·0)	32·5 (22·8 to 42·4)	0·1 (−35·6 to 83·3)	3·94 (3·38 to 4·62)	0·65 (0·46 to 0·84)	−0·2 (−35·3 to 80·6)	2670·8 (2041·5 to 4203·1)	14·3 (11·0 to 22·6)	15·1 (−5·7 to 46·1)	9·64 (8·81 to 10·59)	1·19 (0·96 to 1·49)	3·9 (−20·1 to 51·6)
Tunisia	110·7 (79·4 to 149·0)	11·3 (8·1 to 15·2)	−37·6 (−56·0 to −10·5)	0·95 (0·81 to 1·12)	0·1 (0·07 to 0·13)	−37·2 (−55·5 to −10·5)	2485·2 (1907·5 to 3242·5)	22·1 (17·0 to 28·8)	18·1 (−4·2 to 41·8)	3·58 (3·37 to 3·78)	0·54 (0·44 to 0·66)	−4·6 (−19·3 to 11·4)
Turkey	984·5 (624·4 to 1512·8)	15·4 (9·8 to 23·7)	−76·4 (−84·7 to −64·5)	11·69 (10·11 to 13·52)	0·87 (0·56 to 1·32)	−76·0 (−84·3 to −64·1)	7991·6 (6759·6 to 9798·4)	10·2 (8·6 to 12·5)	−21·8 (−35·3 to −3·4)	35·26 (33·04 to 37·54)	2·24 (1·91 to 2·71)	−56·3 (−65·3 to −45·4)
United Arab Emirates	10·2 (5·1 to 18·8)	2·1 (1·1 to 3·9)	−24·4 (−62·7 to 54·2)	0·41 (0·35 to 0·49)	0·01 (0·01 to 0·02)	−21·2 (−58·9 to 54·7)	471·0 (328·4 to 620·1)	5·1 (3·6 to 6·8)	98·7 (52·3 to 154·2)	1·63 (1·52 to 1·74)	0·15 (0·11 to 0·2)	88·2 (41·8 to 140·5)
Yemen	4012·9 (2765·2 to 5560·7)	100·2 (69·1 to 138·9)	−34·4 (−58·7 to 51·0)	13·28 (11·52 to 15·09)	3·47 (2·41 to 4·8)	−34·1 (−58·4 to 50·1)	8019·2 (5851·7 to 10 684·7)	29·8 (21·7 to 39·7)	−15·9 (−40·1 to 43·4)	24·40 (22·39 to 26·60)	4·72 (3·45 to 6·13)	−25·3 (−49·5 to 50·4)
**South Asia**	**205 488·6 (183 136·1 to 230 519·5)**	**122·9 (109·5 to 137·8)**	**−45·0 (−51·5 to −37·4)**	**384·32 (341·28 to 429·39)**	**177 (157·8 to 198·39)**	**−44·9 (−51·4 to −37·4)**	**642 560·9 (568 623·7 to 695 400·7)**	**38·0 (33·6 to 41·1)**	**−14·7 (−20·4 to −8·4)**	**1027·10 (975·23 to 1080·80)**	**296·51 (267·39 to 322·51)**	**−31·9 (−37·8 to −25·4)**
Bangladesh	21 274·9 (17 071·7 to 25 804·0)	139·1 (111·6 to 168·7)	−56·6 (−65·3 to −47·3)	27·22 (23·80 to 31·09)	18·36 (14·74 to 22·25)	−56·5 (−65·2 to −47·3)	38 666·3 (32 506·1 to 47 679·7)	24·0 (20·2 to 29·6)	−43·5 (−52·8 to −32·8)	69·63 (65·38 to 74·29)	22·89 (19·23 to 26·76)	−52·5 (−60·8 to −44·0)
Bhutan	43·6 (27·1 to 63·9)	66·0 (40·9 to 96·6)	−56·0 (−70·2 to −36·4)	0·13 (0·12 to 0·16)	0·04 (0·02 to 0·06)	−55·8 (−70·1 to −36·3)	200·8 (146·4 to 264·6)	25·9 (18·9 to 34·1)	−7·0 (−26·2 to 16·2)	0·39 (0·37 to 0·42)	0·08 (0·06 to 0·1)	−35·6 (−50·4 to −18·2)
India	140 649·3 (122 929·9 to 160 757·9)	113·2 (99·0 to 129·4)	−46·5 (−53·8 to −37·9)	297·56 (264·96 to 332·97)	121·15 (105·92 to 138·3)	−46·3 (−53·6 to −37·8)	529 381·1 (456 398·6 to 578 182·8)	40·4 (34·8 to 44·1)	−11·3 (−17·5 to −3·9)	834·91 (794·19 to 877·12)	226·35 (198·54 to 248·35)	−30·4 (−37·0 to −23·2)
Nepal	4362·7 (3486·1 to 5342·2)	153·4 (122·6 to 187·8)	−62·9 (−70·4 to −53·7)	5·49 (4·73 to 6·32)	3·76 (3·01 to 4·6)	−62·9 (−70·3 to −53·6)	11 088·1 (8134·1 to 14 310·1)	38·8 (28·5 to 50·1)	−35·5 (−48·2 to −23·0)	14·67 (13·69 to 15·68)	5·55 (4·5 to 6·72)	−52·7 (−61·2 to −43·5)
Pakistan	39 158·0 (29 521·5 to 49 842·8)	157·7 (118·9 to 200·8)	−22·0 (−42·4 to 5·0)	53·90 (46·94 to 61·43)	33·7 (25·39 to 42·88)	−21·9 (−42·2 to 5·0)	63 224·6 (51 828·1 to 74 822·4)	33·4 (27·4 to 39·6)	−10·6 (−27·5 to 10·4)	107·49 (99·53 to 115·86)	41·65 (33·11 to 50·89)	−17·0 (−34·9 to 7·1)
**Southeast Asia, east Asia, and Oceania**	**68 893·1 (61 004·9 to 77 022·9)**	**46·9 (41·6 to 52·5)**	**−56·3 (−61·7 to −49·9)**	**118·59 (103·43 to 135·04)**	**59·38 (52·62 to 66·35)**	**−56·2 (−61·6 to −49·9)**	**459 114·4 (407 453·4 to 494 274·7)**	**21·9 (19·4 to 23·6)**	**−5·2 (−10·9 to 0·2)**	**454·59 (433·76 to 475·58)**	**127·36 (116·96 to 137·32)**	**−36·6 (−41·5 to −31·4)**
American Samoa	1·5 (1·1 to 2·1)	13·3 (9·6 to 17·9)	−39·4 (−55·6 to −18·8)	0·02 (0·02 to 0·02)	0 (0 to 0)	−38·5 (−54·5 to −18·0)	14·6 (12·3 to 17·8)	17·7 (14·8 to 21·5)	3·1 (−14·1 to 23·4)	0·06 (0·05 to 0·06)	0 (0 to 0·01)	−7·6 (−22·7 to 9·8)
Cambodia	2135·9 (1578·4 to 2724·7)	119·6 (88·4 to 152·5)	−63·5 (−73·0 to −51·2)	2·79 (2·39 to 3·21)	1·84 (1·36 to 2·35)	−63·4 (−72·9 to −51·1)	6440·4 (5190·7 to 7598·9)	41·3 (33·3 to 48·7)	−33·3 (−45·2 to −20·0)	7·17 (6·72 to 7·67)	2·88 (2·34 to 3·45)	−52·7 (−61·8 to −41·0)
China	24 247·0 (21 333·8 to 28 877·5)	29·2 (25·7 to 34·7)	−61·2 (−66·1 to −54·4)	36·98 (32·43 to 42·14)	20·92 (18·41 to 24·89)	−61·2 (−66·1 to −54·3)	205 088·4 (182 533·2 to 234 201·2)	14·8 (13·2 to 16·9)	−9·7 (−16·2 to −2·7)	187·22 (179·60 to 194·51)	48·06 (44·06 to 54·54)	−42·3 (−47·0 to −37·2)
Federated States of Micronesia	3·5 (2·0 to 5·6)	29·0 (16·6 to 45·9)	−48·6 (−69·9 to −14·1)	0·03 (0·02 to 0·03)	0 (0 to 0)	−48·3 (−69·4 to −14·1)	34·0 (23·1 to 51·4)	32·4 (22·0 to 49·0)	−14·5 (−34·8 to 11·0)	0·08 (0·08 to 0·09)	0·01 (0·01 to 0·02)	−23·7 (−42·7 to −0·0)
Fiji	94·5 (49·5 to 167·4)	107·5 (56·3 to 190·6)	−24·5 (−60·1 to 33·5)	0·21 (0·17 to 0·25)	0·08 (0·04 to 0·14)	−24·4 (−59·9 to 33·4)	417·3 (338·9 to 544·0)	46·8 (38·0 to 61·0)	0·6 (−21·6 to 28·5)	0·67 (0·63 to 0·72)	0·17 (0·13 to 0·24)	−13·5 (−38·9 to 19·8)
Guam	5·4 (4·0 to 7·3)	38·4 (28·3 to 51·6)	4·3 (−25·7 to 46·2)	0·03 (0·03 to 0·03)	0 (0 to 0·01)	4·2 (−25·4 to 45·5)	58·3 (48·3 to 72·8)	34·3 (28·4 to 42·8)	39·2 (12·4 to 66·2)	0·12 (0·11 to 0·12)	0·02 (0·01 to 0·02)	20·2 (−0·5 to 44·7)
Indonesia	15 250·3 (9900·3 to 20 124·9)	61·5 (39·9 to 81·1)	−53·1 (−70·4 to −26·1)	31·96 (27·47 to 36·66)	13·15 (8·55 to 17·32)	−53·0 (−70·2 to −26·2)	41 662·6 (34 094·1 to 49 368·7)	16·2 (13·2 to 19·2)	−23·5 (−39·8 to −0·8)	88·59 (83·06 to 94·30)	19·47 (14·54 to 24·11)	−42·2 (−57·8 to −17·6)
Kiribati	16·3 (7·1 to 31·0)	108·9 (47·4 to 207·8)	−37·1 (−71·3 to 24·9)	0·04 (0·03 to 0·04)	0·01 (0·01 to 0·03)	−36·9 (−71·0 to 24·9)	55·3 (42·5 to 72·0)	49·1 (37·8 to 63·9)	−5·2 (−27·5 to 23·3)	0·10 (0·09 to 0·10)	0·03 (0·02 to 0·04)	−20·6 (−47·3 to 22·2)
Laos	2384·5 (1464·1 to 3692·6)	285·2 (175·1 to 441·7)	−50·7 (−68·3 to −24·8)	1·08 (0·91 to 1·27)	2·05 (1·26 to 3·18)	−50·6 (−68·3 to −24·7)	4242·3 (3189·9 to 5609·0)	62·4 (46·9 to 82·5)	−37·1 (−52·4 to −17·9)	2·58 (2·37 to 2·79)	2·53 (1·75 to 3·64)	−46·1 (−62·6 to −23·5)
Malaysia	241·3 (171·1 to 337·9)	9·9 (7·0 to 13·8)	−26·0 (−47·9 to 6·0)	1·93 (1·65 to 2·26)	0·21 (0·15 to 0·29)	−25·6 (−47·3 to 6·1)	16 229·1 (10 698·0 to 20 184·5)	53·6 (35·3 to 66·6)	33·9 (11·8 to 56·4)	10·27 (9·74 to 10·84)	3·45 (2·25 to 4·23)	21·5 (3·5 to 40·2)
Maldives	5·6 (3·8 to 7·5)	15·2 (10·3 to 20·4)	−53·5 (−69·3 to −27·1)	0·03 (0·02 to 0·03)	0 (0 to 0·01)	−53·3 (−69·1 to −27·2)	39·8 (32·3 to 47·8)	11·0 (8·9 to 13·2)	5·2 (−12·2 to 26·6)	0·08 (0·08 to 0·09)	0·01 (0·01 to 0·01)	−33·9 (−47·3 to −15·4)
Marshall Islands	5·1 (2·7 to 9·0)	53·9 (28·6 to 96·0)	−58·9 (−80·1 to −15·2)	0·02 (0·02 to 0·03)	0 (0 to 0·01)	−58·7 (−79·9 to −15·1)	24·7 (19·5 to 31·4)	34·2 (26·9 to 43·4)	−14·2 (−34·8 to 10·1)	0·06 (0·06 to 0·07)	0·01 (0·01 to 0·01)	−36·0 (−56·8 to −3·9)
Mauritius	11·5 (7·5 to 14·4)	16·1 (10·5 to 20·3)	−41·3 (−60·1 to −17·3)	0·07 (0·06 to 0·08)	0·01 (0·01 to 0·01)	−41·2 (−59·6 to −17·4)	329·1 (299·5 to 360·9)	25·8 (23·5 to 28·3)	16·8 (4·4 to 31·2)	0·37 (0·35 to 0·39)	0·06 (0·06 to 0·07)	−5·3 (−14·2 to 5·5)
Myanmar	7711·0 (5428·4 to 11 216·4)	164·3 (115·6 to 238·9)	−66·7 (−76·4 to −51·8)	5·39 (4·60 to 6·39)	6·63 (4·67 to 9·63)	−66·7 (−76·4 to −51·7)	19 334·9 (14 032·8 to 26 083·2)	35·8 (26·0 to 48·3)	−44·1 (−56·3 to −28·4)	17·25 (16·09 to 18·43)	9·89 (7·49 to 13·18)	−57·5 (−67·3 to −43·2)
North Korea	1111·7 (527·0 to 2266·1)	63·5 (30·1 to 129·4)	−54·7 (−81·5 to 13·7)	2·38 (2·06 to 2·78)	0·96 (0·46 to 1·96)	−54·5 (−81·4 to 13·3)	4733·0 (3418·1 to 6426·8)	18·8 (13·6 to 25·5)	−10·4 (−42·6 to 33·1)	8·53 (8·05 to 9·04)	1·65 (1·08 to 2·64)	−39·8 (−68·6 to 14·0)
Northern Mariana Islands	0·9 (0·5 to 1·6)	11·5 (6·3 to 19·6)	−24·5 (−60·5 to 41·3)	0·02 (0·02 to 0·02)	0 (0 to 0)	−23·0 (−58·8 to 41·6)	10·2 (8·7 to 12·4)	8·8 (7·5 to 10·7)	24·8 (6·4 to 44·6)	0·06 (0·06 to 0·07)	0 (0 to 0)	18·7 (−3·7 to 44·2)
Papua New Guinea	2171·2 (1182·6 to 3576·7)	215·0 (117·1 to 354·2)	−39·9 (−62·5 to −8·9)	3·17 (2·71 to 3·72)	1·87 (1·03 to 3·08)	−39·8 (−62·3 to −8·8)	5468·3 (3725·9 to 7950·2)	71·6 (48·8 to 104·2)	−13·4 (−34·7 to 14·9)	7·77 (7·20 to 8·40)	2·94 (1·91 to 4·38)	−27·2 (−48·4 to 1·3)
Philippines	9241·2 (7631·2 to 11 024·5)	81·4 (67·2 to 97·1)	−34·9 (−46·4 to −18·2)	15·48 (13·52 to 17·47)	7·94 (6·56 to 9·47)	−34·9 (−46·3 to −18·2)	54 915·4 (49 977·8 to 59 978·2)	54·5 (49·6 to 59·5)	14·8 (4·2 to 26·3)	46·97 (44·35 to 49·74)	17·94 (16·26 to 19·65)	−12·1 (−21·3 to 0·1)
Samoa	4·3 (1·8 to 8·6)	17·4 (7·3 to 34·4)	−48·3 (−73·2 to −0·8)	0·07 (0·06 to 0·08)	0 (0 to 0·01)	−47·4 (−72·1 to −0·9)	59·1 (44·7 to 76·8)	30·5 (23·1 to 39·6)	2·4 (−19·6 to 29·7)	0·18 (0·17 to 0·19)	0·02 (0·01 to 0·02)	−18·3 (−37·7 to 4·6)
Seychelles	3·1 (2·3 to 4·0)	37·4 (27·5 to 47·8)	−26·1 (−43·0 to −7·2)	0·01 (0·01 to 0·01)	0 (0 to 0)	−26·1 (−42·8 to −7·2)	67·6 (58·6 to 77·5)	70·1 (60·7 to 80·3)	10·1 (−2·6 to 26·5)	0·04 (0·04 to 0·04)	0·01 (0·01 to 0·02)	−3·7 (−14·9 to 9·4)
Solomon Islands	51·4 (31·6 to 82·6)	61·2 (37·6 to 98·3)	−37·3 (−62·4 to 9·7)	0·20 (0·18 to 0·24)	0·04 (0·03 to 0·07)	−37·2 (−62·0 to 9·0)	237·6 (156·6 to 344·2)	40·6 (26·7 to 58·7)	−1·7 (−24·8 to 29·2)	0·49 (0·46 to 0·53)	0·1 (0·07 to 0·14)	−17·2 (−40·4 to 15·3)
Sri Lanka	156·9 (112·5 to 226·7)	9·4 (6·7 to 13·5)	−71·0 (−78·9 to −58·7)	1·14 (0·97 to 1·34)	0·14 (0·1 to 0·2)	−70·7 (−78·7 to −58·5)	4424·3 (3597·2 to 5342·5)	21·3 (17·3 to 25·7)	−14·5 (−30·8 to 4·1)	5·91 (5·61 to 6·23)	1 (0·82 to 1·2)	−31·9 (−44·3 to −17·4)
Taiwan (province of China)	30·9 (18·0 to 49·1)	3·0 (1·8 to 4·8)	−20·8 (−51·9 to 31·0)	0·23 (0·20 to 0·27)	0·03 (0·02 to 0·04)	−20·3 (−51·2 to 30·9)	11 218·4 (5666·3 to 14 514·4)	46·3 (23·4 to 59·9)	68·6 (29·4 to 104·3)	2·50 (2·40 to 2·62)	1·26 (0·65 to 1·6)	38·9 (2·5 to 67·5)
Thailand	292·1 (202·7 to 411·8)	7·8 (5·4 to 11·0)	−70·3 (−79·1 to −58·4)	3·83 (3·31 to 4·41)	0·26 (0·18 to 0·36)	−69·9 (−78·5 to −58·1)	59 313·2 (39 833·0 to 74 843·2)	87·4 (58·7 to 110·2)	36·5 (9·3 to 64·1)	27·85 (26·59 to 29·13)	9·47 (6·3 to 11·57)	9·1 (−9·8 to 29·0)
Timor-Leste	304·8 (136·5 to 555·4)	146·1 (65·4 to 266·3)	−48·9 (−77·7 to 8·8)	0·22 (0·19 to 0·26)	0·26 (0·12 to 0·48)	−48·8 (−77·6 to 8·8)	578·8 (377·4 to 856·6)	48·7 (31·7 to 72·0)	−26·4 (−52·0 to 22·7)	0·48 (0·44 to 0·52)	0·33 (0·18 to 0·54)	−43·0 (−69·7 to 11·2)
Tonga	4·8 (2·7 to 8·1)	35·8 (20·6 to 61·3)	−36·2 (−59·9 to 1·2)	0·03 (0·02 to 0·03)	0 (0 to 0·01)	−36·0 (−59·6 to 1·1)	47·4 (37·1 to 59·3)	44·5 (34·8 to 55·6)	1·7 (−16·8 to 25·6)	0·08 (0·07 to 0·09)	0·01 (0·01 to 0·02)	−16·1 (−32·2 to 3·8)
Vanuatu	34·8 (20·9 to 54·6)	106·3 (63·8 to 166·5)	−31·0 (−59·5 to 25·7)	0·08 (0·07 to 0·09)	0·03 (0·02 to 0·05)	−30·9 (−59·3 to 25·2)	129·5 (93·5 to 180·0)	49·3 (35·6 to 68·5)	2·5 (−20·9 to 36·1)	0·21 (0·20 to 0·23)	0·06 (0·04 to 0·08)	−13·2 (−37·9 to 28·6)
Vietnam	3231·8 (2474·6 to 4210·4)	41·6 (31·9 to 54·2)	−38·4 (−52·3 to −17·9)	10·94 (9·41 to 12·51)	2·8 (2·14 to 3·64)	−38·3 (−52·2 to −17·9)	22 795·3 (17 090·9 to 30 499·6)	24·4 (18·3 to 32·6)	6·4 (−19·6 to 37·7)	37·93 (35·91 to 40·01)	5·68 (4·66 to 6·98)	−20·6 (−35·1 to −2·5)
**Sub-Saharan Africa**	**340 225·0 (302 298·2 to 384 616·7)**	**215·1 (191·2 to 243·2)**	**−21·4 (−30·8 to −11·0)**	**273·92 (240·37 to 310·52)**	**292·1 (259·7 to 329·86)**	**−21·4 (−30·7 to −11·0)**	**732 180·4 (658 747·7 to 803 661·1)**	**76·2 (68·6 to 83·7)**	**−4·7 (−12·4 to 4·7)**	**619·57 (576·11 to 662·79)**	**410·09 (372·07 to 452·23)**	**−13·4 (−21·7 to −3·8)**
Angola	11 621·2 (7439·3 to 17 076·9)	234·9 (150·4 to 345·2)	−17·5 (−48·0 to 24·1)	9·83 (8·29 to 11·59)	9·97 (6·4 to 14·63)	−17·4 (−47·8 to 24·2)	21 837·3 (13 535·6 to 36 689·0)	86·5 (53·6 to 145·3)	−0·8 (−36·0 to 45·0)	20·77 (19·03 to 22·82)	13·31 (8·69 to 19·6)	−9·3 (−38·9 to 32·6)
Benin	4026·1 (2902·7 to 5328·2)	231·1 (166·6 to 305·8)	−21·8 (−43·0 to 9·1)	2·63 (2·29 to 3·00)	3·46 (2·5 to 4·57)	−21·7 (−42·9 to 9·1)	8993·1 (6325·5 to 12 248·5)	82·4 (58·0 to 112·2)	0·4 (−26·4 to 37·6)	6·28 (5·83 to 6·77)	4·97 (3·74 to 6·38)	−11·0 (−32·7 to 17·9)
Botswana	114·6 (64·4 to 182·1)	43·6 (24·5 to 69·2)	−36·5 (−59·3 to 2·8)	0·36 (0·31 to 0·41)	0·1 (0·06 to 0·16)	−36·3 (−59·1 to 3·0)	1344·0 (581·9 to 3751·4)	59·5 (25·8 to 166·0)	4·4 (−58·2 to 182·5)	1·26 (1·19 to 1·35)	0·48 (0·21 to 1·39)	−8·3 (−59·1 to 114·8)
Burkina Faso	10 071·3 (7400·8 to 13 499·9)	321·5 (236·3 to 431·0)	−14·0 (−39·2 to 20·0)	4·00 (3·47 to 4·66)	8·64 (6·35 to 11·57)	−13·9 (−39·1 to 20·1)	16 363·2 (12 432·8 to 20 752·0)	90·4 (68·7 to 114·7)	−2·4 (−27·2 to 28·8)	8·87 (8·18 to 9·63)	10·75 (8·19 to 13·9)	−7·8 (−31·7 to 22·3)
Burundi	5261·2 (3329·3 to 8017·6)	247·0 (156·3 to 376·4)	0·9 (−37·5 to 58·6)	5·18 (4·39 to 6·03)	4·52 (2·86 to 6·87)	1·0 (−37·3 to 58·2)	9826·4 (6999·3 to 13 314·4)	87·4 (62·2 to 118·4)	9·4 (−21·0 to 49·2)	10·34 (9·47 to 11·32)	5·95 (4·18 to 8·44)	5·6 (−25·5 to 50·3)
Cameroon	9541·0 (6734·7 to 13 325·4)	251·4 (177·5 to 351·2)	−7·8 (−35·5 to 31·6)	5·28 (4·58 to 6·04)	8·19 (5·8 to 11·44)	−7·8 (−35·3 to 31·3)	20 101·2 (14 706·5 to 26 891·9)	85·9 (62·8 to 114·9)	1·5 (−26·2 to 37·4)	12·23 (11·30 to 13·20)	11·19 (8·29 to 14·71)	−2·9 (−28·8 to 29·7)
Cape Verde	30·1 (22·1 to 40·0)	56·2 (41·3 to 74·9)	−42·1 (−59·5 to −14·6)	0·08 (0·07 to 0·09)	0·03 (0·02 to 0·03)	−42·0 (−59·3 to −14·7)	251·1 (198·8 to 310·9)	48·3 (38·2 to 59·7)	−4·0 (−28·1 to 33·1)	0·27 (0·25 to 0·29)	0·07 (0·06 to 0·08)	−25·3 (−42·1 to −1·7)
Central African Republic	3222·2 (2084·7 to 4755·2)	453·1 (293·1 to 668·6)	9·8 (−31·1 to 79·6)	1·79 (1·51 to 2·10)	2·77 (1·79 to 4·08)	9·7 (−30·9 to 79·7)	7082·6 (4798·9 to 9918·4)	144·4 (97·9 to 202·3)	16·0 (−18·2 to 63·1)	4·52 (4·18 to 4·87)	3·86 (2·72 to 5·38)	13·7 (−22·7 to 65·2)
Chad	13 589·9 (9620·8 to 18 424·0)	511·3 (361·9 to 693·1)	11·9 (−20·8 to 59·6)	7·17 (6·18 to 8·24)	11·64 (8·25 to 15·78)	11·8 (−20·7 to 59·5)	19 668·5 (14 439·0 to 25 521·5)	139·9 (102·7 to 181·5)	15·6 (−14·9 to 54·7)	13·04 (11·93 to 14·18)	13·57 (9·97 to 17·61)	14·5 (−16·4 to 57·5)
Comoros	163·7 (97·5 to 261·4)	133·3 (79·4 to 212·9)	−35·3 (−58·9 to 7·3)	0·22 (0·19 to 0·26)	0·14 (0·08 to 0·23)	−35·2 (−58·7 to 7·1)	446·0 (326·2 to 602·4)	56·3 (41·2 to 76·1)	−5·7 (−33·3 to 34·2)	0·55 (0·50 to 0·60)	0·23 (0·16 to 0·32)	−20·7 (−44·7 to 19·2)
Congo (Brazzaville)	932·8 (576·1 to 1398·1)	121·5 (75·1 to 182·2)	−27·2 (−54·4 to 13·1)	1·03 (0·90 to 1·18)	0·8 (0·5 to 1·2)	−27·1 (−54·3 to 13·1)	3250·8 (2317·1 to 4502·7)	70·2 (50·1 to 97·3)	3·5 (−26·1 to 48·3)	2·53 (2·36 to 2·69)	1·41 (1·02 to 1·92)	−11·7 (−37·9 to 27·1)
Côte d'Ivoire	9363·6 (6821·8 to 12 702·1)	256·3 (186·7 to 347·7)	−7·4 (−33·2 to 31·2)	5·07 (4·41 to 5·87)	8·06 (5·87 to 10·93)	−7·4 (−33·1 to 31·0)	20 428·5 (15 324·1 to 27 870·1)	90·0 (67·5 to 122·8)	4·4 (−22·9 to 44·2)	12·26 (11·35 to 13·20)	11·41 (8·77 to 14·74)	−1·6 (−26·0 to 31·6)
Democratic Republic of the Congo	38 357·4 (25 735·0 to 53 739·2)	273·5 (183·5 to 383·2)	−8·2 (−40·7 to 36·1)	38·20 (24·25 to 32·24)	32·95 (22·14 to 46·13)	−8·1 (−40·7 to 35·9)	72 827·3 (53 663·3 to 95 077·9)	94·1 (69·3 to 122·8)	8·6 (−16·8 to 45·8)	60·75 (55·93 to 65·54)	42·91 (31·65 to 56·67)	−0·8 (−28·5 to 38·2)
Djibouti	225·1 (140·4 to 314·9)	215·8 (134·6 to 301·9)	−38·9 (−61·6 to −9·3)	0·20 (0·17 to 0·24)	0·19 (0·12 to 0·27)	−38·8 (−61·5 to −9·3)	668·1 (437·2 to 1008·9)	75·1 (49·1 to 113·3)	−8·6 (−41·6 to 51·4)	0·61 (0·57 to 0·66)	0·32 (0·22 to 0·45)	−25·5 (−49·4 to 10·5)
Equatorial Guinea	233·7 (121·8 to 416·5)	182·3 (95·0 to 325·0)	−16·0 (−48·0 to 32·4)	0·24 (0·20 to 0·28)	0·2 (0·1 to 0·36)	−15·8 (−47·8 to 32·4)	598·3 (344·6 to 1165·9)	70·8 (40·8 to 138·0)	2·4 (−38·0 to 63·7)	0·61 (0·57 to 0·66)	0·31 (0·18 to 0·52)	−6·2 (−37·1 to 42·7)
Eritrea	1996·5 (1331·0 to 2837·9)	240·7 (160·5 to 342·2)	−12·5 (−40·9 to 20·7)	2·46 (2·11 to 2·86)	1·71 (1·14 to 2·43)	−12·5 (−40·7 to 20·1)	4192·0 (2752·6 to 5955·7)	80·0 (52·5 to 113·6)	9·4 (−21·3 to 48·5)	5·65 (5·19 to 6·10)	2·44 (1·66 to 3·31)	−2·6 (−30·2 to 30·5)
Ethiopia	25 970·5 (17 970·8 to 34 477·7)	177·4 (122·7 to 235·5)	−54·1 (−70·1 to −31·8)	25·38 (22·27 to 28·52)	22·31 (15·47 to 29·62)	−54·0 (−70·0 to −31·7)	58 231·2 (42 874·1 to 77 649·7)	58·6 (43·1 to 78·1)	−32·4 (−52·5 to −4·5)	60·64 (56·46 to 65·03)	31·81 (23·35 to 40·99)	−45·0 (−61·6 to −21·3)
Gabon	211·0 (136·1 to 306·8)	87·9 (56·7 to 127·8)	−29·6 (−51·9 to 4·0)	0·40 (0·34 to 0·46)	0·18 (0·12 to 0·26)	−29·4 (−51·7 to 3·9)	1193·9 (820·6 to 1730·0)	69·2 (47·5 to 100·2)	−4·6 (−32·7 to 39·6)	1·18 (1·11 to 1·26)	0·4 (0·29 to 0·54)	−14·8 (−37·0 to 18·9)
Ghana	4682·3 (3268·3 to 6359·1)	115·2 (80·4 to 156·5)	−23·8 (−45·9 to 5·7)	6·35 (5·41 to 7·39)	4·02 (2·81 to 5·46)	−23·7 (−45·8 to 5·6)	19 051·7 (12 499·4 to 28 341·1)	69·5 (45·6 to 103·4)	3·7 (−29·2 to 54·7)	15·68 (14·51 to 16·81)	8·13 (5·85 to 11·32)	−7·5 (−33·1 to 27·9)
Guinea	7135·2 (5258·5 to 9358·8)	354·9 (261·5 to 465·4)	−20·2 (−42·4 to 7·6)	3·48 (2·98 to 4·04)	6·12 (4·51 to 8·02)	−20·2 (−42·3 to 7·8)	13 571·0 (10 473·1 to 17 346·0)	107·9 (83·3 to 138·0)	−2·5 (−25·5 to 27·9)	8·09 (7·46 to 8·74)	8·0 (6·28 to 10·1)	−12·2 (−33·4 to 13·9)
Guinea–Bissau	1150·3 (798·5 to 1605·7)	392·6 (272·6 to 548·1)	−13·8 (−40·2 to 25·6)	0·45 (0·38 to 0·53)	0·98 (0·68 to 1·37)	−13·7 (−40·0 to 25·4)	2223·4 (1370·9 to 3917·0)	120·3 (74·2 to 211·9)	−1·3 (−37·4 to 45·6)	1·05 (0·96 to 1·15)	1·32 (0·91 to 1·98)	−7·2 (−35·0 to 27·9)
Kenya	11 999·1 (10 010·8 to 14 286·0)	164·2 (137·0 to 195·5)	−15·9 (−26·9 to −3·2)	12·68 (11·03 to 14·55)	10·33 (8·62 to 12·29)	−15·8 (−26·7 to −3·2)	26 842·8 (22 886·8 to 30 326·6)	58·1 (49·5 to 65·7)	3·6 (−7·0 to 14·7)	31·08 (28·64 to 33·43)	14·52 (12·47 to 16·73)	−7·0 (−16·3 to 4·2)
Lesotho	680·6 (471·5 to 942·7)	249·8 (173·0 to 345·9)	−26·9 (−48·5 to 2·8)	0·41 (0·36 to 0·47)	0·59 (0·41 to 0·81)	−26·8 (−48·4 to 2·9)	2194·0 (1493·8 to 3165·8)	103·0 (70·2 to 148·7)	−8·6 (−35·0 to 26·8)	1·28 (1·20 to 1·37)	1·04 (0·75 to 1·42)	−15·0 (−37·5 to 14·6)
Liberia	1499·6 (1051·2 to 2016·2)	212·4 (148·9 to 285·5)	−30·5 (−52·4 to −2·3)	1·21 (1·02 to 1·40)	1·29 (0·91 to 1·73)	−30·5 (−52·3 to −2·2)	3223·6 (2404·7 to 4177·0)	71·5 (53·3 to 92·6)	−6·9 (−30·1 to 26·1)	2·87 (2·63 to 3·11)	1·8 (1·32 to 2·3)	−20·1 (−41·1 to 6·4)
Madagascar	7804·0 (5361·9 to 11 103·9)	209·4 (143·9 to 297·9)	−10·1 (−40·5 to 34·7)	7·57 (6·50 to 8·77)	6·71 (4·63 to 9·55)	−10·1 (−40·3 to 34·7)	17 759·4 (12 659·9 to 23 847·2)	73·4 (52·3 to 98·6)	8·9 (−20·7 to 46·2)	17·53 (16·08 to 18·96)	9·89 (7·17 to 13·2)	−0·4 (−28·5 to 35·9)
Malawi	8105·2 (5711·7 to 11 070·3)	274·5 (193·4 to 374·9)	−10·0 (−37·7 to 24·3)	6·10 (5·26 to 6·98)	6·96 (4·91 to 9·5)	−10·0 (−37·6 to 24·4)	14 233·9 (10 768·6 to 18 529·2)	82·7 (62·6 to 107·6)	−0·6 (−25·4 to 32·8)	12·85 (11·78 to 13·93)	8·74 (6·66 to 11·24)	−6·4 (−29·7 to 24·5)
Mali	6155·6 (4284·8 to 8654·0)	190·0 (132·3 to 267·1)	−12·6 (−40·0 to 26·1)	4·58 (3·96 to 5·34)	5·28 (3·69 to 7·41)	−12·6 (−39·7 to 26·2)	8654·7 (6578·1 to 11 460·2)	49·3 (37·4 to 65·2)	−5·0 (−28·4 to 26·5)	9·33 (8·52 to 10·20)	6·17 (4·56 to 8·39)	−8·4 (−33·7 to 27·9)
Mauritania	890·1 (654·8 to 1161·0)	144·0 (105·9 to 187·8)	−33·2 (−51·4 to −6·2)	1·05 (0·90 to 1·23)	0·76 (0·56 to 0·99)	−33·2 (−51·2 to −6·2)	2301·8 (1683·4 to 3127·0)	56·4 (41·2 to 76·6)	−8·2 (−32·1 to 25·4)	2·58 (2·39 to 2·79)	1·16 (0·88 to 1·47)	−21·9 (−41·2 to 3·7)
Mozambique	6557·9 (4615·6 to 9098·8)	135·8 (95·6 to 188·4)	−35·2 (−55·0 to −9·8)	6·36 (5·51 to 7·28)	5·64 (3·97 to 7·82)	−35·2 (−55·0 to −9·7)	16 219·4 (11 530·9 to 22 061·5)	57·9 (41·2 to 78·8)	−6·8 (−35·8 to 32·6)	15·30 (14·17 to 16·49)	8·51 (6·32 to 11·06)	−22·1 (−42·5 to 4·4)
Namibia	301·7 (187·1 to 449·0)	90·8 (56·3 to 135·1)	−11·0 (−47·6 to 42·9)	0·47 (0·40 to 0·54)	0·26 (0·16 to 0·39)	−10·9 (−47·4 to 42·7)	1323·8 (889·3 to 1953·5)	54·0 (36·3 to 79·6)	−10·5 (−40·1 to 40·1)	1·37 (1·27 to 1·46)	0·56 (0·38 to 0·78)	−12·5 (−40·8 to 28·0)
Niger	14 121·8 (10 022·4 to 19 340·4)	344·5 (244·5 to 471·7)	−16·9 (−43·3 to 16·7)	6·43 (5·53 to 7·40)	12·07 (8·57 to 16·53)	−16·8 (−43·2 to 16·5)	22 000·2 (16 957·4 to 28 386·2)	110·8 (85·4 to 143·0)	−2·1 (−27·1 to 29·7)	12·79 (11·72 to 13·92)	14·54 (11 to 19·3)	−10·7 (−35·7 to 19·8)
Nigeria	59 644·1 (43 761·4 to 80 821·9)	190·3 (139·6 to 257·9)	−23·4 (−46·8 to 9·3)	50·12 (43·56 to 57·15)	51·19 (37·64 to 69·39)	−23·3 (−46·7 to 9·3)	129 528·6 (93 421·9 to 183 585·3)	71·0 (51·2 to 100·6)	−10·5 (−32·9 to 21·0)	97·76 (89·82 to 105·57)	74·69 (56·45 to 99·56)	−15·8 (−37·2 to 12·2)
Rwanda	4079·1 (2804·7 to 5645·0)	237·7 (163·4 to 328·9)	−39·6 (−58·4 to −12·2)	3·25 (2·80 to 3·76)	3·5 (2·41 to 4·84)	−39·6 (−58·3 to −12·3)	8181·3 (6218·1 to 11 024·6)	70·3 (53·5 to 94·8)	−20·5 (−41·7 to 10·2)	7·98 (7·33 to 8·62)	4·74 (3·55 to 6·15)	−30·9 (−49·9 to −4·1)
São Tomé and Príncipe	36·1 (24·9 to 49·0)	119·3 (82·2 to 161·8)	−45·2 (−60·4 to −22·7)	0·05 (0·04 to 0·05)	0·03 (0·02 to 0·04)	−45·2 (−60·2 to −22·7)	129·2 (85·4 to 179·9)	67·6 (44·7 to 94·1)	−16·9 (−39·2 to 9·0)	0·12 (0·11 to 0·13)	0·06 (0·04 to 0·07)	−30·6 (−47·5 to −7·3)
Senegal	3562·8 (2604·9 to 4552·3)	138·0 (100·9 to 176·4)	−31·4 (−51·3 to −6·0)	3·63 (3·22 to 4·07)	3·06 (2·24 to 3·9)	−31·4 (−51·1 to −6·2)	8797·7 (6329·6 to 12 395·3)	58·2 (41·9 to 82·0)	−4·9 (−30·0 to 29·7)	8·28 (7·69 to 8·88)	4·61 (3·54 to 5·89)	−19·1 (−39·4 to 8·3)
Sierra Leone	3612·1 (2458·7 to 5054·6)	356·5 (242·6 to 498·8)	−26·7 (−49·5 to 3·8)	1·96 (1·67 to 2·30)	3·1 (2·11 to 4·34)	−26·6 (−49·4 to 3·7)	6658·3 (5025·4 to 8498·9)	103·0 (77·8 to 131·5)	−12·2 (−35·8 to 13·3)	4·47 (4·14 to 4·85)	4·12 (3·08 to 5·4)	−19·6 (−41·0 to 6·9)
Somalia	11 116·1 (8223·0 to 14 563·7)	546·8 (404·5 to 716·4)	2·8 (−25·8 to 50·0)	4·39 (3·81 to 5·01)	9·52 (7·05 to 12·47)	2·8 (−25·6 to 49·5)	17 021·0 (12 116·0 to 24 645·9)	156·9 (111·7 to 227·2)	8·8 (−18·2 to 53·6)	8·91 (8·22 to 9·65)	11·4 (8·55 to 15·11)	5·7 (−21·4 to 49·6)
South Africa	3306·8 (2645·2 to 4140·6)	62·1 (49·7 to 77·7)	−63·5 (−71·8 to −53·3)	7·29 (6·49 to 8·07)	2·85 (2·28 to 3·56)	−63·4 (−71·7 to −53·2)	35 124·5 (30 300·4 to 39 843·0)	65·4 (56·4 to 74·2)	−14·5 (−25·8 to −3·5)	35·50 (34·07 to 36·84)	10·68 (9·27 to 12·23)	−35·2 (−44·1 to −26·3)
South Sudan	7605·5 (5167·5 to 10 622·6)	399·5 (271·4 to 557·9)	47·6 (−2·4 to 167·0)	4·79 (4·02 to 5·64)	6·52 (4·43 to 9·1)	47·5 (−2·3 to 165·8)	13 757·3 (8562·4 to 22 165·2)	112·0 (69·7 to 180·4)	55·3 (1·6 to 166·9)	11·04 (10·13 to 12·05)	8·35 (5·67 to 11·76)	51·2 (2·4 to 157·4)
Swaziland	303·4 (199·0 to 438·5)	173·3 (113·7 to 250·5)	−46·6 (−64·4 to −22·7)	0·30 (0·26 to 0·36)	0·26 (0·17 to 0·38)	−46·5 (−64·3 to −22·6)	1062·8 (637·6 to 1629·8)	82·5 (49·5 to 126·5)	−17·6 (−47·0 to 22·5)	0·85 (0·79 to 0·91)	0·51 (0·34 to 0·74)	−30·8 (−53·1 to 0·2)
Tanzania	17 712·5 (13 115·7 to 23 577·8)	190·4 (141·0 to 253·5)	−26·3 (−45·6 to −1·7)	14·12 (12·31 to 16·31)	15·22 (11·27 to 20·25)	−26·2 (−45·4 to −1·7)	38 574·2 (28 222·2 to 52 240·4)	72·3 (52·9 to 97·9)	−1·6 (−27·8 to 33·2)	32·02 (29·53 to 34·63)	21·14 (16·22 to 26·77)	−16·2 (−36·0 to 8·3)
The Gambia	457·3 (344·3 to 600·8)	121·6 (91·6 to 159·8)	−26·7 (−46·1 to 0·3)	0·55 (0·47 to 0·64)	0·39 (0·3 to 0·52)	−26·6 (−46·0 to 0·2)	1077·8 (830·0 to 1355·9)	53·9 (41·5 to 67·8)	−6·7 (−27·6 to 21·5)	1·14 (1·04 to 1·24)	0·58 (0·46 to 0·71)	−16·6 (−34·8 to 6·4)
Togo	2229·8 (1647·2 to 2964·3)	192·5 (142·2 to 256·0)	−19·2 (−40·4 to 10·3)	2·53 (2·17 to 2·94)	1·92 (1·42 to 2·55)	−19·1 (−40·2 to 10·3)	5333·9 (3995·9 to 6982·5)	73·0 (54·7 to 95·6)	0·0 (−25·7 to 33·8)	5·63 (5·18 to 6·12)	2·88 (2·22 to 3·73)	−9·0 (−30·3 to 19·1)
Uganda	12 506·9 (8972·5 to 17 330·6)	169·0 (121·3 to 234·2)	−1·0 (−31·2 to 42·0)	16·56 (14·10 to 19·24)	10·74 (7·71 to 14·86)	−1·0 (−31·0 to 42·0)	25 997·1 (18 861·8 to 34 105·5)	66·4 (48·2 to 87·1)	7·7 (−23·1 to 46·3)	33·42 (30·39 to 36·45)	15 (11·39 to 19·56)	4·5 (−23·2 to 39·1)
Zambia	5072·6 (3542·4 to 7114·9)	175·6 (122·7 to 246·4)	−31·3 (−52·9 to −2·6)	4·43 (3·88 to 5·06)	4·36 (3·05 to 6·11)	−31·2 (−52·8 to −2·5)	13 140·0 (9867·0 to 16 934·5)	80·9 (60·7 to 104·2)	−8·7 (−32·5 to 19·3)	9·77 (9·06 to 10·53)	6·81 (5·23 to 8·91)	−20·0 (−40·1 to 6·0)
Zimbabwe	2896·8 (2089·0 to 3836·0)	117·0 (84·4 to 154·9)	5·9 (−26·4 to 48·0)	3·24 (2·79 to 3·71)	2·49 (1·8 to 3·3)	6·0 (−26·2 to 48·1)	10 709·5 (7235·2 to 15 661·0)	68·8 (46·5 to 100·6)	−2·9 (−33·5 to 41·5)	8·33 (7·70 to 8·97)	4·68 (3·41 to 6·42)	0·7 (−28·3 to 38·3)

Data are n or % (95% uncertainty interval). Modelled number of deaths, episodes, and DALYs for each country in children younger than 5 years and for all ages (not age standardised). The percent change in deaths and DALYs is the change in the absolute number between 2005 and 2015. Data are from GBD 2015 estimates for both sexes.^4^, ^5^ DALYs=disability-adjusted life-years.

We calculated that, in 2015, LRIs caused 103·0 million DALYs (95% UI 96·1 million to 109·1 million) in all ages and 60·6 million DALYs (95% UI 56·0 million to 65·6 million) in children younger than 5 years (59% of LRI DALYs in all ages; table 1). We estimated that in 2015, 291·8 million episodes of LRI occurred (95% UI 276·3 million to 307·0 million), of which 101·8 million episodes were in children aged younger than 5 years (95% UI 90·0 million to 114·4 million; table 1).

Although nearly 60% of LRI DALYs were from children younger than 5 years, our findings suggest that LRI mortality was substantial across all ages, and in elderly people in particular. In adults aged 70 years or older, 1·27 million deaths (95% UI 1·15–1·34 million) were estimated to be caused by LRIs in 2015. In some countries, we estimated a much larger number of deaths due to LRIs in older adults (≥70 years) than in children younger than 5 years—eg, in China (172·3 per 100 000 [95% UI 150·3–196·4] in older adults *vs* 29·2 per 100 000 [25·7–34·7] in children aged <5 years), the USA (235·2 per 100 000 [224·0–247·0] *vs* 2·7 per 100 000 [2·4–3·0]), and Japan (613·7 per 100 000 [588·4–639·2] *vs* 2·8 per 100 000 [2·4–3·2]).

**Figure 2:**
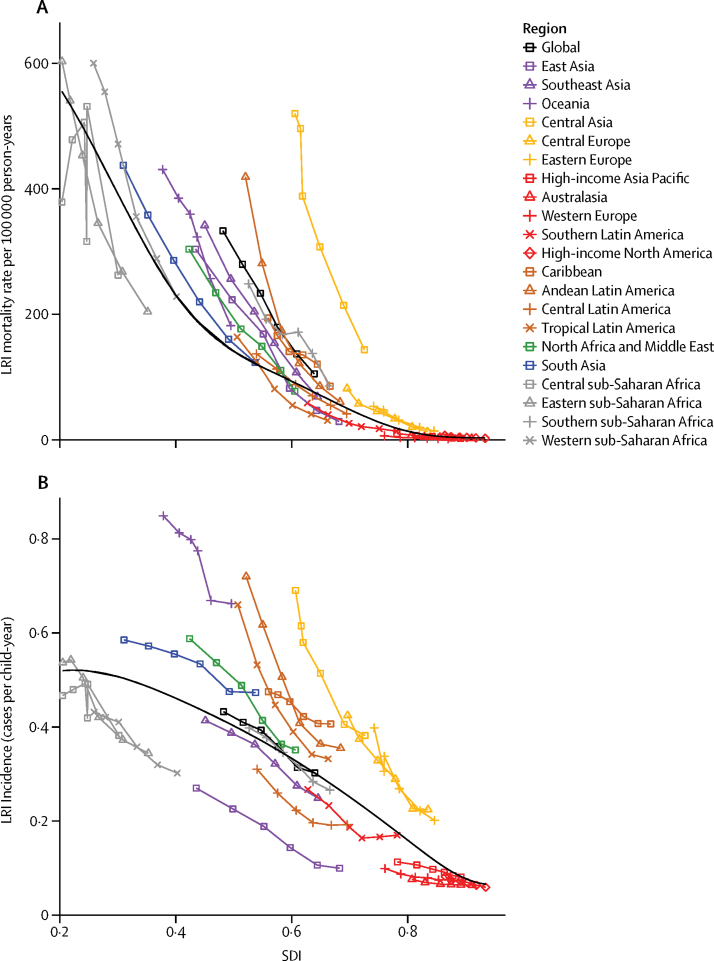
LRI burden by Global Burden of Diseases Study region plotted against SDI Under-5 LRI mortality rate per 100 000 (A) and incidence per child-year (B) is shown. Data points show 5-year increments from 1990 to 2015. The black line is a least-squares cubic spline regression, with knots at 0·4, 0·6, and 0·8, using the under-5 LRI mortality rate or incidence for each geographic location, and represents the expected rate based on SDI alone (estimates above the black line are higher than expected and those below are lower than expected). More information on the formulation and theory of the SDI can be found in the Cause of Death GBD 2015 capstone paper.^5^ LRI=lower respiratory tract infection. SDI=Sociodemographic Index.

The estimated global burden of LRIs decreased greatly between 2005 and 2015, particularly in children younger than 5 years (table 1, Figure 1, Figure 2). During this period, the global number of under-5 deaths due to LRI decreased by 36·9% (95% UI 31·6 to 42·0) from 1·11 million (95% UI 1·03 million to 1·20 million) to 703 918 (651 385 to 763 039), with variation by region and SDI (table 1, figure 1C). However, the total number of LRI deaths decreased by 3·2% (95% UI −0·45 to 6·9; table 1, figure 1D) from 2·83 million (95% UI 2·63 million to 2·97 million) to 2·74 million (2·50 million to 2·86 million) because of a slower decrease in the LRI mortality rate in all ages (14·3% decrease) and population growth and ageing. The LRI mortality rate in all ages increased in many geographies, notably in high-SDI countries, where it increased 9·6% between 2005 and 2015, from 36·2 per 100 000 (95% UI 35·4–37·1) to 39·7 per 100 000 (37·9–41·0).

Between 2005 and 2015, the fastest reduction in under-5 LRI mortality rate occurred in east and southeast Asia, central Europe, and tropical Latin America according to our estimates (>50% reduction; figure 1C). The fastest rate of improvement in under-5 LRI mortality occurred in Turkey (14% average annual decrease; figure 1C). The slowest decreases in under-5 mortality occurred in sub-Saharan Africa (2·1% annual decrease), and mortality increased in South Sudan (0·7% annual increase; figure 1C). We detected a relationship between LRI mortality and incidence and the SDI (figure 2). The LRI mortality rate decreased rapidly when transitioning from low to middle SDI, but the mortality rate in central Asia was much higher than expected on the basis of SDI (figure 2A). The relationship between incidence and SDI appeared to be more linear than for mortality and SDI (figure 2B). Despite reductions in LRI mortality, LRI incidence has decreased at a slower rate than mortality in children younger than 5 years (8·8%, 95% UI 6·6–11·1%) from 0·18 episodes per child-year (95% UI 0·16–0·20) in 2005 to 0·15 episodes per child-year (95% UI 0·13–0·17) in 2015, and in all ages (5·5%, 4·4–6·6) from 0·042 (95% UI 0·039–0·044) in 2005 to 0·040 (0·037–0·042) in 2015.

**Figure 3:**
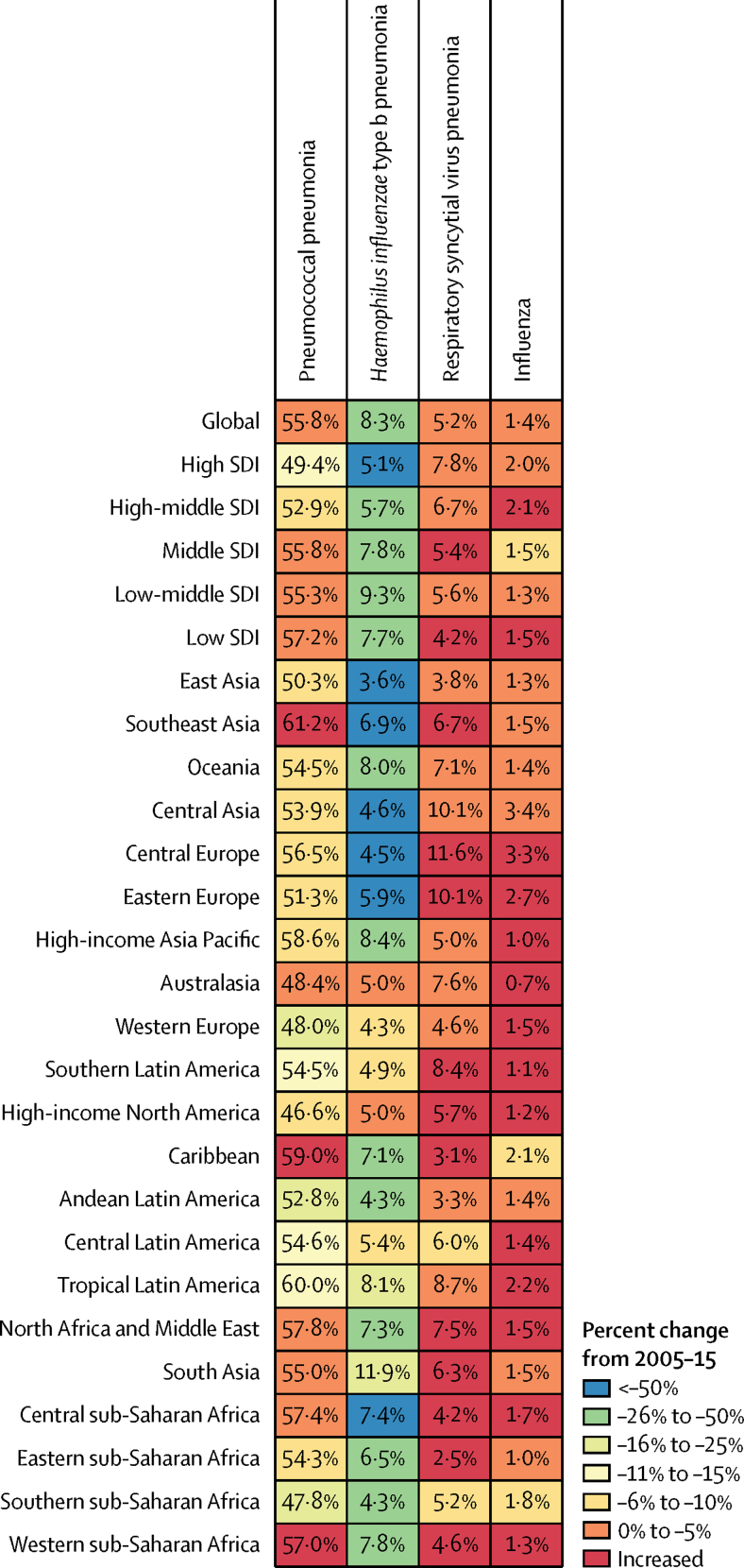
Attributable fraction of LRI mortality in children younger than 5 years in 2015 Aetiologies for each GBD region are ordered by the global ranking. Numbers show the population attributable fraction in 2015, and colours show the percent change from 2005 to 2015. LRI=lower respiratory tract infection.

LRIs were attributed to four aetiologies in GBD 2015.^5^ We estimated that the bacterial causes of LRIs, pneumococcal pneumonia and Hib, together accounted for 64·1% of LRI deaths in children younger than 5 years (table 2). Pneumococcal pneumonia was the most common aetiology, leading to an estimated 392 965 deaths (95% UI 228 367–532 281) or 55·8% (95% UI 32·5–75·0) of LRI deaths in children younger than 5 years, and 1 517 388 deaths (857 940–2 183 791), or 55·4% (31·5–79·1) of LRI deaths in all ages. Syria had the largest percentage of under-5 LRI deaths due to pneumococcal pneumonia (70·6%, 95% UI 43·4–91·8). Pneumococcal pneumonia was also responsible for a substantial number of deaths in the elderly population worldwide: we estimated that in 2015, pneumococcal pneumonia killed 693 041 people aged 70 years and older (95% UI 295 084–1 116 257). The pneumococcal pneumonia PAF in children younger than 5 years was unchanged globally between 2005 and 2015, but decreased in high-SDI regions (figure 3). During the same period, the attributable fraction of LRI deaths in children younger than 5 years due to Hib decreased by 38·6% (95% UI 34·5 to 43·3), from 13·4% (−0·8 to 24·7) in 2005 to 8·3% (−0·5 to 15·9) in 2015 (figure 3). Hib was a major cause of under-5 LRI mortality in India where we estimated that it was responsible for 14·9% (−0·9 to 27·4) of LRI deaths (table 2). Hib was not attributed to any LRI deaths in people older than 5 years.

**Table 2:** Number of deaths and PAFs of LRI-related deaths in children aged 5 years or younger, by aetiology, in top ten countries with highest under-5 LRI mortality burden

	**Pneumococcal pneumonia**		***Haemophilus influenzae* type b**		**Respiratory syncytial virus**		**Influenza**		**LRI deaths unattributed, %**
		Number	PAF, %	Number	PAF, %	Number	PAF, %	Number	PAF, %
Global	392 964·8 (228 367·0 to 532 281·4)	55·8 (32·5 to 75·0)	58 735·8 (−3130·9 to 114 528·3)	8·3 (−0·5 to 15·9)	36 362·5 (20 355·4 to 61 544·9)	5·2 (2·9 to 8·6)	10 150·6 (5 731·1 to 16 789·6)	1·4 (0·8 to 2·4)	29·3 (−1·9 to 63·8)
India	82 448·4 (51 126·5 to 112 117·5)	58·7 (36·6 to 75·8)	20 987·1 (−1164·2 to 39 659·8)	14·9 (−0·9 to 27·4)	8414·9 (4689·0 to 14 116·9)	6·0 (3·4 to 10·1)	2351·7 (1326·4 to 3790·8)	1·7 (1 to 2·7)	18·7 (−16·0 to 59·9)
Nigeria	33 810·9 (17 837·6 to 53 327·8)	56·5 (33·9 to 75·3)	5249·7 (−239·1 to 11 278·1)	8·8 (−0·5 to 17·4)	2297·1 (1040·9 to 4276·5)	3·8 (2 to 6·6)	396·4 (188·9 to 745·6)	0·7 (0·4 to 1·1)	30·2 (−0·4 to 64·2)
Pakistan	20 025·0 (9714·1 to 32 523·3)	50·0 (26·5 to 75·9)	2565·7 (−106·3 to 5580·4)	6·5 (−0·3 to 13·4)	3263·4 (1664·4 to 5706·6)	8·3 (4·6 to 14·3)	556·5 (269·5 to 1057·0)	1·4 (0·8 to 2·5)	32·8 (−6·1 to 68·4)
Democratic Republic of the Congo	21 483·2 (9964·1 to 35 809·1)	55·8 (29 to 80·9)	2966·7 (−147·6 to 6748·8)	7·7 (−0·4 to 15·6)	1612·8 (739·2 to 3251·3)	4·2 (2·3 to 7·4)	677·7 (308·1 to 1309·8)	1·8 (0·9 to 3)	30·5 (−6·9 to 68·2)
Ethiopia	14 148·3 (6769·4 to 22 672·6)	54·2 (30·5 to 76·1)	2121·4 (−100·8 to 4583·1)	8·1 (−0·4 to 16·4)	606·2 (255·1 to 1219·1)	2·3 (1·1 to 4·4)	298·2 (115·4 to 620·2)	1·1 (0·5 to 2·2)	34·3 (0·9 to 68·3)
China	12 177·0 (7171·5 to 16 901·6)	50·2 (30 to 66·7)	857·9 (−42·1 to 1825·4)	3·5 (−0·2 to 7·3)	896·8 (494·2 to 1548·5)	3·7 (2·1 to 6·3)	303·4 (167·8 to 519·5)	1·3 (0·7 to 2·1)	41·3 (17·6 to 67·4)
Bangladesh	8460·8 (4028·1 to 13 627·8)	39·8 (20·4 to 59·8)	733·2 (−32·6 to 1645·8)	3·4 (−0·2 to 7·4)	1258·0 (665·0 to 2238·3)	5·9 (3·2 to 10·5)	154·2 (78·5 to 269·6)	0·7 (0·4 to 1·3)	50·2 (21·0 to 76·2)
Afghanistan	11 920·0 (5681·6 to 19 034·2)	62·2 (36·7 to 84·1)	1848·8 (−93·1 to 4006·6)	9·7 (−0·5 to 18·9)	1702·4 (713·2 to 3350·3)	8·9 (4·2 to 16·6)	318·0 (134·4 to 646·0)	1·7 (0·8 to 3·2)	17·5 (−22·8 to 58·8)
Tanzania	9203·7 (4418·1 to 14 959·4)	51·9 (27·3 to 74·1)	801·1 (−31·0 to 1898·7)	4·5 (−0·2 to 9·6)	450·9 (197·7 to 876·2)	2·5 (1·2 to 4·8)	162·6 (77·9 to 304·9)	0·9 (0·5 to 1·6)	40·2 (9·9 to 71·2)
Indonesia	9573·2 (4522·3 to 14 806·7)	62·5 (38·6 to 82·2)	1298·4 (−73·5 to 2818·6)	8·5 (−0·5 to 16·7)	567·3 (246·7 to 1086·3)	3·7 (1·9 to 6·6)	360·0 (160·4 to 654·2)	2·4 (1·3 to 4)	22·9 (−9·5 to 58·7)

Data are n or % (95% uncertainty interval). The number of deaths in children younger than 5 years are shown for each aetiology at the global level and for each of the ten countries with the highest LRI mortality burden. Aetiological attributable fractions are based on a counterfactual modelling strategy and do not necessarily sum to 100% in a given location. LRI=lower respiratory tract infection. PAF=population attributable fraction.

We estimated that RSV was responsible for 36 363 deaths (20 355–61 545), and influenza was responsible for 10 151 (5 731–16 790) in children younger than 5 years, together accounting for 6·6% of LRI deaths in this age group (table 2). The burdens of RSV were highest in central and eastern Europe and in central Asia, where it accounted for more than 10% of under-5 LRI mortality in 2015 in each of these regions (figure 3); the highest RSV burden was 12·3% (95% UI 6·6–21·7%) in Macedonia. Influenza was not frequently associated with under-5 LRI mortality but was responsible for more than 7% of deaths in all ages in central and eastern Europe and central Asia. The highest attributable fraction due to influenza was in central Asia and central and eastern Europe. The viral aetiologies, RSV and influenza, were more often associated with non-fatal episodes of LRI, largely because of the adjustment for the lower case fatality ratio in viral causes of LRI than bacterial aetiologies. In all ages, 15·4% (95% UI 13·0–18·4%) of incidence was attributable to RSV and 10·4% (8·7–11·9%) to influenza. Between 2005 and 2015, the influenza PAF increased globally in all ages (5·6% increase, 95% UI 0·0–11·0%), and by more than 15% in North Africa, the Middle East, and south Asia.

We estimated that the leading risk factors for LRI DALYs in 2015 were childhood wasting (responsible for 44·6% [95% UI 31·7–52·8] of DALYs worldwide), household air pollution (35·8%, 24·8–45·5), and ambient particulate matter (27·5%, 20·8–34·7). Suboptimal breastfeeding was the third-leading risk factor for under-5 DALYs globally and the leading risk factor for under-5 DALYs in high SDI locations. Other risk factors for LRI, such as smoking, alcohol use, and zinc deficiency, were responsible for less than 10% of LRI DALYs globally.

**Figure 4:**
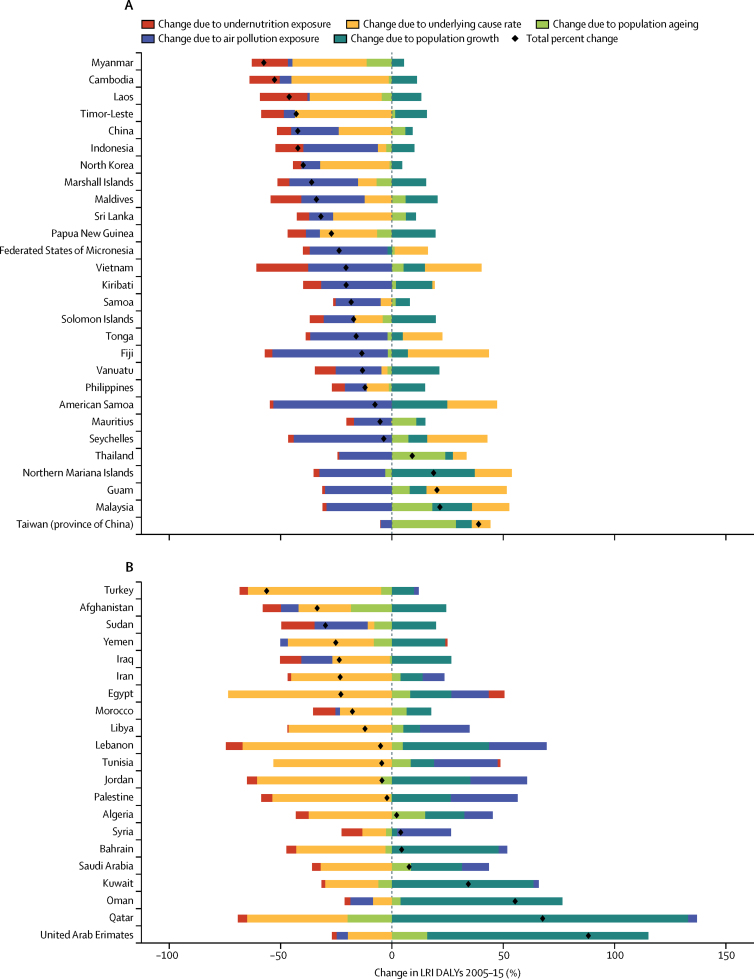

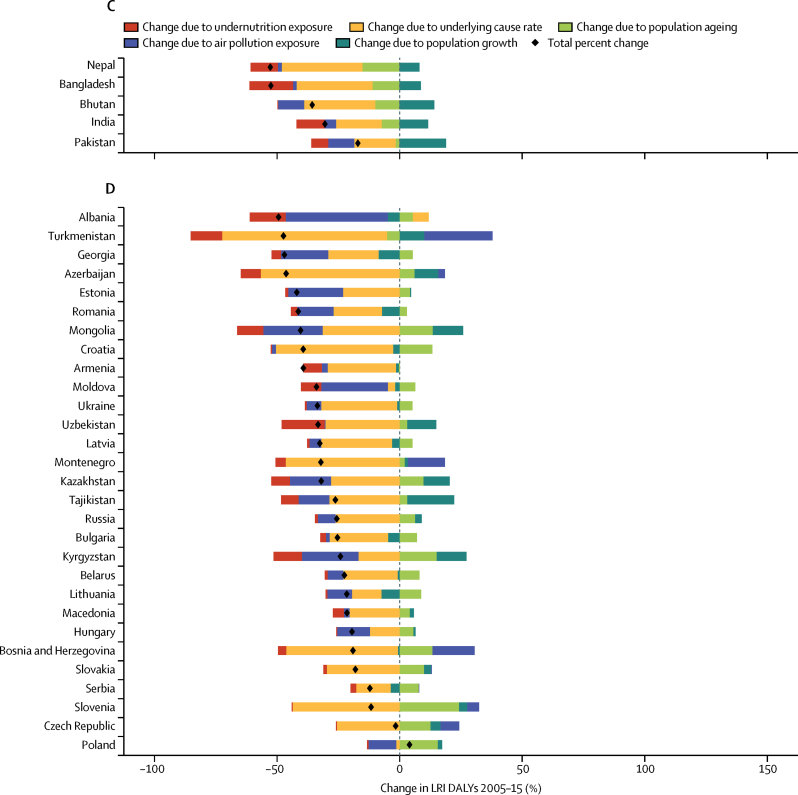

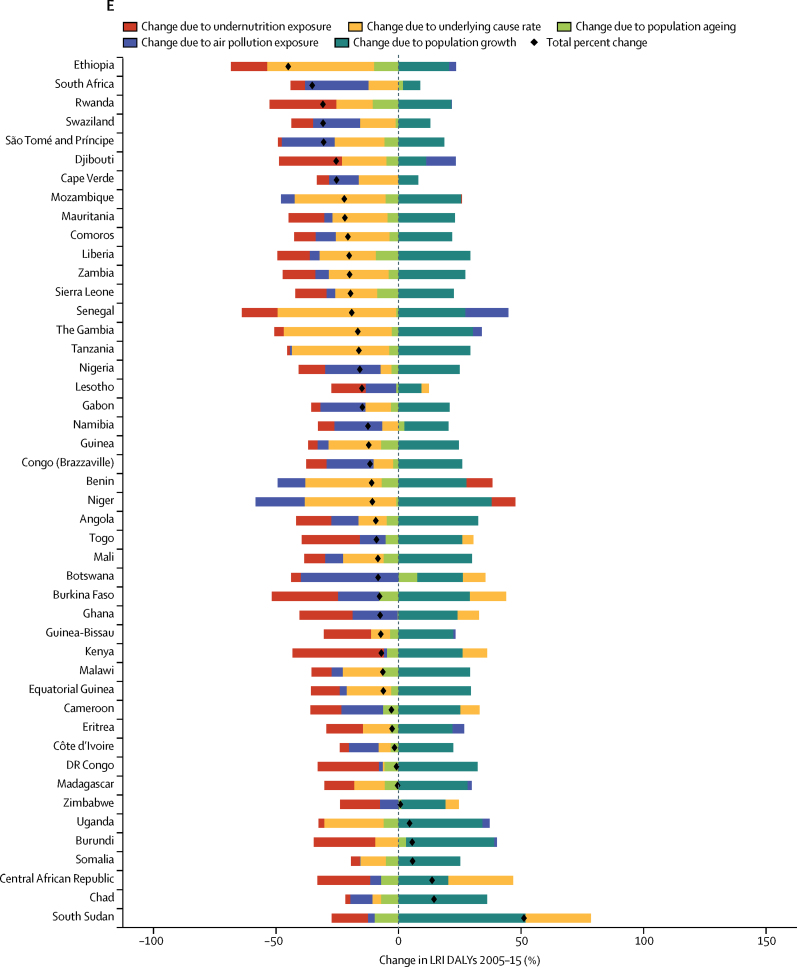

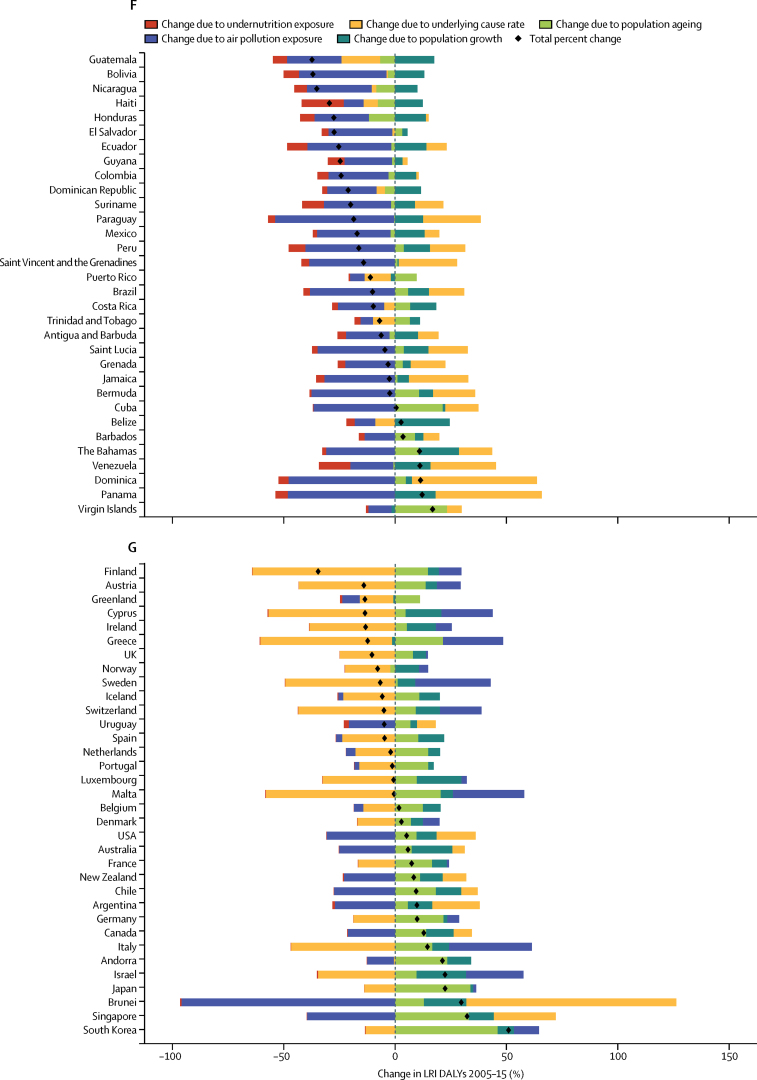
Risk factor decomposition of the change in attributable DALYs in all ages between 2005 and 2015 (A) Southeast Asia, east Asia, and Oceania; (B) north Africa and Middle East; (C) south Asia; (D) central Europe, eastern Europe, and central Asia; (E) sub-Saharan Africa; (F) Latin America and Caribbean; and (G) high-income WHO regions. Black dots show the overall percentage change in LRI DALYs and colours show contribution of different factors to the rate of change. Bars to the left of zero show a reduction in attribution and bars to the right show an increase. LRI=lower respiratory tract infection. DALY=disability-adjusted life-year.

A decomposition of the change in attributable DALYs between 2005 and 2015 by country is shown in figure 4, which includes the two leading risk factors for LRI DALYs, childhood undernutrition and air pollution (indoor and ambient). At the global level, we estimated that LRI DALYs have decreased 8·9% because of reduced prevalence of childhood undernutrition and decreased 4·3% because of improvements in air pollution exposure. We estimated that LRIs attributable to childhood undernutrition have decreased in many countries in sub-Saharan Africa during this period, particularly in Kenya (37·2% decrease), but the number of DALYs in Kenya have only marginally decreased overall, mainly because of population growth (figure 4E). LRI DALYs in many countries in Latin America and the Caribbean have decreased substantially because of reductions in exposure to air pollution, including a 53% reduction attributable to these improvements in air pollution in Paraguay. Population ageing has contributed to a larger burden of LRI DALYs, particularly in high-income countries. In adults aged 70 years and older, DALYs due to LRI have increased by an estimated 18·9% between 2005 and 2015 (data not shown). The increase in LRI DALYs in this age group was highest in low-SDI regions where the number of DALYs increased by 25·0%. All LRI models and results for GBD 2015 can be explored further online using the Institute for Health Metrics and Evaluation visualisations.

## Discussion

The GBD 2015 study estimated that LRIs were the fifth-leading cause of death (of 249 causes) and the leading infectious cause, responsible for 2·74 million deaths (95% UI 2·50 million to 2·86 million). LRIs were the third-leading cause of under-5 mortality behind preterm birth and neonatal encephalopathy, accounting for 12·1% of deaths in this age group. Our analysis suggests that the number of deaths due to LRI in children younger than 5 years decreased by 37% between 2005 and 2015. We found that, although all-age LRI mortality rate decreased, growing and ageing populations have contributed to no significant decline in total LRI deaths between 2005 and 2015. Despite dramatic improvement in the under-5 LRI mortality rate, LRI remains a preventable cause of death in young children and elderly adults, particularly in south Asia and sub-Saharan Africa, and was the second-leading cause of DALYs in 2015. The findings call for renewed efforts to control and prevent LRIs across all age groups.

Some solutions to prevent LRI deaths do not require major advances in technology. Measures to protect, prevent, and treat LRIs are highlighted in the Global Action Plan for Pneumonia and Diarrhoea.^28^ The findings from this study indicate that LRI incidence has declined far more slowly than mortality, suggesting that interventions and treatments that prevent mortality for LRI, particularly in children younger than 5 years, have been much more successful reducing the burden of LRI than prevention of disease incidence. According to this study, the reduction in LRI DALYs can be traced to Hib vaccine use, decreased exposure to indoor air pollution, and a reduction in undernutrition in children younger than 5 years.

Although such interventions were not estimated in GBD 2015, improved access to health care and emphasis on appropriate treatment have probably played a crucial role in reducing LRI mortality, with proper treatment reducing mortality by 20–42%.^2^, ^29^, ^30^, ^31^ How much of the decrease in mortality is due to proper adherence and implementation of the WHO Integrated Management of Childhood Illness recommendations is unclear, because data on its uptake are scarce. These recommendations, which are based on symptom-based screening criteria such as fast breathing or lower chest wall indrawing, have been updated several times, and their application varies substantially. Divergence from these criteria might lead to inappropriate treatment and misuse of antibiotics.^29^, ^32^, ^33^ Our results suggest that most severe LRIs have bacterial causes, whereas pneumococcal pneumonia and Hib have effective Gavi-supported vaccines, emphasising that combined appropriate case management and vaccine use might prevent many episodes of LRI and reduce dependence on antibiotics.^34^

In 2015, approximately 65% of children younger than 5 years received the Hib vaccine and 40% received the PCV.^35^ At the global level, the PAF of Hib on LRI deaths decreased 37·8% between 2005 and 2015, reflecting the expanded use and introduction of the vaccine during this time, particularly in countries that received support from Gavi.^36^ Despite the growing use of PCV, pneumococcal pneumonia mortality has not decreased significantly at the global level and has decreased more slowly than Hib, in part because the PAF for pneumococcal pneumonia depends on the PAF for Hib; as Hib decreases, we assume that pneumococcal pneumonia must increase to account for overall LRI aetiological attribution.

The expanded use of PCV might have several indirect effects on LRI burden. PCV might prevent influenza and RSV mortality, because up to half of severe viral infections are complicated by pneumococcal pneumonia.^37^ Further, PCV might induce large indirect (herd) vaccine effects that protect unvaccinated populations, such as adults and elderly people.^38^ Amid debate about quantifying the effect of indirect vaccine effectiveness for adults in populations with infant vaccine use,^39^ our findings highlight the burden of LRI in the elderly population, including nearly 700 000 deaths in people aged older than 70 years due to pneumococcal pneumonia. Expanding access to the vaccine in adults might substantially reduce the burden of LRI.

Our results suggest that LRIs were the second-leading cause of DALYs globally in 2015 after ischaemic heart disease.^4^ Our results also suggest that decreases in under-5 undernutrition have substantially reduced LRI DALYs, and are responsible for nearly 9% of the decline during this period.^22^, ^40^, ^41^ The greatest reduction in LRI DALYs due to childhood undernutrition between 2005 and 2015 occurred in east and southeast Asia. This finding is notable because improved childhood nutrition will have effects beyond reducing LRI DALYs and is also likely to reduce the burden of disease caused by diarrhoea and measles.^40^ Emphasis on sustainable agriculture, supplementary nutritional programmes, and equitable distribution of food through the Sustainable Development Goals will be necessary for continued reductions in the global burden of LRI.^42^

Household solid fuel use as a risk factor for LRI has decreased since 2005, particularly in Latin America and southeast Asia, which are undergoing rapid urbanisation and economic development. Economic development that shifts energy requirements away from household burning of biomass might reduce exposure to indoor air pollution at the expense of outdoor and ambient particulate matter from large-scale energy production facilities like coal-burning power plants.^43^ Providing affordable clean energy options in low sociodemographic areas of the world is covered by the Sustainable Development Goals, but achieving this aim will be a challenge and the risk of LRIs might depend on its success.^3^

Our estimates of LRI mortality, morbidity, and aetiology attribution are limited by data availability and especially the sparsity of data in sub-Saharan Africa, the region with the greatest LRI burden and need for high-quality data (appendix pp 5, 7, and 14–15). Only extremely scarce verbal autopsy data are available for large populations and the data that are available in Africa and south Asia might be of low quality, as measured by indices such as completeness, detail, internal consistency, and timeliness. Better surveillance systems, including standard reporting mechanisms and case definitions, in Africa and south and southeast Asia would substantially reduce a major source of uncertainty in the LRI mortality estimates.^44^ Assessing a systematic bias in morbidity or mortality estimates is difficult because of data quantity and quality. The predictive modelling approaches used in GBD 2015 rely on covariates and shared information across space and time to fill in these areas and the data gaps are reflected in the uncertainty intervals in the estimates (table 1).

Even with the application and expanded use of PCR diagnostic techniques, data on the aetiology of pneumonia remain sparse, particularly in areas with high disease burden. This scarcity of data is largely due to the difficulty of obtaining appropriate samples for testing, particularly in children, the relatively high cost of PCR, and challenges in culturing and diagnosing many pathogens that cause respiratory infections.^45^, ^46^ Studies that have attempted to elucidate the aetiology of childhood pneumonia, frequently using nasopharyngeal swabs or lung aspirates, have had poor success in identifying an obvious aetiological agent.^45^, ^47^ Atypical pathogens, including nosocomial infections such as *Staphylococcus aureus*, or intracellular pathogens like *Mycoplasma pneumoniae*, might be important aetiologies for LRIs and are not included in GBD 2015.^48^, ^49^ Such omissions might limit the ability to attribute LRI episodes and deaths to pathogens because our analysis is not able to show whether the unattributed LRI episodes and deaths are due to the four aetiologies included in GBD 2015 or other pathogens. Results from the Pneumonia Etiology Research for Child Health Project,^50^ a seven-site case-control study in sub-Saharan Africa and south Asia, were not available for inclusion in GBD 2015 but might provide evidence on LRI aetiologies such as additional pathogens, the relative contribution of each aetiology, and viral–bacterial coinfections.

The attributable fraction strategy for Hib and pneumococcal pneumonia assumes that the vaccine efficacy against invasive disease is the same as for pneumonia. A study by Bonten and colleagues^18^ using a urine antigen test in elderly adults suggests that the vaccine efficacy of PCV13 might be up to a third higher against invasive pneumococcal disease than against pneumococcal pneumonia.^18^ We have adjusted our estimates of vaccine effectiveness from other studies using this ratio but recognise the uncertainty around the application of a single study in elderly adults to all other studies and decided to use a flat distribution centred on the mean ratio from the study to reflect this uncertainty. Application of this diagnostic test is unsuitable for children and is complicated by the frequent nasopharyngeal carriage rate in children, perhaps up to 90% in low-income settings.^45^, ^51^, ^52^ We do not account for serotype replacement or changes in serotype prevalence due to the introduction of PCV, which might be an important factor in the burden of pneumococcal pneumonia and the effectiveness of the vaccine at the population level.^29^

Only four randomised controlled trials on Hib vaccine efficacy have been done in children younger than 5 years. Despite a plausible disease burden in older children and adults, we decided to apply the attributable fraction of LRI episodes and deaths due to Hib pneumonia to the under-5 age group only. The lower bound of the Hib PAF estimates is below zero (not statistically significant) at the global level, reflecting in part the scarcity of reliable data on Hib vaccine efficacy.

The attribution of the viral pathogens to LRI mortality was based on the relative case fatality of bacterial to viral aetiologies, and cases of LRI admitted to hospital might not be representative of cases not admitted to hospital. Efforts to improve surveillance, such as the African Network for Influenza Surveillance and Epidemiology,^53^ are essential in tracking the burden of influenza and other LRI aetiologies and for appropriate and timely response to epidemics. We excluded data describing pandemic H1N1 influenza to avoid biasing global and temporal trends in influenza burden, but doing so might have led to lower estimates of influenza burden, particularly since 2008.

The GBD 2015 estimates of LRI mortality and burden are generally similar to the GBD 2013 estimates.^1^, ^10^ Global under-5 mortality was lower in GBD 2015 than GBD 2013, primarily because of decreased estimates in Nigeria (appendix pp 18–21). Nigeria is a high-population, high-burden country with sparse data and estimates in this high-burden country are influenced by regional trends and covariates; limitations shared by much of sub-Saharan Africa. In fact, only a single datapoint informed cause of death models in this country. Mortality and morbidity were different between GBD versions in China and India (appendix pp 18–21), which reflects in part that these countries are now modelled subnationally (data not shown), allowing for greater accuracy and precision in geographic disparities. Disparities in LRI burden by wealth, geography, and other subpopulation characteristics might be missed when national-level estimates are presented. The GBD study will be produced annually starting with GBD 2016, and future iterations will feature finer spatial resolution, including mapping the burden of LRI on a 5 × 5 km level, which will enable tracking of the burden at a very fine resolution.

Our estimates of pneumonia mortality in children younger than 5 years differ from those produced by the WHO Department of Evidence, Information and Research and the Maternal and Child Epidemiology Estimation (MCEE) group. The GBD 2015 estimates for under-5 mortality due to LRI in 2015 (704 000 deaths, 95% UI 651 000–763 000) were much lower than those from the MCEE (920 000 deaths).^54^, ^55^ The difference in total under-5 deaths was greatest for Nigeria and India (appendix p 25).

Despite substantial reductions in under-5 LRI mortality in many countries, the burden remains high, particularly in areas of low sociodemographic development, and has increased in some populations, particularly elderly people. Estimates of the global burden of LRI will be improved by more high-quality data on mortality, morbidity, and aetiologies, especially in sub-Saharan Africa where the burden is highest and data are most scarce. The creation and expansion of civil registration systems in Africa and south Asia are gaining momentum, and such data will not only improve global comparative mortality assessments such as the GBD study, but also increase the evidence for guiding decision about local policy.^44^, ^56^ Improvements in diagnostics for LRI aetiologies, including those appropriate for children younger than 5 years to better understand the unique contribution of each aetiology to the LRI burden, will help guide targeted interventions such as vaccination. Continuing to emphasise the importance of appropriate case management, to expand the use of PCV, and to reduce childhood undernutrition and exposure to air pollution will accelerate the reduction in LRI disease burden.

Correspondence to: Dr Ali H Mokdad, Institute for Health Metrics and Evaluation, University of Washington, Seattle, WA 98121, USA **mokdaa@uw.edu**
