# Supplementary material for: Estimates of the global, regional, and national morbidity, mortality, and aetiologies of lower respiratory tract infections in 195 countries: a systematic analysis for the Global Burden of Disease Study 2015
Source: Lancet Infect Dis. 2017 Nov;17(11):1133–61. doi: 10.1016/S1473-3099(17)30396-1 (PMC5666185; doi:10.1016/S1473-3099(17)30396-1)
Supplement: Supplementary appendix [file mmc1.pdf]

THE LANCET Infectious Diseases

Supplementary webappendix

This webappendix formed part of the original submission and has been peer reviewed. We post it as supplied by the authors.

Supplement to: GBD 2015 LRI Collaborators. Estimates of the global, regional, and national morbidity, mortality, and aetiologies of lower respiratory tract infections in 195 countries: a systematic analysis for the Global Burden of Disease Study 2015. *Lancet Infect Dis* 2017; published online August 23. http://dx.doi.org/10.1016/S1473-3099(17)30396-1.

Appendix to The global burden of lower respiratory infections: results from the Global Burden of Diseases, Injuries, and Risk Factors (GBD) 2015 Study

This appendix provides methodological detail, supplemental figures, and comprehensive information on input data and data transformation.

Contents

Analytic flowcharts…………………………………..…………………………………………………......….2

Details on mortality modelling……………………………………………………………………………….4

Details on morbidity modelling………………………………………………………………………………6

Details on etiologic attribution………………………………………………………………….……………9

Comparison to GBD 2013……………………………………………………………..…………………………18

Comparison to WHO-MCEE…………………………………………………………………………...………..23

References……………………………………………………………………………..……………………….………26

Flowchart 1. This analytic flowchart shows the lower respiratory infections (LRI) mortality, etiologic attribution, and morbidity strategy. Mortality, morbidity, and etiologic attribution are modeled independently and etiologies are attributed to cases and deaths after those envelopes are estimated. Each of these steps will be described in greater detail in this appendix.

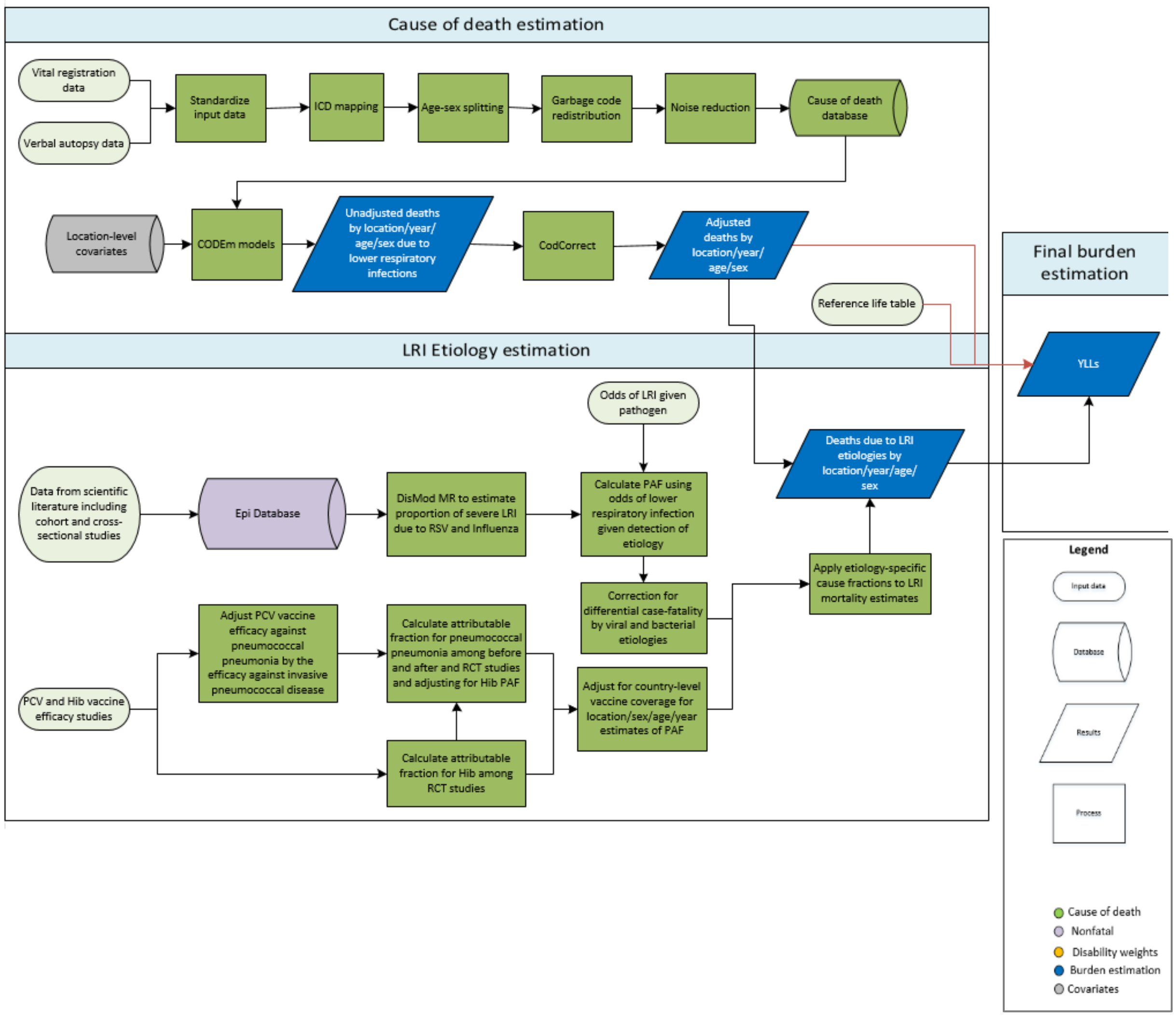

Flowchart 2. This flowchart shows the detailed analytic strategy for the non-fatal LRI modelling including the etiologic attribution to LRI morbidity. Each of these steps will be discussed in greater detail in the section that follows.

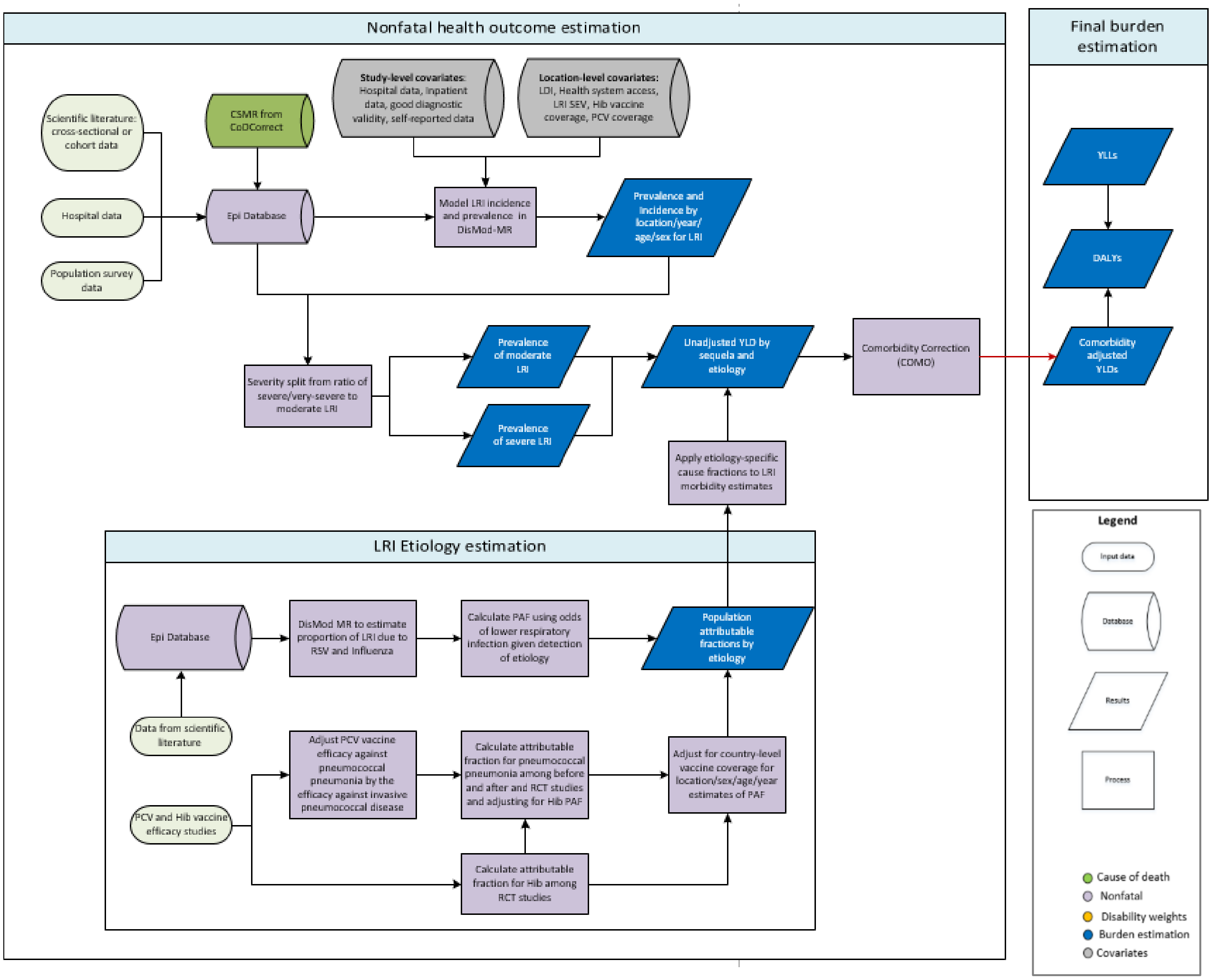

Summary of LRI mortality modelling

Input data were all available data from vital registration systems, surveillance systems, and verbal autopsy. We first identified verbal autopsy studies, irrespective of cause, by searching PubMed and Google Scholar for all studies with the term “verbal autopsy”, and did country-specific searches on Google using the country name and “verbal autopsy”. LRI mortality was identified by ICD9 and ICD10 codes (ICD9 Codes included 073·0-073·6, 079·82, 466-469, 480-489, 513·0, and 770·0. ICD10 codes included A48·1, J09-J22, J85·1, P23-P23·9, and U04). We included studies that used verbal autopsy, had over 50 deaths, provided the number of deaths with LRI as the underlying cause, and were conducted for at least one year to account for seasonality. A summary of the input data is shown in **Appendix Figure 1.** There were 515,000 data points on LRI mortality that were used in the modelling. We checked for and excluded outliers from our data by country or region. We excluded ICD9-coded mortality data in Sri Lanka (1982, 1987–1992), ICD9-coded neonatal mortality data in Guatemala (1980, 1981, 1984, 2000–2004), and Civil Registration System data in some Indian states (1986–1995). Overall, 3013 data points were excluded or outliered (0·6% of data points).

A key component of cause of death modeling in GBD is the redistribution of poorly coded causes of death such as “infection”, “fever”, or “dehydration” to specific causes of death.[1] This processing of *garbage* codes, which means causes of death that cannot or should not be considered underlying causes of death, reallocates a number of deaths from these non-specific causes to LRI. The garbage code redistribution was informed by an IHME expert review of the data and subsequent modeling.[1] Data points were split into GBD age groups using the global mortality age pattern for LRI. An overall mortality envelope and population estimates by age, sex, and location were used to calculate cause fraction and mortality rate.

LRI mortality was estimated in the Cause of Death Ensemble model (CODEm) platform.[2,3] CODEm is a Bayesian statistical model and uses spatial priors from a hierarchical structure to inform the mortality models. CODEm is based on five general principles: 1) identifying all available data, 2) maximizing the comparability and quality of the dataset, 3) developing a diverse set of plausible models, 4) assessing the predictive validity of each plausible individual model and of ensemble models, and 5) choosing the model or ensemble model with the best performance in out-of-sample predictive analysis. Each sub-model is evaluated using in- and out-of-sample predictive validity (15% of data are withheld during the modelling process) and is assigned a proportional number of draws that contribute to the final model. Sub-models are selected from mixed-effects linear models and spatio-temporal Gaussian process regression using either the percent of deaths that are due to LRI or the LRI death rate.

Covariates are selected independently for each sub-model and the selection is based on an algorithm that captures biologically plausible relationships between the covariates and LRI mortality and provides a diversity of possible models. A list of covariates that the models select from is provided in **Table 1**. For every covariate, the direction of effect and a level of biologic proximity to LRI mortality was defined by the modeler. Each model includes all combinations of covariates if the direction of effect is along the assumed direction and the coefficient is significant at the $p < 0{\cdot}05$ level. Also, if adding a higher level covariate changes the significance of a level one to non-significant or an implausible direction, it will be dropped from the set. The reason for this algorithm is to give priority and emphasis on covariates that are more causally and proximately related to LRI such as air pollution and malnutrition rather than more contextual and macro covariates such as education and income per capita.

LRI mortality is estimated for 21 age groups, 591 locations, both sexes, and every year from 1980-2015. We estimated LRI mortality separately for males and females and for children under 5 years and older than 5 years. Data-rich and data-poor geographic locations were modeled separately and these models were then hybridized for a final global model for each sex.

LRI mortality estimates are then squeezed into an overall mortality envelope by age/sex/location/year in a process called CoDCorrect. This step is to ensure internal consistency among causes of death and that the sum of cause-specific mortality is the same as the estimated all-cause mortality.

Figure 1. LRI mortality data geographic distribution. The number of verbal autopsy or vital registration data points for all ages and from 1980-2015 are shown. Countries in white have no data. Subnational estimation occurs in the United States, Mexico, Brazil, South Africa, the United Kingdom, Saudi Arabia, India, China, and Japan. Input data and models can be found using the GBD visualization tools at: http://vizhub.healthdata.org/data-visualizations.

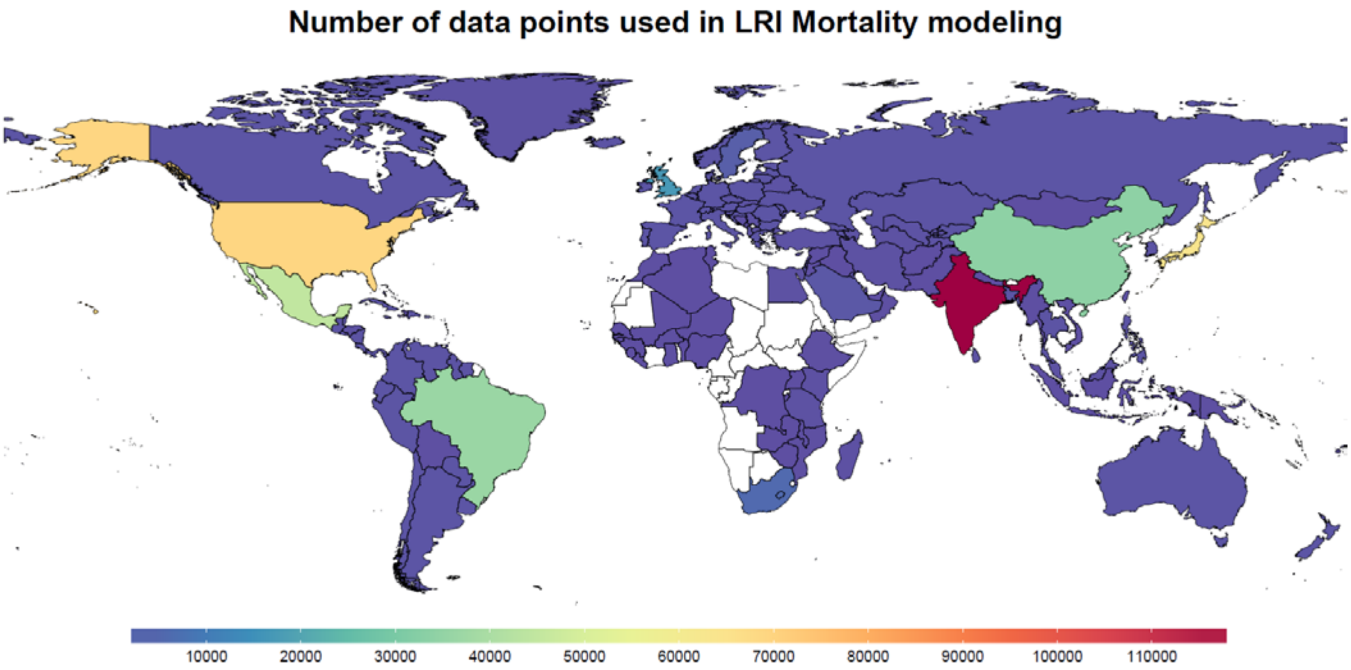

Table 1. Covariates in CODEm. CODEm uses a covariate selection algorithm and chooses from the covariates listed in the table below. Covariates are selected from this list while considering prior information about the strength of the association and direction of effect between the covariate and LRI mortality. The strength of the relationship is ranked from 1 (in causal pathway) to 3 (likely related to LRI mortality).

Covariate	Strength of Relationship	Direction
LRI Summary Exposure Variable	1	+
Malnutrition <2 SD	1	+
Hib vaccine coverage	1	-
Pneumococcal conjugate vaccine coverage	1	-
Indoor air pollution	1	+
Smoking prevalence	1	+
DTP3 vaccine coverage	2	-
Education per Capita	3	-
LDI per Capita	3	-
Outdoor air pollution (PM2·5)	3	+
Water and sanitation SEV	3	+
Socio-demographic status	3	-

Summary of LRI morbidity modelling

LRI are characterized as clinician-diagnosed or x-ray confirmed pneumonia or bronchiolitis. Cases are split into moderate and severe/very severe episodes using a severity definition that closely matches the WHO Integrated Management of Childhood Illness categories of pneumonia (**Table 2**).[4]

The distribution of moderate (84·9%, 95% UI: 83·1-86·7%) and severe/very severe (15·1%, 95% UI: 13·3-16·9%) LRI is determined by a random effects meta-analysis of the ratio of severe to all LRI from 15 studies that report the incidence of moderate and severe LRI. Years lived with disability (YLDs) are calculated using the severity distribution of LRI cases and the disability weights associated with them. The disability weights were separately estimated by the Disability Weights Survey portion of the GBD study and were systematically constructed based on responses from more than 6,000 survey respondents.[5]

The non-fatal LRI burden, including incidence and prevalence, is modeled in DisMod-MR 2·1 (DisMod). DisMod is a Bayesian, hierarchical, age-integrating meta-regression tool that relates incidence, prevalence, recovery, and mortality. Input data are from a systematic literature review of cross-sectional and cohort studies, hospital inpatient and outpatient data (ICD9 Codes included 073·0-073·6, 079·82, 466-469, 480-489, 513·0, and 770·0. ICD10 codes included A48·1, J09-J22, J85·1, P23-P23·9, and U04), MarketScan healthcare utilization data (USA only), and population-representative surveys. To make the data more consistent for covariates and adjustments in the modelling process, we converted all incidence data to prevalence data using an average duration of illness of ten days. Input data include all data used in GBD 2013 and a new review of data sources from January 2012-August 2015 (**Table 3, Figure 2**).

Input data are adjusted to our standard case definition. Data are adjusted by study-level binary covariates which describe if the source is a hospital or inpatient sample and if the data come from a self-reported survey (**Table 4**). Self-reported prevalence of LRI symptoms from population-representative surveys such as the Demographic and Health Survey (DHS) and the Multiple Indicator Cluster Survey (MICS) is used. Our case definition from symptom-based prevalence estimates is children in the last two weeks with fever and cough with difficulty breathing and symptoms located in the chest and/or chest and nose. This is consistent with the WHO Integrated Management of Childhood Illness guidelines definition of pneumonia and with the DHS and MICS definition of acute respiratory infection.[4] We extracted the prevalence of children under 5 years old that had fever and cough with difficulty breathing and included an indicator for this less-specific definition.

Some surveys did not include the prevalence of LRI symptoms *and* fever so we adjusted survey prevalence estimates that did not include fever by studies that did based on a logistic regression. These data were converted from two-week period prevalence to point prevalence using a mean duration of illness of ten days. The illness duration was based on expert opinion as data on the duration of untreated LRI were sparse. Where applicable, period prevalence was converted to point prevalence using the following formula:

$$Prevalence_{Point} = Prevalence_{Period} * \frac{Duration}{Duration - 1 + Recall\ period}$$

Country-level covariates also inform the model. These include Hib and pneumococcal conjugate vaccine coverage, health system access, income per capita, and the SEV for LRI (**Table 4**). In addition, the model is informed by cause-specific mortality estimates from a different process for mortality and cause of death in GBD 2015. PCV and Hib vaccine coverage are modeled as covariates for GBD 2015. The values are modeled using spatio-temporal Gaussian Process Regression, a Bayesian modeling approach to leverage space-time variation, using input data from surveys, delivery campaigns, and information about national vaccination infrastructure.[6]

Figure 2. Geographic distribution of LRI morbidity modelling data. The number of data points for all ages and from 1990-2015 are shown. Countries in white have no data. Subnational estimation occurs in the United States, Mexico, Brazil, South Africa, the United Kingdom, Saudi Arabia, India, China, and Japan. Input data and models can be found using the GBD visualization tools at: http://vizhub.healthdata.org/data-visualizations.

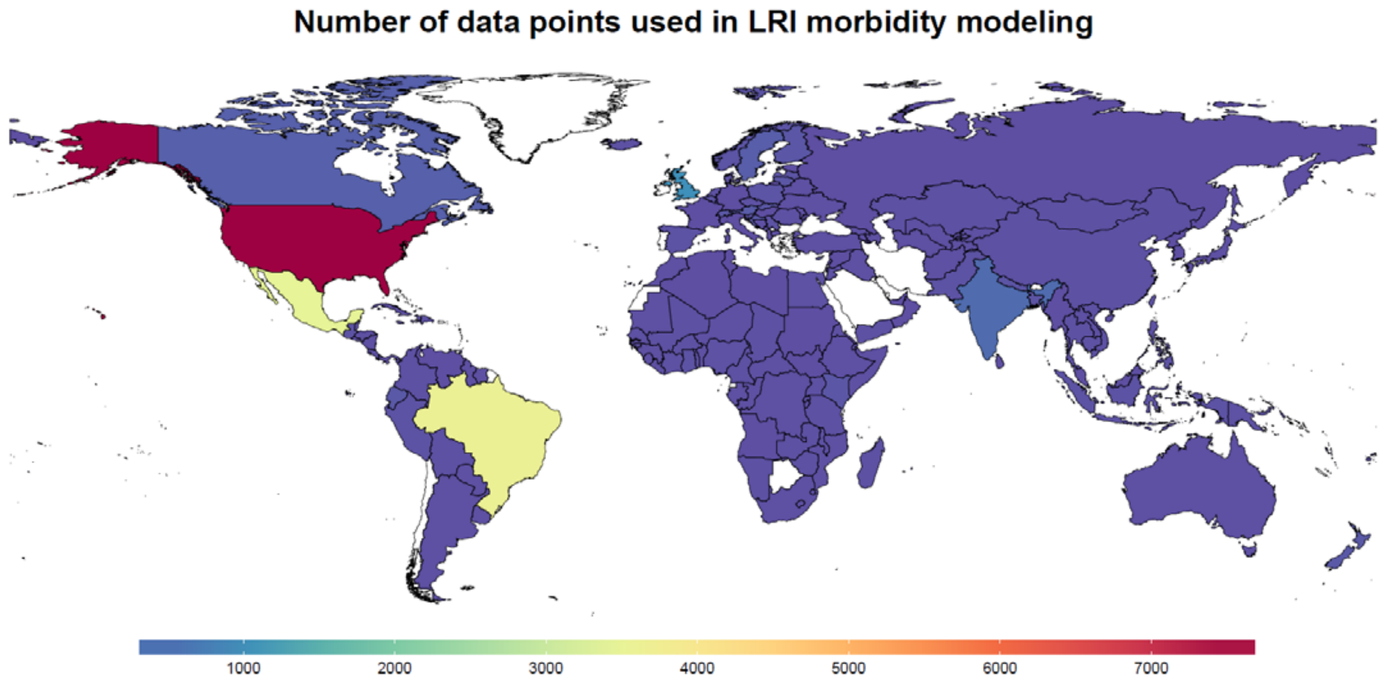

Table 2. Severity definitions for moderate and severe/very severe LRI.

Severity level	Lay description	Disability Weight (95% CI)	Percent of episodes (95% CI)
Moderate	Cough or difficulty breathing with rapid breathing<br>Has a fever and aches and feels weak which causes some difficulty with daily activities	0·051<br>(0·032-0·074)	84·9%<br>(83·1-86·7%)
Severe/very severe	Cough or difficulty breathing with lower chest wall indrawing, central cyanosis, or the inability to drink<br>Has a high fever and pain and feels very weak, which causes great difficulty with daily activities	0·133<br>(0·088-0·19)	15·1%<br>(13·3-16·9%)

Table 3. Data Inputs for LRI modeling. Mortality risk data are from GBD 2015 Cause of Death estimates.

	Prevalence	Mortality risk
Sources	585	9 non-IHME
Data points	20,506	18,333
Countries/subnationals	377	218
GBD world regions	21	20

Table 4. Covariates in the LRI DisMod model.

Study covariate	Parameter	Exponentiated beta (95% UI)
Hospital inpatient population	Prevalence	0·44 (0·42-0·46)
Hospital data from middle- or low-income country	Prevalence	0·29 (0·24-0·32)
Self-reported	Prevalence	1·08 (1·02-1·15)
Poor diagnostic specificity	Prevalence	1·34 (1·25 -1·45)
Hib vaccine coverage	Prevalence	0·72 (0·67-0·77)
PCV vaccine coverage	Prevalence	0·87 (0·84-0·90)
LRI SEV	Prevalence	0·78 (0·69-0·86)
Sociodemographic status	Excess mortality	0·55 (0·49-0·65)

Summary of etiology population attributable fraction strategy

We estimated LRI etiologies separately from overall LRI mortality using two distinct counterfactual modelling strategies based on population attributable fractions (PAFs), described in detail below. We did not attribute etiologies to neonatal pneumonia due to a dearth of reliable data in this age group. We calculated uncertainty of our PAF estimates from 1,000 draws of each parameter using normal distributions in log space.

Pneumococcal pneumonia and Hib

For *Streptococcus pneumoniae* (pneumococcal pneumonia) and *Haemophilus influenzae* type B (Hib), we calculated the population attributable fraction using a vaccine probe design.[7,8] The ratio of vaccine effectiveness against nonspecific pneumonia to pathogen-specific disease represents the fraction of pneumonia cases attributable to each pathogen.

To estimate the PAF for Hib and pneumococcal pneumonia, we calculated the ratio of vaccine effectiveness against nonspecific pneumonia to pathogen-specific pneumonia (Equations 1 and 3). We estimated a study-level estimate of PAF from a meta-analysis of these ratios (**Table 6**). To estimate the PAF for Hib, we only used randomized controlled trials because of implausibly high values of vaccine efficacy in case-control studies. To estimate the PAF for pneumococcal pneumonia, we included RCTs and before and after vaccine introduction longitudinal studies.

We adjusted the study-level PAF estimate by vaccine coverage and expected vaccine performance to estimate country- and year-specific PAF values. For pneumococcal pneumonia, we adjusted the PAF by the final Hib PAF estimate and by vaccine serotype coverage.[9] Finally, we used an age distribution of PAF modeled in DisMod to determine the PAF by age. Because of an absence of data describing vaccine efficacy against Hib in children older than 2 years of age, we did not attribute Hib to episodes of LRI in ages 5 years and older.

We used a vaccine probe design to estimate the PAF for pneumococcal pneumonia and (Hib) by first calculating the ratio of vaccine effectiveness against nonspecific pneumonia to pathogen-specific pneumonia at the study level (Equations 1 and 2).[7,8,10] We then adjusted this estimate by vaccine coverage and expected vaccine performance to estimate country- and year-specific PAF values (Equations 3 and 4).

$$1)\quad HibPAF_{Base} = 1 - \frac{VE_{Pneumonia}}{VE_{Hib}}$$

$$2)\quad PneumoPAF_{Base} = 1 - \frac{VE_{Pneumonia} * (1 - PAF_{Hib} * VE_{Hib\ Optimal})}{VE_{Streptococcus} * Cov_{Serotype}}$$

$$3)\quad PAF_{Hib} = PAF_{Base} * \frac{(1 - Cov_{Hib} * VE_{Hib\ Optimal})}{(1 - PAF_{Base} * Cov_{Hib} * VE_{Hib\ Optimal})}$$

$$4)\quad PAF_{Pneumo} = \frac{PAF_{Base} * \left(1 - Cov_{PCV} * VE_{PCV\ Optimal}\right)}{\left(1 - PAF_{Hib} * Cov_{Hib} * VE_{Hib\ Optimal}\right) * \left(1 - \frac{PAF_{Base} Cov_{PCV} * VE_{PCV\ Optimal}}{\left(1 - PAF_{Hib} * Cov_{Hib} * VE_{Hib\ Optimal}\right)}\right)}$$

Where $VE_{Pneumonia}$ is the vaccine efficacy against nonspecific pneumonia, VE_{Hib} is the vaccine efficacy against invasive Hib disease, $VE_{Streptococcus}$ is the vaccine efficacy against serotype-specific pneumococcal pneumonia, $Cov_{serotype}$ is the serotype-specific vaccine coverage for PCV,[9] $VE_{Hib\ Optimal}$ is the Hib effectiveness in the community (0·8)[11], PAF_{Hib} is the final PAF for Hib, Cov_{PCV} is the PCV coverage, Cov_{Hib} is the Hib coverage by country, and $VE_{PCV\ Optimal}$ is the vaccine effectiveness in the community (0·8).[12] **Figure 4** shows the global maps for PCV and Hib vaccine coverage estimates among all ages in 2005 and 2015.

For Hib, we assumed that the vaccine efficacy against invasive Hib disease is the same against Hib pneumonia. For pneumococcal pneumonia, a recent study in adults [13] found that the vaccine efficacy against invasive pneumococcal disease may be significantly higher than against pneumococcal pneumonia. We used this ratio to adjust estimates of vaccine efficacy against invasive pneumococcal disease from other studies. However, recognizing that the study is unique in that it uses a urine antigen test among adults, we added uncertainty around our adjustment using a wide uniform distribution (median 0·65, 0·3-1·0). This has increased the estimates of pneumococcal pneumonia mortality in a meaningful way.

Influenza and RSV

The population attributable fractions (PAFs) for influenza and respiratory syncytial virus (RSV) are estimated using the following formula:[14]

$$5) \quad PAF = Proportion * (1 - \frac{1}{OR})$$

Where *Proportion* is the proportion of LRI cases that test positive for influenza or RSV and *OR* is the odds ratio of LRI given the presence of the pathogen. We used an odds ratio of 5·1 (3·19 – 8·14) for influenza and 9·79 (4·98 – 19·27) for RSV from a recently published meta-analysis.[15] These odds ratios are marginally different from those used in GBD 2013.

The *Proportion* values are modeled estimates. We used the meta-regression tool DisMod-MR to estimate the proportion of LRI cases that are positive for influenza and RSV, separately, by location/year/age/sex. These models are informed by data from a systematic review of cohort, cross-sectional, and clinical trial studies (**Search string 2, Table 5**). Our inclusion criteria were studies that were published between January 1990 and June 2015, a sample size of at least 100, at least one year in duration, and with LRI, pneumonia, or bronchiolitis as the case definition. We excluded studies that described pandemic H1N1 influenza solely and studies that used influenza-like illness as the case definition. If the ages of the study participants were not reported, we assigned an age range based on the prevalence-weighted mean age of LRI, from the LRI prevalence DisMod model, for the appropriate year/sex/location.

There are separate PAF values for nonfatal and fatal LRI episodes. Fatal PAFs are adjusted using a scalar from the DisMod proportion models that represents the relative frequency of detection in inpatient versus non-inpatient sample populations. The value of these scalars are 0·92 (95% UI: 0·7-1·22) and 0·71 (95% UI: 0·52-0·94) for influenza and RSV, respectively. In addition, as the case-fatality of viral causes of pneumonia is lower than for bacterial causes, we adjusted the fatal PAF estimates by determining the ratio of case-fatality among viral to bacterial causes of pneumonia from hospital data coded specifically to these causes. Hospital data were limited to the USA, Austria, Brazil, and Mexico. We generated an age-specific ratio of case-fatality between viral and bacterial causes of LRI using DisMod-MR (**Table 7**).

Information on the sources for aetiology modeling in GBD 2015 can be found on the Global Health Data Exchange (http://internal-ghdx.healthdata.org/gbd-2015).

Search Strings

1. ('lower respiratory'[title/abstract] OR pneumonia[title/abstract] AND ('2012/01/01'[PDat] : '2015/12/31'[PDat])AND Humans[MeSH Terms] NOT(autoimmune[title/abstract] OR COPD [title/abstract] OR 'cystic fibrosis'[title/abstract])
2. (“lower respiratory”[title/abstract] OR pneumonia[title/abstract] AND (influenza[title/abstract] OR influenza[MeSH Terms] OR “respiratory syncytial”[title/abstract] OR etiolog*[title/abstract]) AND ("2012/01/01"[PDat] : "2015/12/31"[PDat])AND Humans[MeSH Terms]
3. (“haemophilus influenzae type b” OR “haemophilus influenzae b”) AND vaccine AND (efficacy OR effectiveness) AND ("2012/01/01"[PDat] : "2015/12/31"[PDat])AND Humans[MeSH Terms] NOT(autoimmune[title/abstract] OR COPD [title/abstract] OR "cystic fibrosis"[title/abstract])
4. ('streptococcus pneumoniae' OR pneumococcus OR pneumococcal) AND ('conjugate vaccine' OR 'polysaccharide vaccine') AND (efficacy OR effectiveness) AND ('2012/01/01'[PDat] : '2015/12/31'[PDat])AND Humans[MeSH Terms]

Table 5. Summary of LRI etiology data used in attributable fraction estimation. The number of data points does not necessarily indicate the number of unique sources used in the modeling. Data in the modeling are included at the most detailed possible level based on age, year, sex, and geography. More information including meta-data on the sources used in the aetiology modeling can be found on the Global Health Data Exchange: http://ghdx.healthdata.org/gbd-2015

Etiology	Total data points	Data points new to GBD 2015	Number (percent) of GBD Locations	Number (percent) from Inpatient population	Number (percent) from Children under 5yrs
Respiratory syncytial virus	368	277 (75·3%)	84 (15%)	48 (13%)	191 (51·9%)
Influenza	482	404 (83·8%)	92 (16·4%)	57 (11·8%)	153 (31·7%)
Pneumococcal pneumonia	30	2 (6·7%)	8 (1·4%)	NA	15 (50%)
Hib pneumonia	4	0 (0%)	4 (<1%)	NA	4 (100%)

Table 6. Sources in the Hib and *Streptococcus pneumoniae* vaccine efficacy meta-analysis.

Citation	Year	Location
**Pneumococcal pneumonia**		
Ansaldi F, Sticchi L, Durando P, Carloni R, Oreste P, Vercelli M, Crovari P, Icardi G. Decline in pneumonia and acute otitis media after the introduction of childhood pneumococcal vaccination in Liguria, Italy. *J Int Med Res*. 2008; 36(6): 1255-60.	2008	Italy
Bonten MJM, Huijts SM, Bolkenbaas M, Webber C, Patterson S, Gault S, van Werkhoven CH, van Deursen AMM, Sanders EAM, Verheij TJM, Patton M, McDonough A, Moradoghli-Haftvani A, Smith H, Mellelieu T, Pride MW, Crowther G, Schmoele-Thoma B, Scott DA, Jansen KU, Lobatto R, Oosterman B, Visser N, Caspers E, Smorenburg A, Emini EA, Gruber WC, Grobbee DE. Polysaccharide conjugate vaccine against pneumococcal pneumonia in adults. *N Engl J Med*. 2015; 372(12): 1114–25	2015	Netherlands
Cutts F, Zaman SM, Enwere G, Jaffar S, Levine O, Okoko J, Oluwalana C, Vaughan A, Obaro S, Leach A, McAdam K, Biney E, Saaka M, Onwuchekwa U, Yallop F, Pierce N, Greenwood B, Adegbola R. Efficacy of nine-valent pneumococcal conjugate vaccine against pneumonia and invasive pneumococcal disease in The Gambia: randomised, double-blind, placebo-controlled trial. *Lancet*. 2005; 365(9465): 1139-46	2005	The Gambia
Grijalva CG, Nuorti JP, Arbogast PG, Martin SW, Edwards KM, Griffin MR. Decline in pneumonia admissions after routine childhood immunisation with pneumococcal conjugate vaccine in the USA: a time-series analysis. *Lancet*. 2007; 369(9568): 1179-86	2007	United States
Hansen J, Black S, Shinefield H, Cherian T, Benson J, Fireman B, Lewis E, Ray P, Lee J. Effectiveness of Heptavalent Pneumococcal Conjugate Vaccine in Children Younger Than 5 Years of Age for Prevention of Pneumonia: Updated Analysis Using World Health Organization Standardized Interpretation of Chest Radiographs. *Pediatr Infect Dis J*. 2006; 25(9): 779-81	2006	United States
Jardine A, Menzies RI, McIntyre PB. Reduction in hospitalizations for pneumonia associated with the introduction of a pneumococcal conjugate vaccination schedule without a booster dose in Australia. *Pediatr Infect Dis J*. 2010; 29(7): 607-12.	2010	Australia
Klugman KP, Madhi SA, Huebner RE, Kohberger R, Mbelle N, Pierce N, Vaccine Trialists Group. A trial of a 9-valent pneumococcal conjugate vaccine in children with and those without HIV infection. *N Engl J Med*. 2003; 349(14): 1341-8	2003	South Africa
Lucero MG, Tallo V, Lupisan S, Sanvictores D, Ugpo J, Lechago M, Abucejo-Ladesma E, Sombrero L, Nohynek H, Puumalainen T, Nissinen A, Soininen A, Ruutu P, Makela HP, Williams G, Forsyth S, De Campo M, Riley I, Simoes EAF. Efficacy of an 11-valent pneumococcal conjugate vaccine against radiologically confirmed pneumonia among children less than 2 years of age in the Philippines: A randomized, double-blind, placebo-controlled trial. *Pediatr Infect Dis J*. 2009; 28(6): 455-62	2009	Philippines
Martinelli D, Pedalino B, Cappelli MG, Caputi G, Sallustio A, Fortunato F, Tafuri S, Cozza V, Germinario C, Chironna M, Prato R. Towards the 13-valent pneumococcal conjugate universal vaccination: effectiveness in the transition era between PCV7 and PCV13 in Italy, 2010-2013. *Hum Vaccin Immunother*. 2014; 10(1): 33-9	2014	Italy
Simonsen L, Taylor RJ, Young-Xu Y, Haber M, May L, Klugman KP. Impact of Pneumococcal Conjugate Vaccination of Infants on Pneumonia and Influenza Hospitalization and Mortality in All Age Groups in the United States. *MBio*. 2011; 2(1): e00309-10	2011	United States
Tregnaghi MW, Sáez-Llorens X, López P, Abate H, Smith E, Pósleman A, et al. Evaluating the efficacy of 10-valent pneumococcal non-typeable Haemophilus influenzae protein-D conjugate vaccine (PHiD-CV) against community-acquired pneumonia in Latin America [abstract]. In: Abstracts of the 29th Annual Meeting of the European Society for Paediatric Infectious Diseases (ESPID); 2011 June 7-11; The Hague, The Netherlands	2011	Panama
Zhou F, Kyaw MH, Shefer A, Winston CA, Nuorti J. Health care utilization for pneumonia in young children after routine pneumococcal conjugate vaccine use in the United States. *Arch Pediatr Adolesc Med*. 2007; 161(12): 1162-8	2007	United States
**Hib**		
Baqui AH, El Arifeen S, Saha SK, Persson L, Zaman K, Gessner BD, Moulton LH, Black RE, Santosham M. Effectiveness of Haemophilus influenzae type B conjugate vaccine on prevention of pneumonia and meningitis in Bangladeshi children: a case-control study. *Pediatr Infect Dis J*. 2007; 26(7): 565-71	2007	Bangladesh
Gessner BD, Sutanto A, Linehan M, Djelantik IG, Fletcher T, Gerudug IK, Ingerani, Mercer D, Moniaga V, Moulton LH, Moulton LH, Mulholland K, Nelson C, Soemohardjo S, Steinhoff M, Widjaya A, Stoeckel P, Maynard J, Arjoso S. Incidences of vaccine-preventable Haemophilus	2005	Indonesia

influenzae type b pneumonia and meningitis in Indonesian children: hamlet-randomised vaccine-probe trial. *Lancet*. 2005; 365(9453): 43-52		
Levine OS, Lagos R, Muñoz A, Villaroel J, Alvarez AM, Abrego P, Levine MM. Defining the burden of pneumonia in children preventable by vaccination against Haemophilus influenzae type b. *Pediatr Infect Dis J*. 1999; 18(12): 1060-4	1999	Chile
Mulholland K, Hilton S, Adegbola R, Usen S, Oparaugo A, Omosigho C, Weber M, Palmer A, Schneider G, Jobe K, Lahai G, Jaffar S, Secka O, Lin K, Ethevenaux C, Greenwood B. Randomised trial of Haemophilus influenzae type-b tetanus protein conjugate vaccine corrected for prevention of pneumonia and meningitis in Gambian infants. *Lancet*. 1997; 349(9060): 1191-7	1997	The Gambia

Table 7. The median values for the ratio of case fatality for viral to bacterial pneumonia. These estimates are modeled using hospital-based, ICD-coded admissions and mortality for viral and bacterial pneumonia. Values in parentheses represent the 95% uncertainty interval.

Age Group	Ratio
Early Neonatal	0·34 (0·19-0·58)
Late Neonatal	0·34 (0·19-0·58)
Post Neonatal	0·34 (0·19-0·58)
1 to 4	0·28 (0·16-0·44)
5 to 9	0·31 (0·15-0·56)
10 to 14	0·33 (0·19-0·53)
15 to 19	0·37 (0·2-0·64)
20 to 24	0·46 (0·12-1·16)
25 to 29	0·44 (0·17-0·93)
30 to 34	0·46 (0·22-0·83)
35 to 39	0·5 (0·22-1)
40 to 44	0·61 (0·13-1·75)
45 to 49	0·5 (0·21-0·99)
50 to 54	0·44 (0·23-0·74)
55 to 59	0·42 (0·21-0·75)
60 to 64	0·42 (0·15-0·95)
65 to 69	0·39 (0·19-0·7)
70 to 74	0·38 (0·21-0·61)
75 to 79	0·37 (0·2-0·62)
80 plus	0·37 (0·17-0·71)

Figure 3. Geographic distribution of etiology data. A) Number of data points for *Streptococcus pneumoniae* (30), **B)** Number of data points for Hib (4), **C)** Number of data points for influenza (482), **D)** Number of data points for respiratory syncytial virus (368).

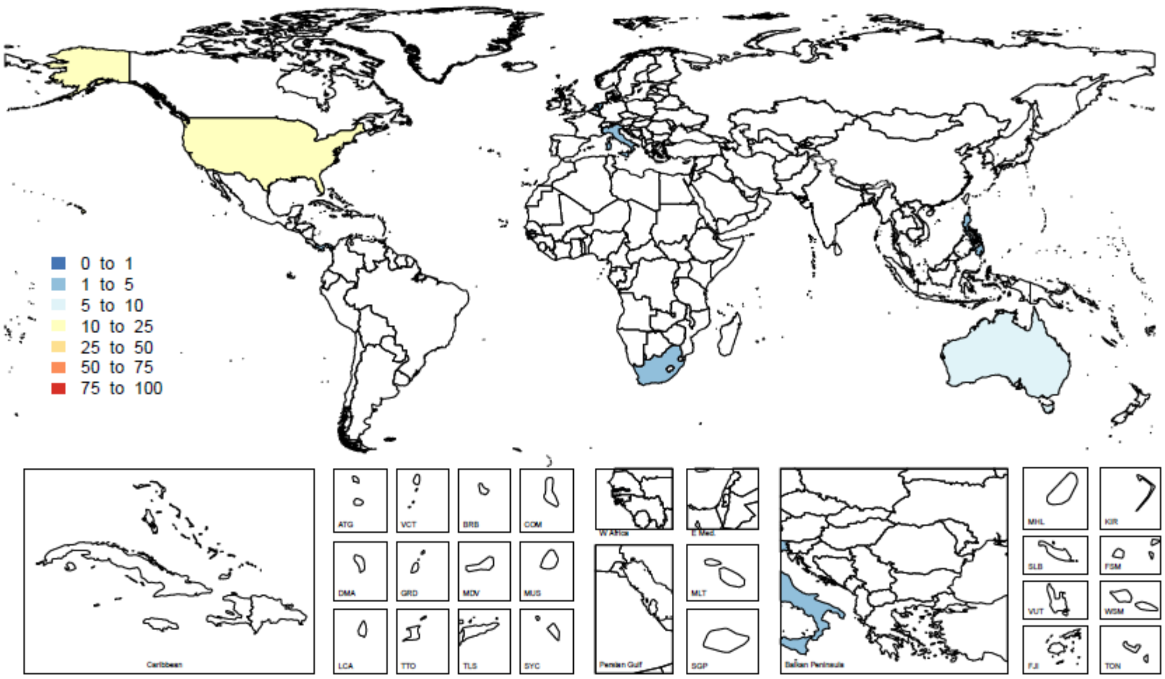

A)

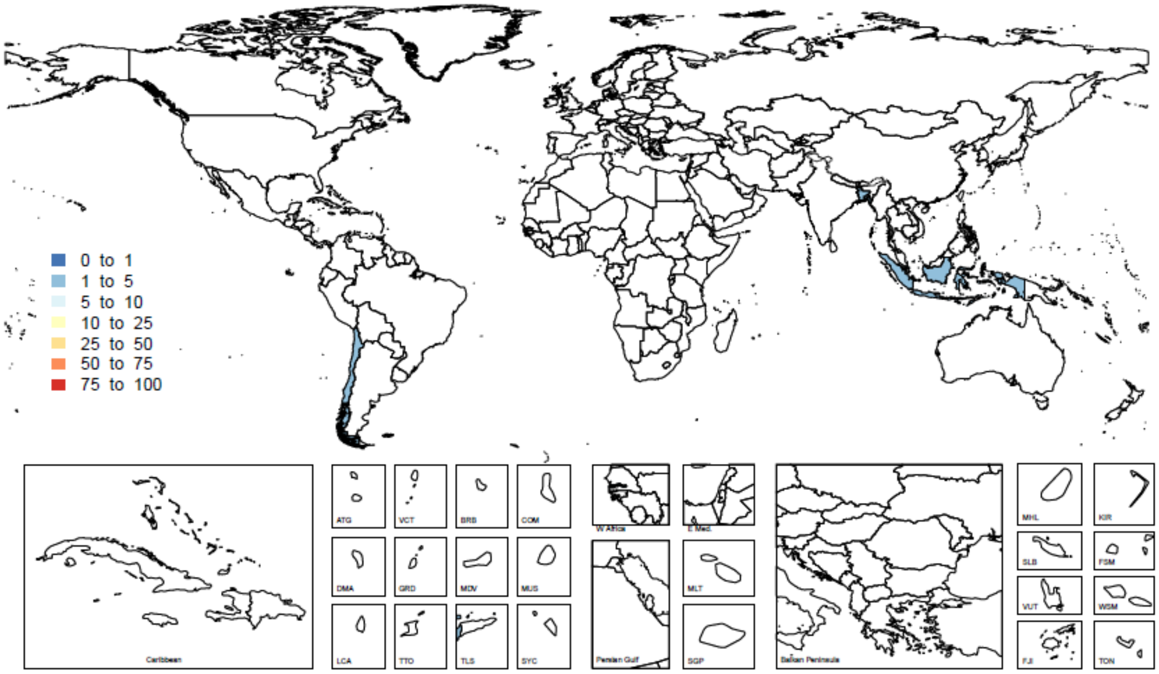

B)

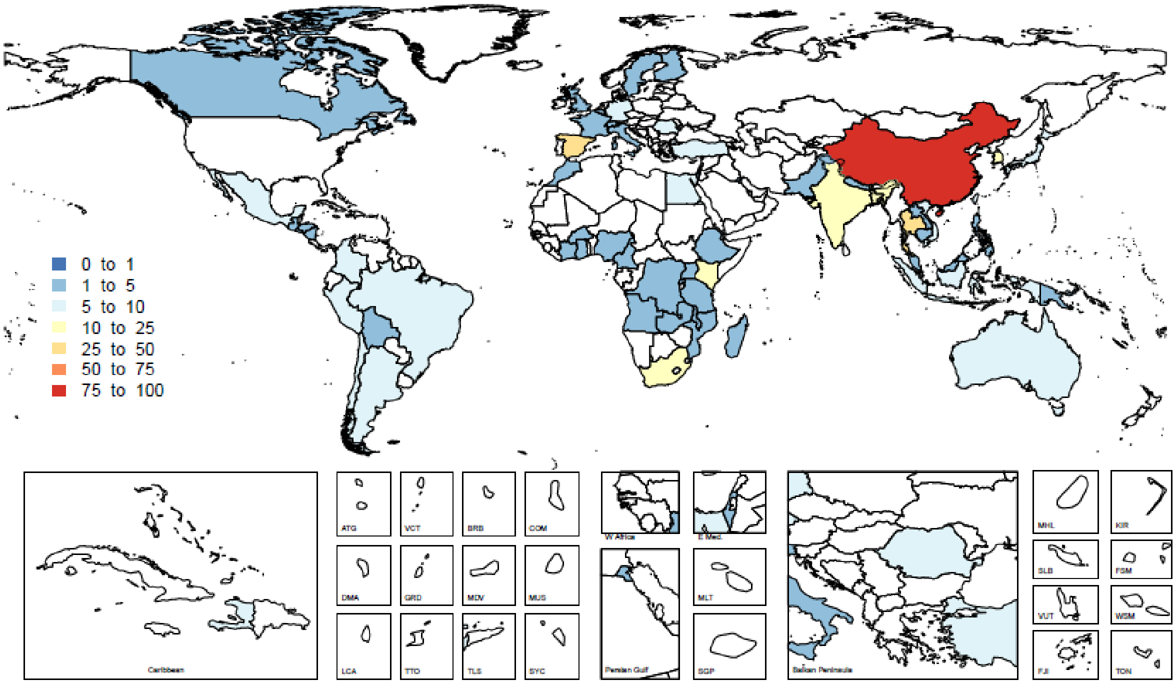
0 to 1
1 to 5
5 to 10
10 to 25
25 to 50
50 to 75
75 to 100
Caribbean
ATG
VCT
BRB
COM
DMA
GRD
MDV
MUS
LCA
TTO
TLS
SYC
W Africa
E Med.
Persian Gulf
MLT
SGP
Balkan Peninsula
MHL
KIR
SLB
FSM
VUT
WSM
FJI
TON

C)

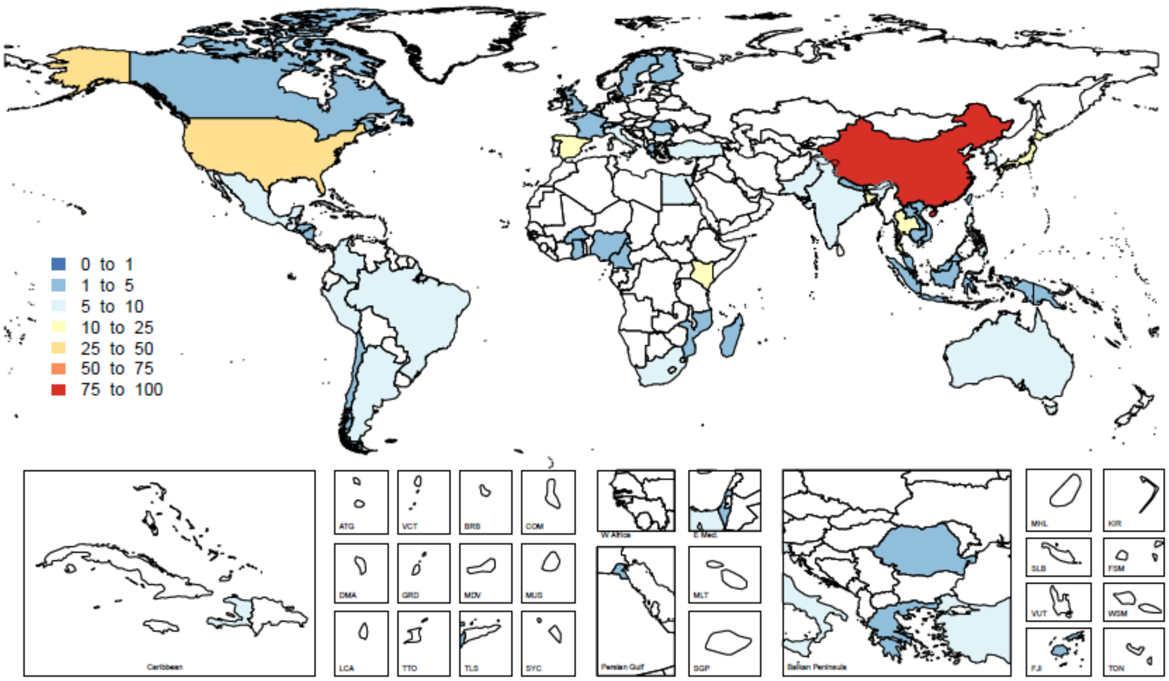
0 to 1
1 to 5
5 to 10
10 to 25
25 to 50
50 to 75
75 to 100
Caribbean
ATG
VCT
BRB
COM
DMA
GRD
MDV
MUS
LCA
TTO
TLS
SYC
W Africa
E Med.
Persian Gulf
MLT
SGP
Balkan Peninsula
MHL
KIR
SLB
FSM
VUT
WSM
FJI
TON

D)

Figure 4. Vaccine coverage estimates for all ages. Vaccine coverage estimates are modeled as part of the covariate models for GBD 2015. **A)** Hib coverage in 2005, **B)** Hib coverage in 2015, **C)** PCV coverage in 2005, & **D)** PCV coverage in 2015.

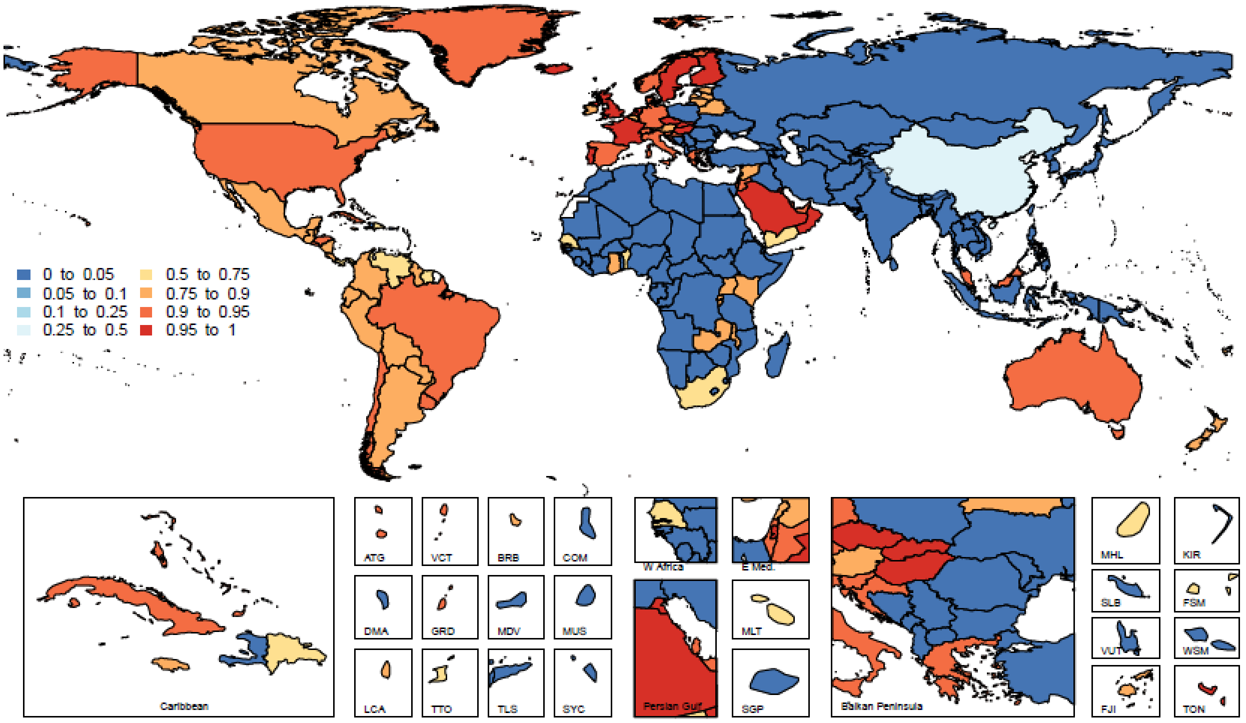

A)

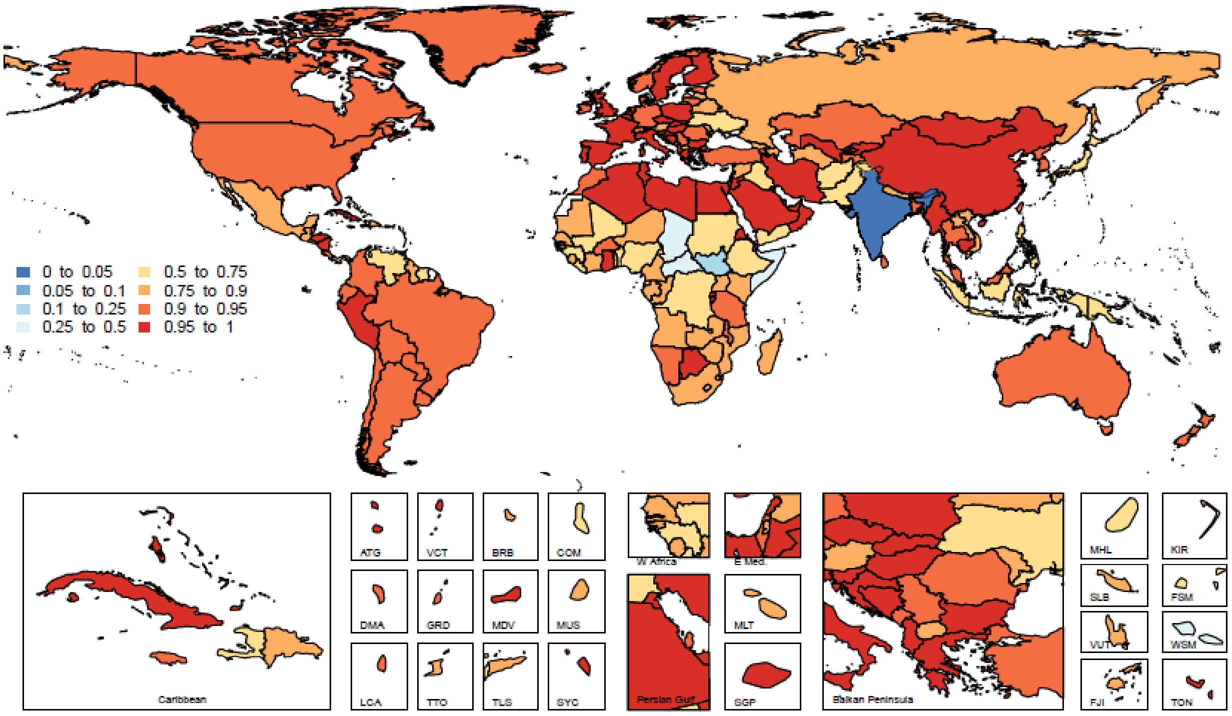

B)

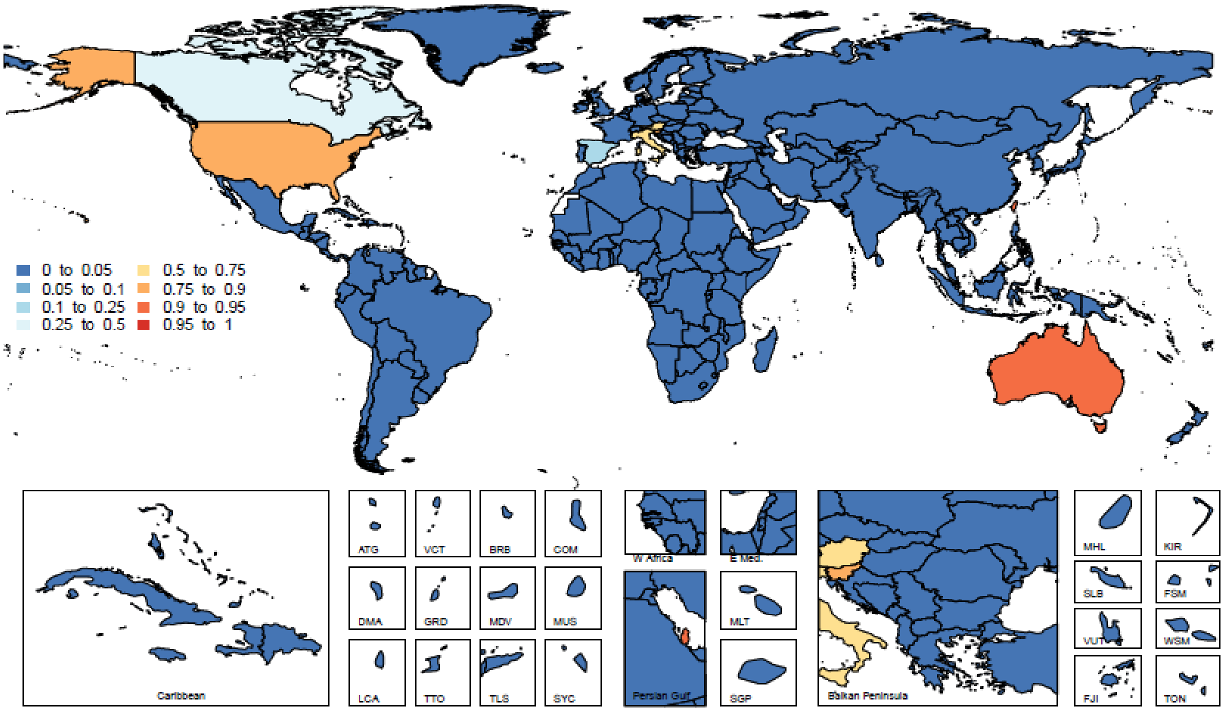
0 to 0.05
0.05 to 0.1
0.1 to 0.25
0.25 to 0.5
0.5 to 0.75
0.75 to 0.9
0.9 to 0.95
0.95 to 1
Caribbean
ATG
VCT
BRB
COM
DMA
GRD
MDV
MUS
LCA
TTO
TLS
SYC
W Africa
E Med.
Persian Gulf
MLT
SGP
Balkan Peninsula
MHL
KIR
SLB
FSM
VUT
WSM
FJI
TON

C)

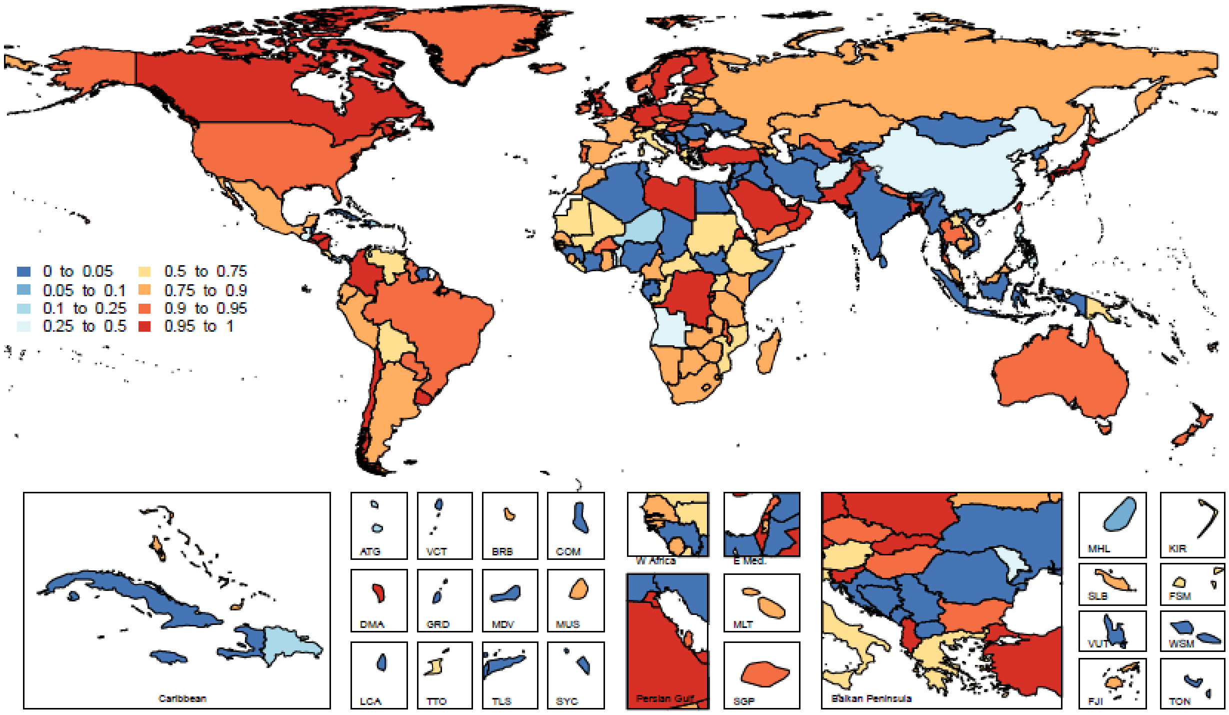
0 to 0.05
0.05 to 0.1
0.1 to 0.25
0.25 to 0.5
0.5 to 0.75
0.75 to 0.9
0.9 to 0.95
0.95 to 1
Caribbean
ATG
VCT
BRB
COM
DMA
GRD
MDV
MUS
LCA
TTO
TLS
SYC
W Africa
E Med.
Persian Gulf
MLT
SGP
Balkan Peninsula
MHL
KIR
SLB
FSM
VUT
WSM
FJI
TON

D)

Comparison to GBD 2013

The differences in final estimates for LRI mortality and morbidity between GBD 2013 and GBD 2015 are shown in **Figure 5.** One major change in estimation between GBD rounds is the introduction of subnational estimation in India and China. Modelling LRI burden at the subnational level increases the number of input data points and improves resolution by allowing for greater within-country variation. The number of LRI deaths in children under 5 in 2010 is much lower in GBD 2015 (886,479, 95% UI: 826,641-949,912) compared to GBD 2013 (984,888, 95% UI: 892,595-1,075,280). Much of this difference is attributable to three high-burden countries, Nigeria, Pakistan, and the Democratic Republic of the Congo, and can be traced to changes in input data values or availability and not changes in modelling strategy. The number of children under-5 deaths in Nigeria and the DRC deviates between GBD versions are due mainly to changes in the availability of data. Of two data points in Nigeria among under-5 age groups, one data point was notably different in the number of deaths attributed to LRI in GBD version and three verbal autopsy studies from the DRC were included in GBD 2013 but not in GBD 2015. In contrast, the number of deaths due to LRI from two verbal autopsy data points in Pakistan were much lower in GBD 2015 compared to GBD 2013. These updates in Cause of Death data are especially pronounced in locations with sparse data (**Figure 1**).

The etiologic fractions of LRI mortality among children under 5 in 2010 are shown in **Figure 6.** The attributable fraction due to pneumococcal pneumonia has increased substantially from GBD 2013 to GBD 2015 while influenza, RSV, and Hib are similar between GBD rounds. The fraction of LRI deaths that are unattributed to any pathogen has decreased from 50% to 25%.

Our estimation of the LRI deaths attributable to pneumococcal pneumonia relies on data from vaccine efficacy studies that show a decrease in all pneumonia and among invasive (bacteraemic) pneumococcal disease in children and adults. However, at least one study using a urine antigen test in elderly adults found that the relative vaccine efficacy of pneumococcal conjugate vaccine (PCV) against invasive pneumococcal disease was 66% greater than against pneumococcal pneumonia.[16] For GBD 2015 we corrected the estimated PCV efficacy against invasive pneumococcal disease by this ratio in both child and adult age groups. This is a change in our GBD 2015 methodology that was not used in GBD 2013, although no studies have used this diagnostic test to detect non-invasive pneumococcal pneumonia in children due to high carriage of *Streptococcus pneumoniae* in healthy children.[17–19] To reflect the uncertainty of this adjustment, we used a uniform distribution around the point estimate of the ratio. This adjustment contributed to an increase in our estimates of pneumococcal pneumonia mortality as the vaccine efficacy against pneumococcal pneumonia may be lower than previously estimated.[16] Given the much larger attributable fraction and wide uncertainty of the final estimates, further studies to confirm and precisely quantify the difference in vaccine efficacy between invasive and non-invasive disease are needed.

The attributable fraction for Hib was not statistically significant among children under 5 at the global level. Indeed, this is biologically implausible as these pathogens do not have a protective effect against mortality. The attributable fraction for Hib is based on a meta-analysis of randomised controlled trials of vaccine efficacy where the confidence interval of the pooled estimate was not statistically significant. This is a potential limitation and future analyses could use alternative meta-analytic methods, such as log-log meta-analysis or imposing a non-negative population attributable fraction prior, to prevent negative population attributable fraction estimates. Data availability limitations, particularly for vaccine efficacy data across age groups, hindered our ability to conduct pathogen attribution analyses for Hib among populations 5 years and older; a similar lack of data made estimating population attributable fractions and pathogen-specific mortality for neonates analytically infeasible.

Figure 5. Scatterplot of GBD 2015 and GBD 2013 results by country in 1990 and 2010.[20,21] **A)** The number of deaths among children under 5 years in 1990 is shown by country, **B)** The number of deaths among children under 5 years in 2010 is shown by country, **C)** The number of years lived with disability (YLD), a measure of the number of LRI episodes, among children under 5 years in 1990 is shown by country, **D)** The number of YLDs among children under 5 years in 2010 is shown by country.

A)

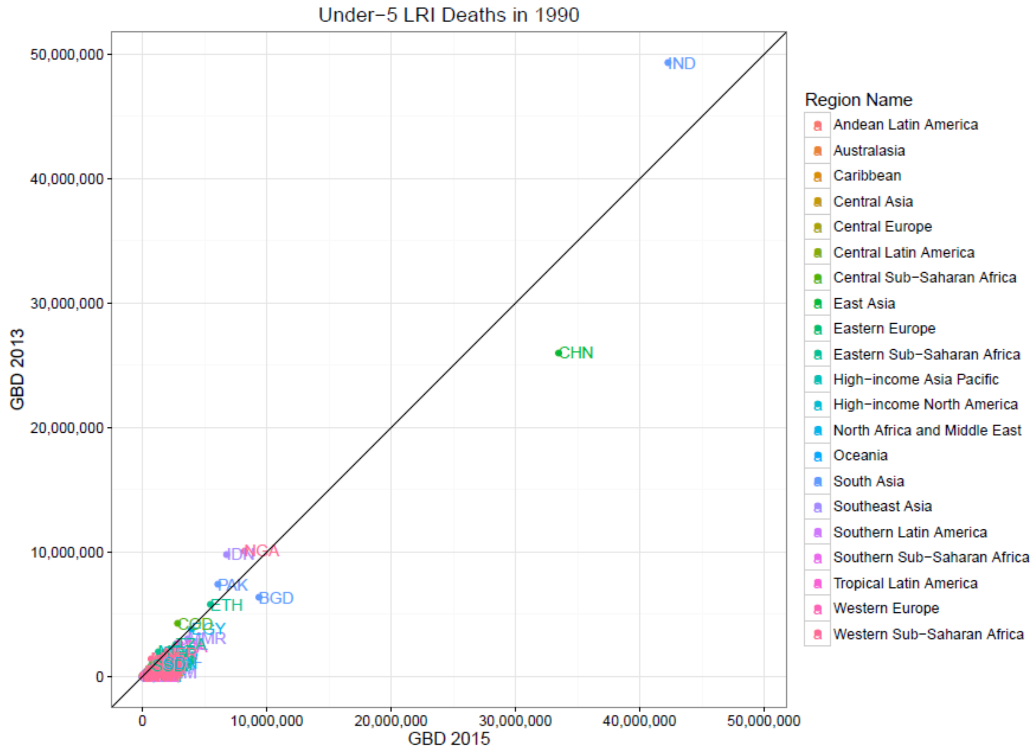

B)

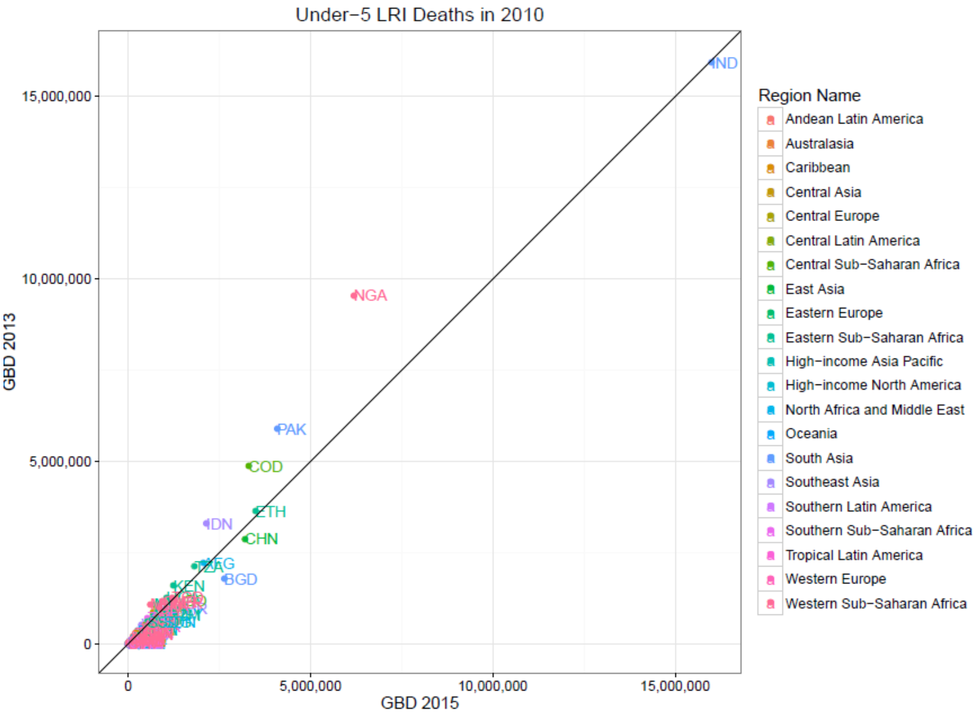

Under−5 LRI Deaths in 2010
GBD 2013
GBD 2015
15,000,000
10,000,000
5,000,000
0
0
5,000,000
10,000,000
15,000,000
NGA
PAK
COD
ETH
CHN
BGD
KEN
Region Name
Andean Latin America
Australasia
Caribbean
Central Asia
Central Europe
Central Latin America
Central Sub−Saharan Africa
East Asia
Eastern Europe
Eastern Sub−Saharan Africa
High−income Asia Pacific
High−income North America
North Africa and Middle East
Oceania
South Asia
Southeast Asia
Southern Latin America
Southern Sub−Saharan Africa
Tropical Latin America
Western Europe
Western Sub−Saharan Africa

C)

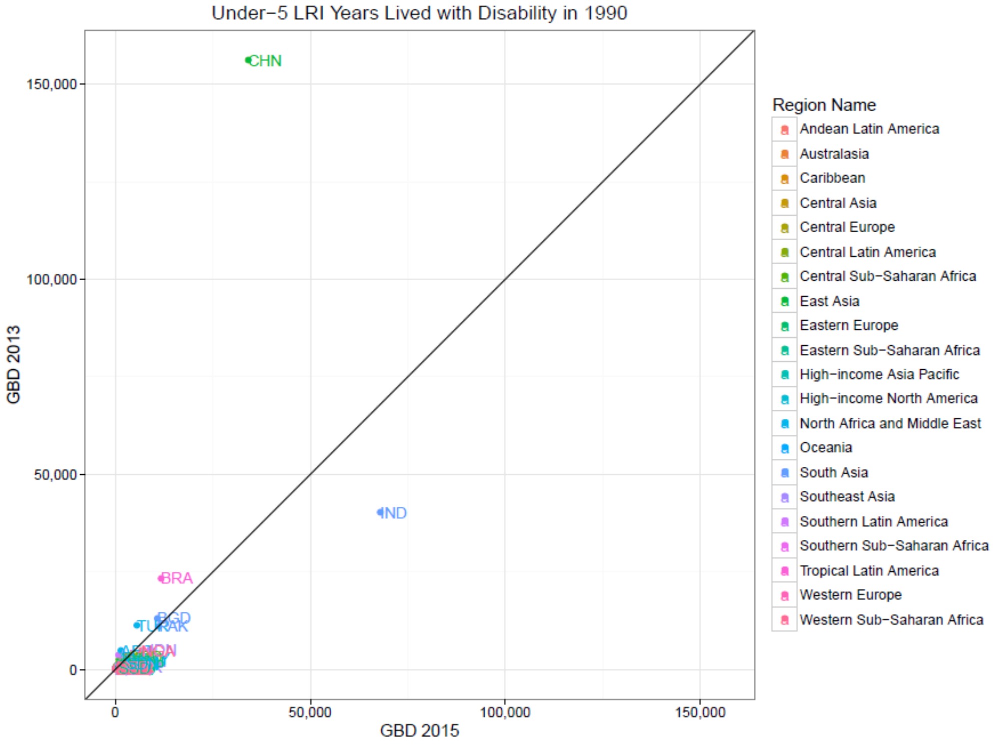

Under−5 LRI Years Lived with Disability in 1990
GBD 2013
GBD 2015
150,000
100,000
50,000
0
0
50,000
100,000
150,000
CHN
BRA
Region Name
Andean Latin America
Australasia
Caribbean
Central Asia
Central Europe
Central Latin America
Central Sub−Saharan Africa
East Asia
Eastern Europe
Eastern Sub−Saharan Africa
High−income Asia Pacific
High−income North America
North Africa and Middle East
Oceania
South Asia
Southeast Asia
Southern Latin America
Southern Sub−Saharan Africa
Tropical Latin America
Western Europe
Western Sub−Saharan Africa

D)

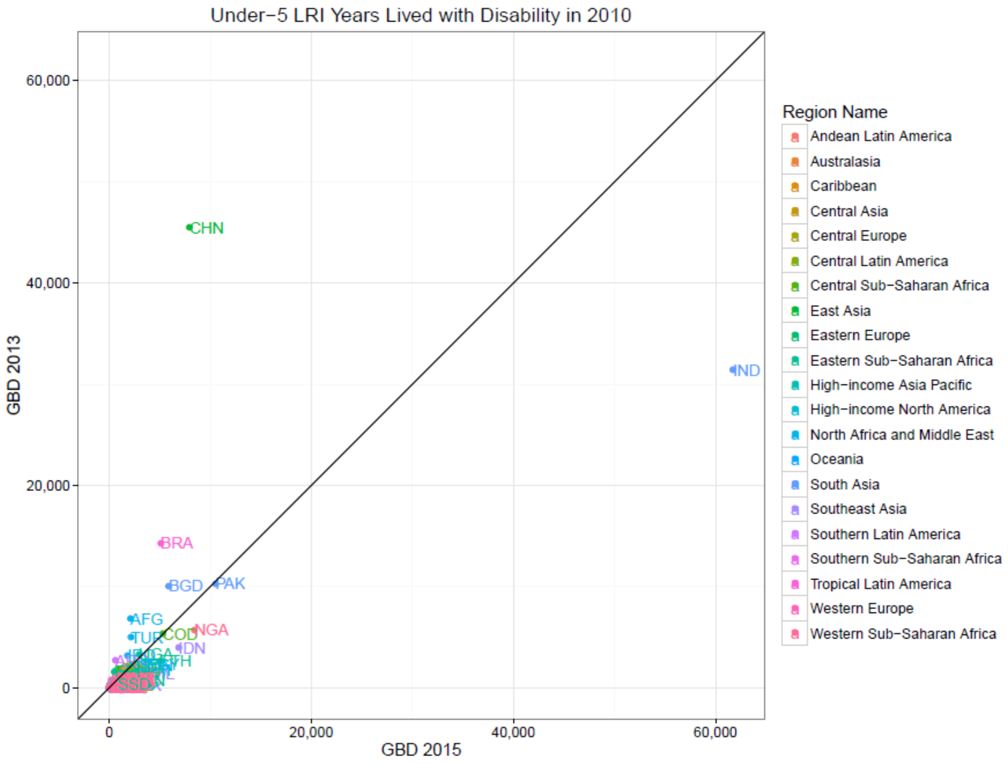

Under−5 LRI Years Lived with Disability in 2010
60,000
40,000
20,000
0
GBD 2013
0
20,000
40,000
60,000
GBD 2015
CHN
IND
BRA
BGD
PAK
AFG
NGA
COD
IDN
Region Name
Andean Latin America
Australasia
Caribbean
Central Asia
Central Europe
Central Latin America
Central Sub−Saharan Africa
East Asia
Eastern Europe
Eastern Sub−Saharan Africa
High−income Asia Pacific
High−income North America
North Africa and Middle East
Oceania
South Asia
Southeast Asia
Southern Latin America
Southern Sub−Saharan Africa
Tropical Latin America
Western Europe
Western Sub−Saharan Africa

Figure 6. Aetiologic attribution to under-5 LRI deaths in 2010. The percent of under-5 LRI deaths due to each aetiology is shown in the pie charts below. **A)** Attribution in GBD 2013.[20] **B)** Attribution in GBD 2015.

A)

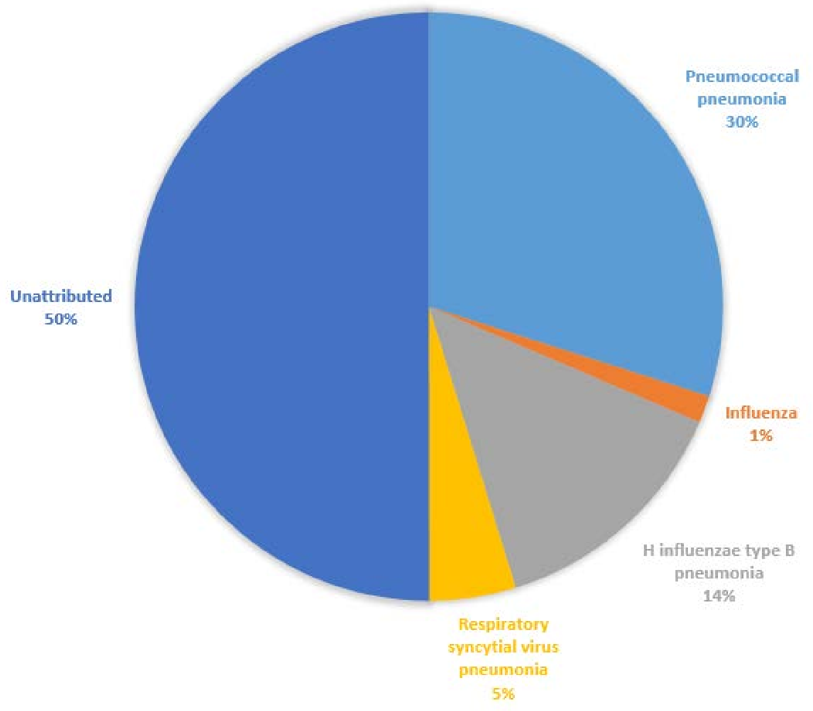

B)

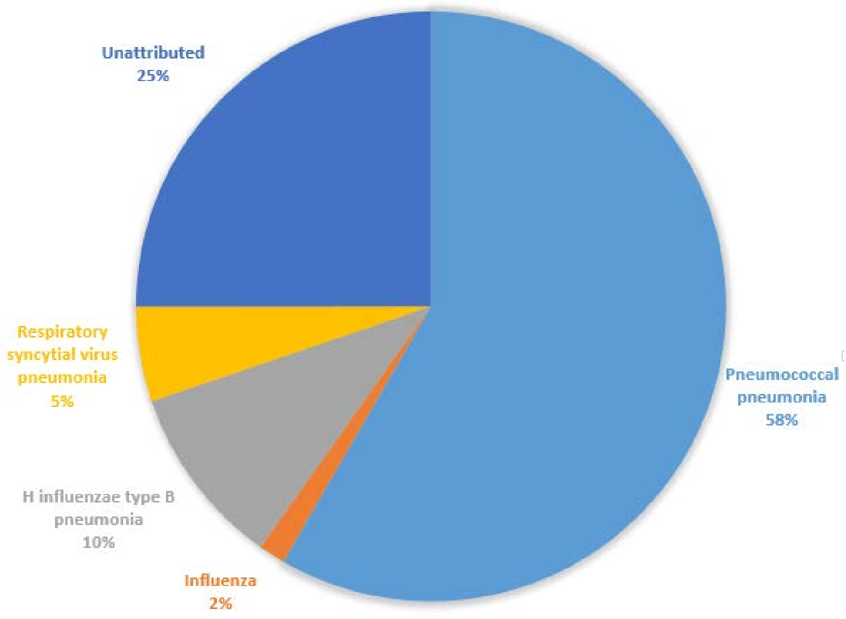

Comparison to WHO-MCEE

A comparison of the most recent estimates for etiologic attribution to under-5 LRI mortality between GBD 2015 and the CHERG-MCEE group is provided in the main text. The number of under-5 deaths by country in 2015 is shown in **Figure 7.**[22] The main differences are in India and Nigeria. Like much of Western sub-Saharan Africa, Nigeria has very little reliable cause-of-death data and estimates in this high burden country are influenced by regional trends and covariates. India is estimated at the subnational level in GBD 2015 and allows for much finer resolution and regional variation in LRI mortality estimates. A description of major differences between MCEE and GBD 2013 estimation can be found in manuscripts by Liu et al. 2015 and Vos et al. 2015.[23,24]

There is little agreement in aetiologic attribution in the year 2010 (most recent common year estimate) between GBD 2015 and the Child Health Epidemiology Research Group (CHERG), from which the MCEE developed,[25,26] besides the general order of burden. Influenza and RSV estimation strategies are markedly different between GBD and WHO-MCEE groups. In GBD 2015, we apply a counterfactual, severity- and case fatality-adjusted attributable fraction of LRI deaths to estimate the number of deaths due to influenza and RSV, while the WHO-MCEE group performed a meta-analysis of aetiology-specific incidence and applied several different approaches, based on case fatality and single study, cohort-based cause-specific mortality data.[27,28] By applying a counterfactual approach, we account for the frequency of detection of organisms in healthy individuals and by definition allow for co-infection between viral and bacterial causes leading to LRI episodes and deaths.[15,29] Although we share an estimation strategy for vaccine-preventable LRI aetiologies with CHERG,[8,10] a major difference is that in GBD 2015 we have introduced a correction factor for the vaccine efficacy against pneumococcal pneumonia that increases the attributable fraction for this aetiology. This correction factor is to account for the differential vaccine efficacy against invasive pneumococcal disease and pneumococcal pneumonia.

Table 8. Comparison with CHERG/MCEE.[26–28] The number of deaths (1000s) by aetiology is shown for the WHO-CHERG group and for GBD 2015 in 2010, the most recent shared year between groups.

Etiology		CHERG	GBD 2015
**H influenzae type B (Hib)**			
	Number	197 (27 to 345)	88.6 (0 to 168.6)
	Percent	15.7	9.9
**Influenza**			
	Number	137 (38 to 163)	12.9 (7.4 to 20.8)
	Percent	10.9	1.5
**Pneumococcal pneumonia**			
	Number	412 (218 to 569)	517 (309.9 to 678.1)
	Percent	32.8	58.4
**Respiratory syncytial virus**			
	Number*	66 to 199	46.1 (26.0 to 77.5)
	Percent	10.6 (midpoint)	5.2
**Total number of LRI deaths**		**1257 (1053 to 1483)**	**886.5 (826.6 to 949.9)**

***The reported ranges are not uncertainty, they are the low and high values form two separate approaches used to estimate aetiology mortality. The first is case-fatality from a meta-analysis (lower number) and the second is from a single-study cohort where the aetiology-specific mortality rate is calculated.**

Figure 7. Scatterplot of under-5 LRI deaths in 2015 comparing GBD 2015 and the WHO-MCEE group final estimates.[22]

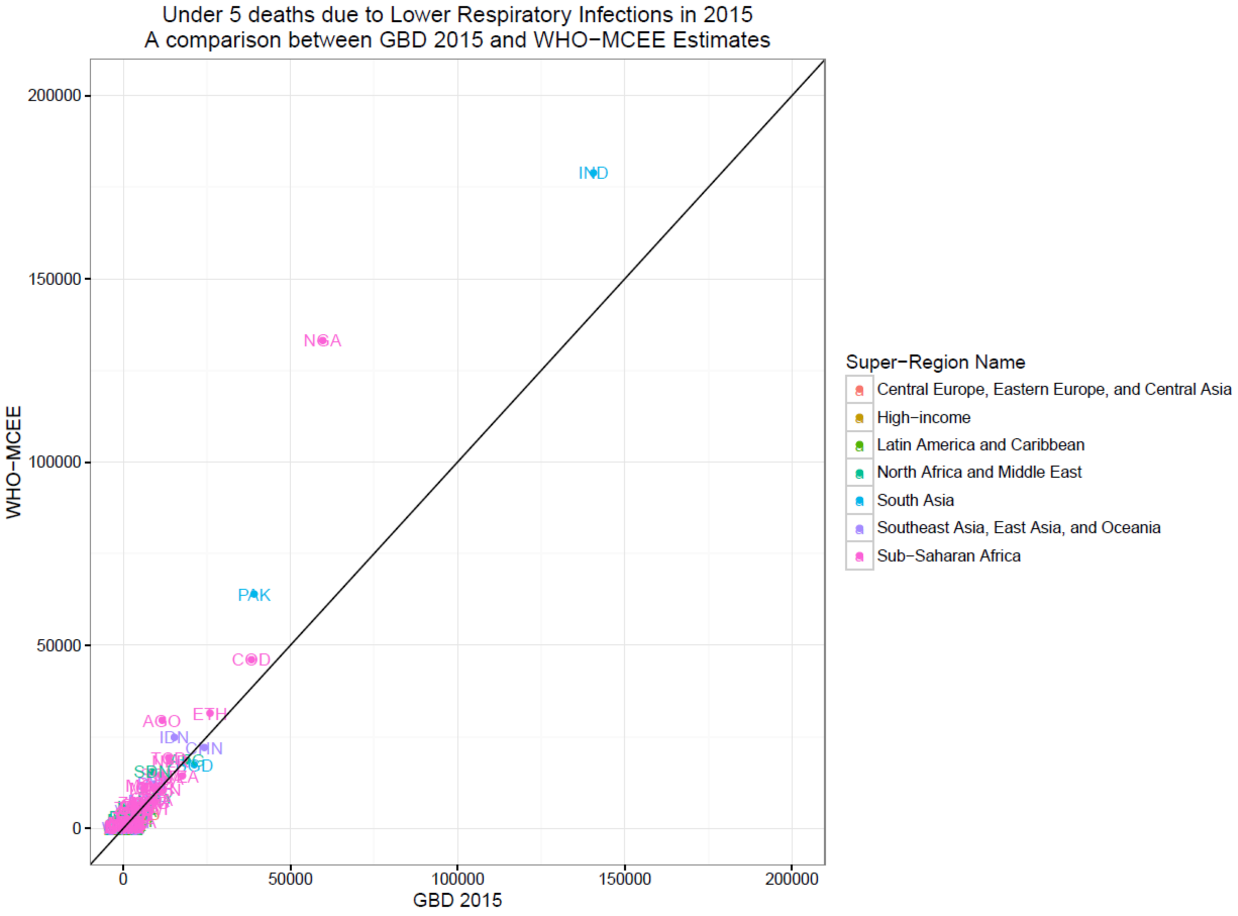

References

1 Naghavi M, Makela S, Foreman K, O’Brien J, Pourmalek F, Lozano R. Algorithms for enhancing public health utility of national causes-of-death data. *Popul Health Metr* 2010; **8**: 9.

2 GBD 2015 Mortality and Causes of Death Collaborators. Global, regional, and national life expectancy, all-cause and cause-specific mortality for 249 causes of death, 1980–2015: a systematic analysis for the Global Burden of Disease Study 2015. *Lancet* 2016; **388**: 1459–544.

3 Foreman KJ, Lozano R, Lopez AD, Murray CJ. Modeling causes of death: an integrated approach using CODEm. *Popul Health Metr* 2012; **10**: 1.

4 World Health Organization: Department of Child and Adolescent Health and Development. Handbook Integrated Management of Childhood Illness. 2005.

5 Salomon JA, Haagsma JA, Davis A, *et al.* Disability weights for the Global Burden of Disease 2013 study. *Lancet Glob Health* 2015; **3**: e712-723.

6 GBD 2015 Risk Factors Collaborators. Global, regional, and national comparative risk assessment of 79 behavioural, environmental and occupational, and metabolic risks or clusters of risks, 1990-2015: a systematic analysis for the Global Burden of Disease Study 2015. *Lancet Lond Engl* 2016; **388**: 1659–724.

7 Feikin DR, Scott JAG, Gessner BD. Use of vaccines as probes to define disease burden. *Lancet Lond Engl* 2014; **383**: 1762–70.

8 O’Brien KL, Wolfson LJ, Watt JP, *et al.* Burden of disease caused by Streptococcus pneumoniae in children younger than 5 years: global estimates. *Lancet Lond Engl* 2009; **374**: 893–902.

9 Johnson HL, Deloria-Knoll M, Levine OS, *et al.* Systematic evaluation of serotypes causing invasive pneumococcal disease among children under five: the pneumococcal global serotype project. *PLoS Med* 2010; **7**. DOI:10.1371/journal.pmed.1000348.

10 Watt JP, Wolfson LJ, O’Brien KL, *et al.* Burden of disease caused by Haemophilus influenzae type b in children younger than 5 years: global estimates. *Lancet Lond Engl* 2009; **374**: 903–11.

11 Swingler G, Fransman D, Hussey G. Conjugate vaccines for preventing Haemophilus influenzae type B infections. *Cochrane Database Syst Rev* 2007; : CD001729.

12 Lucero MG, Dulalia VE, Nillos LT, *et al.* Pneumococcal conjugate vaccines for preventing vaccine-type invasive pneumococcal disease and X-ray defined pneumonia in children less than two years of age. *Cochrane Database Syst Rev* 2009; : CD004977.

13 Bonten MJM, Huijts SM, Bolkenbaas M, *et al.* Polysaccharide conjugate vaccine against pneumococcal pneumonia in adults. *N Engl J Med* 2015; **372**: 1114–25.

14 Miettinen OS. Proportion of disease caused or prevented by a given exposure, trait or intervention. *Am J Epidemiol* 1974; **99**: 325–32.

15 Shi T, McLean K, Campbell H, Nair H. Aetiological role of common respiratory viruses in acute lower respiratory infections in children under five years: A systematic review and meta-analysis. *J Glob Health* 2015; **5**: 10408.

16 Bonten MJM, Huijts SM, Bolkenbaas M, *et al.* Polysaccharide conjugate vaccine against pneumococcal pneumonia in adults. *N Engl J Med* 2015; **372**: 1114–25.

17 Hill PC, Akisanya A, Sankareh K, *et al.* Nasopharyngeal carriage of Streptococcus pneumoniae in Gambian villagers. *Clin Infect Dis Off Publ Infect Dis Soc Am* 2006; **43**: 673–9.

18 Dowell SF, Garman RL, Liu G, Levine OS, Yang YH. Evaluation of Binax NOW, an assay for the detection of pneumococcal antigen in urine samples, performed among pediatric patients. *Clin Infect Dis Off Publ Infect Dis Soc Am* 2001; **32**: 824–5.

19 Scott JAG, Brooks WA, Peiris JSM, Holtzman D, Mulholland EK. Pneumonia research to reduce childhood mortality in the developing world. *J Clin Invest* 2008; **118**: 1291–300.

20 GBD 2013 Mortality and Causes of Death Collaborators. Global, regional, and national age-sex specific all-cause and cause-specific mortality for 240 causes of death, 1990-2013: a systematic analysis for the Global Burden of Disease Study 2013. *Lancet Lond Engl* 2015; **385**: 117–71.

21 Global Burden of Disease Study 2013 Collaborators. Global, regional, and national incidence, prevalence, and years lived with disability for 301 acute and chronic diseases and injuries in 188 countries, 1990-2013: a systematic analysis for the Global Burden of Disease Study 2013. *Lancet Lond Engl* 2015; **386**: 743–800.

22 WHO. Estimates for 2000-2015. http://www.who.int/ healthinfo/global_burden_disease/estimates_child_cod_2015/en/ (accessed Aug 25, 2016).

23 Liu L, Black RE, Cousens S, Mathers C, Lawn JE, Hogan DR. Causes of child death: comparison of MCEE and GBD 2013 estimates. *The Lancet* 2015; **385**: 2461–2.

24 Vos T, Flaxman AD, Naghavi M, *et al.* Years lived with disability (YLDs) for 1160 sequelae of 289 diseases and injuries 1990-2010: a systematic analysis for the Global Burden of Disease Study 2010. *Lancet Lond Engl* 2012; **380**: 2163–96.

25 JH Bloomberg School of Public Health. Maternal Child Epidemiology Estimation. http://www.jhsph.edu/research/centers-andinstitutes/ institute-for-international-programs/current-projects/ maternal-child-epidemiology-estimation/ (accessed Aug 26, 2016).

26 Walker CLF, Rudan I, Liu L, *et al.* Global burden of childhood pneumonia and diarrhoea. *Lancet Lond Engl* 2013; **381**: 1405–16.

27 Nair H, Brooks WA, Katz M, *et al.* Global burden of respiratory infections due to seasonal influenza in young children: a systematic review and meta-analysis. *Lancet Lond Engl* 2011; **378**: 1917–30.

28 Nair H, Nokes DJ, Gessner BD, *et al.* Global burden of acute lower respiratory infections due to respiratory syncytial virus in young children: a systematic review and meta-analysis. *Lancet Lond Engl* 2010; **375**: 1545–55.

29 Izadnegahdar R, Cohen AL, Klugman KP, Qazi SA. Childhood pneumonia in developing countries. *Lancet Respir Med* 2013; **1**: 574–84.
